# Treatment Strategies to Control Blood Pressure in People With Hypertension in Tanzania and Lesotho: A Randomized Clinical Trial

**DOI:** 10.1001/jamacardio.2024.5124

**Published:** 2025-01-29

**Authors:** Herry Mapesi, Martin Rohacek, Fiona Vanobberghen, Ravi Gupta, Herieth Ismael Wilson, Blaise Lukau, Alain Amstutz, Aza Lyimo, Josephine Muhairwe, Elizabeth Senkoro, Theonestina Byakuzana, Jacqueline Nkouabi, Geofrey Mbunda, Jamali Siru, Ayesha Tarr, Elsie Ramapepe, Madavida Mphunyane, Johanna Oehri, Valeriya Nemtsova, Xiaohan Yan, Moniek Bresser, Tracy Renée Glass, Daniel Henry Paris, Günther Fink, Winfrid Gingo, Niklaus Daniel Labhardt, Thilo Burkard, Maja Weisser

**Affiliations:** 1 Ifakara Health Institute, Ifakara Branch, Ifakara, United Republic of Tanzania; 2 Swiss Tropical and Public Health Institute, Basel, Switzerland; 3 University of Basel, Basel, Switzerland; 4 SolidarMed, Partnerships for Health, Maseru, Lesotho; 5 Division of Clinical Epidemiology, Department of Clinical Research, University Hospital Basel, Basel, Switzerland; 6 Oslo Centre for Biostatistics and Epidemiology, Oslo University Hospital, Oslo, Norway; 7 Bristol Medical School, University of Bristol, Bristol, United Kingdom; 8 St Francis Regional Referral Hospital, Ifakara, United Republic of Tanzania; 9 Tanzania Training Center for International Health, Ifakara, United Republic of Tanzania; 10 Agency for Development, Washington, DC; 11 Ministry of Health, Maseru, Lesotho; 12 Medical Outpatient and Hypertension Clinic, ESH Hypertension Centre of Excellence, University Hospital Basel, Basel, Switzerland; 13 Department of Cardiology, University Hospital Basel, Basel, Switzerland; 14 Division of Infectious Diseases, University Hospital Basel, Basel, Switzerland

## Abstract

**Importance:**

Hypertension is the primary cardiovascular risk factor in Africa. Recently revised World Health Organization guidelines recommend starting antihypertensive dual therapy; clinical efficacy and tolerability of low-dose triple combination remain unclear.

**Objectives:**

To compare the effect of 3 treatment strategies on blood pressure control among persons with untreated hypertension in Africa.

**Design, Setting, and Participants:**

This was an open-label, parallel, 3-arm randomized clinical trial to evaluate noninferiority of a strategy starting 2 pills vs full-dose monotherapy with stepped escalation (noninferiority margin 10%) and superiority of starting low-dose 3 pills vs monotherapy allowing for monthly up titration. Recruitment lasted from March 5, 2020, to March 30, 2022. The setting was 2 hospitals in rural Lesotho and Tanzania. Participants included nonpregnant Black African individuals 18 years and older with uncomplicated, untreated hypertension (standardized office blood pressure ≥140 mm Hg systolic or ≥90 mm Hg diastolic).

**Interventions:**

Participants were randomized 2:2:1 to stepped monotherapy (amlodipine, 10 mg, with escalation to add hydrochlorothiazide if needed), 2-pill strategy (amlodipine, 5 mg; losartan, 50 mg), or 3-pill strategy (amlodipine, 2.5 mg; losartan, 12.5 mg; hydrochlorothiazide, 6.25 mg). Drugs were up titrated monthly until reaching the target blood pressure (≤ 130/80 mm Hg for participants aged <65 years; ≤140/90 mm Hg for those aged ≥65 years).

**Main Outcomes and Measures:**

Proportion of participants reaching target blood pressure at 12 weeks.

**Results:**

Of 1761 participants screened, 1268 were enrolled (median [IQR] age, 54 [45-65] years; 914 female [72%]), with 505 in the monotherapy cohort, 510 in the 2-pill cohort, and 253 in the 3-pill cohort. In noninferiority analyses, 207 of 370 participants (56%) receiving the 2-pill strategy and 173 of 338 participants (51%) receiving the stepped monotherapy strategy achieved the blood pressure target (adjusted odds ratio [aOR], 1.18; 95% CI, 0.87-1.61), fulfilling noninferiority. In superiority analyses after multiple imputation for missing outcome data, 57% of participants receiving the 3-pill strategy, 55% receiving the 2-pill strategy, and 49% receiving the stepped monotherapy strategy reached the target blood pressure (aOR, 1.24; 95% CI, 0.94-1.63; *P* = .12 and aOR, 1.28; 95% CI, 0.91-1.79; *P* = .16 for the 2-pill and 3-pill vs stepped monotherapy strategies, respectively).

**Conclusions and Relevance:**

Results of this randomized clinical trial show that in 2 African settings, for adults with uncomplicated untreated hypertension, a strategy starting a 2-pill low-dose treatment was noninferior to starting stepped monotherapy. Two-pill and 3-pill low-dose strategies were not superior to stepped monotherapy. Wide CIs preclude the ability to rule out potentially clinically important effects of the additional pill strategies for hypertension control.

**Trial Registration:**

ClinicalTrials.gov Identifier: NCT04129840

## Key Points

**Question:**

Is a 2-pill antihypertensive strategy noninferior and is a low-dose 3-pill strategy superior to starting full-dose monotherapy with stepped escalation in controlling blood pressure in people with hypertension in rural Tanzania and Lesotho?

**Findings:**

In this 3-arm randomized clinical trial including 1268 individuals, starting 2 pills was noninferior and starting 3 pills was not superior compared with stepped monotherapy for blood pressure control at 12 weeks.

**Meaning:**

Results show that the 3 strategies led to similar blood pressure control; wide CIs preclude the ability to rule out clinically important effects of the additional pill strategies for hypertension control.

## Teaser

This randomized clinical trial investigates if a 2-pill antihypertensive strategy and a low-dose 3-pill strategy are superior to full-dose monotherapy with stepped escalation to control blood pressure in people with hypertension in rural Tanzania and Lesotho.

## Introduction

Hypertension affected 1.3 billion people worldwide in 2023.^1,2^ In Africa, the prevalence of hypertension is 25% to 30%, and 7% to 15% of individuals with hypertension have controlled blood pressure.^1,2,3,4^ Treatment recommendations for African people are extrapolated from studies including African American or Afro-Caribbean individuals with different genetic background, lifestyle, environmental factors, and drug availability.^5,6,7,8^

Until recently, the World Health Organization (WHO) recommended to start treatment with a diuretic or a calcium channel blocker (CCB) alone.^9,10^ After data from high-income settings demonstrated superiority of combination regimens,^11,12,13,14^ the WHO updated the recommendation to start an antihypertensive dual combination therapy in 2021.^15^ In Africa, Comparison of Three Combination Therapies in Lowering Blood Pressure in Black Africans (CREOLE) was the first large-scale clinical trial to compare combination antihypertensives in people with uncontrolled hypertension not receiving treatment or taking monotherapy. Amlodipine-containing combination therapies with either hydrochlorothiazide or perindopril were superior to perindopril plus hydrochlorothiazide.^16^ In Africa, combination therapies have not been compared with stepped-care approaches (ie, starting monotherapy and sequentially adding other drugs) in large trials. It remains unclear whether starting a low-dose triple combination is superior in terms of clinical efficacy and tolerability compared with a stepped-care approach.

We conducted the Identifying Strategies to Control Arterial Hypertension in Sub-Saharan Africa (coArtHA) randomized clinical trial^17^ to compare 3 treatment strategies among persons with untreated hypertension in Lesotho and Tanzania. We hypothesized that a 2-pill strategy starting a CCB and an angiotensin II receptor blocker (ARB) at lower doses would be noninferior compared with high-dosed CCB monotherapy as an initial strategy, with stepped escalation to add hydrochlorothiazide if needed, and that a low-dose 3-pill strategy containing low-dose CCB, ARB, and hydrochlorothiazide would be superior for blood pressure control, reduce adverse effects, and prevent hypertension-mediated organ damage compared with starting CCB monotherapy.^12,14^

## Methods

### Study Design

The coArtHA trial was an investigator-initiated, parallel, open-label, 3-arm randomized clinical trial conducted at the St Francis Regional Referral Hospital, Ifakara, in rural Tanzania and at the Mokhotlong District Hospital, Mokhotlong, in rural Lesotho. This trial was approved by the Ethikkomission Nordwest und Zentralschweiz, Switzerland, the institutional review board of the Ifakara Health Institute, the National Institute for Medical Research, the Tanzania Medicines and Medical Devices Authority, and the National Health Research and Ethics Committee, Ministry of Health of Lesotho. We used a superiority design for the comparison between starting a 3-pill strategy (CCB/ARB/hydrochlorothiazide) and a stepped CCB monotherapy strategy (CCB/hydrochlorothiazide),^9^ and a noninferiority design for the comparison of starting a 2-pill strategy (CCB/ARB).

 Participants gave written informed consent for screening and enrollment. The protocol (Supplement 1) and statistical analysis plan (Supplement 2) have been published previously.^17^ An independent data monitoring committee evaluating interim efficacy and safety data in July 2021 recommended continuation. This study followed the Consolidated Standards of Reporting Trials (CONSORT) reporting guidelines.^18,19^

### Participants

Participants were recruited from general outpatient and HIV departments and through community-based prescreening events. Black African individuals 18 years and older with confirmed uncomplicated untreated hypertension (ie, no antihypertensive medication within the last month) were eligible. Hypertension was determined by standardized office blood pressure greater than or equal to 140/90 mm Hg using the mean of the last 2 of 3 measurements spaced 1 to 2 minutes apart^12^ with an Omron M6 Comfort (HEM-7321-E)^20^ blood pressure device (Omron Corporation). Exclusion criteria were current hospitalization, acute disease, blood pressure greater than or equal to 180/110 mm Hg with acute headache or chest pain, a cardiovascular event in the last month, clinical signs of hypertension-mediated organ damage, creatinine clearance less than or equal to 30 mL/min/1.73 m^2^ (to convert to milliliters per second per meter squared, multiply by 0.0167), a positive pregnancy test, or inability to attend follow-up visits.

### Randomization and Masking

Participants were randomized in a 2:2:1 ratio to the stepped monotherapy, 2-pill, and 3-pill strategies, respectively. Randomization was stratified by site, HIV status, and age (<65 years and ≥65 years) using permuted blocks with varying block sizes. Allocation was concealed using opaque, sealed envelopes prepared by independent persons based on a randomization list generated by an independent statistician. The first 5 randomizations in each stratum were checked in real time; subsequent regular checks were performed to ensure randomization sequence was respected. Investigators and participants were not blinded to treatment allocation.

### Intervention

Participants randomized to the stepped monotherapy cohort followed the 2019 WHO treatment algorithm, namely, amlodipine, 10 mg, once daily with the addition of hydrochlorothiazide if the target blood pressure was not reached^8,9,11^ (eTable 1 in Supplement 3). Participants randomized to the 2-pill cohort started with amlodipine, 5 mg, and losartan, 50 mg, once daily, and those randomized to the 3-pill cohort were started with amlodipine, 2.5 mg, hydrochlorothiazide, 6.25 mg, and losartan, 12.5 mg, once daily with subsequent dose escalation in all groups if blood pressure was above target.

### Outcomes

The primary end point was the proportion of participants achieving target blood pressure at 12 weeks. The blood pressure target was less than or equal to 130/80 mm Hg for participants younger than 65 years and less than or equal to 140/90 mm Hg for those 65 years and older according to the 2018 European Society of Cardiology (ESC) and the European Society of Hypertension (ESH) guidelines for the management of hypertension.^12^ Secondary outcomes were target blood pressure attainment at 4, 8, and 24 weeks, changes in blood pressure, time to target blood pressure attainment, the proportion of participants with treatment adjustments, the number of adjustments per participant, and the proportion of participants with poor adherence (<90% pill count, self-reported missing pills ≥2 times in the last month, or drug intake different from prescription corresponding to <90% adherence) at 12 weeks. Further secondary outcomes over 24 weeks were loss to follow-up, treatment discontinuation for any reason, kidney impairment (using the Kidney Disease Improving Global Outcomes classification^21^ Chronic Kidney Disease Epidemiology Collaboration equation^22^ and albumin-creatinine ratio^21^), left ventricular hypertrophy based on left ventricular mass index^23^ by focused echocardiography if available, or Multi-Ethnic Study of Atherosclerosis Index,^24^ major cardiovascular events (ie, death, stroke, myocardial infarction, heart failure, end-stage kidney disease), 1 or higher grade three-quarter adverse event according to the Common Terminology Criteria for Adverse Events,^25^ and serious adverse events.

### Procedures

Screened individuals received history taking, physical examination, serum creatinine level test (ISTAT [Abbott Diagnostics]), rapid HIV test (Bioline [Abbott Diagnostics]) for those not known to live with HIV or not tested within the last 3 months, and urine beta human chorionic gonadotropin pregnancy test in women aged 18 to 45 years. Enrolled participants received full blood cell counts, serum alanine aminotransferase level test, urine dipstick, urine albumin-creatinine ratio, a 12-lead electrocardiogram (Schiller AT-1 electrocardiograph [Schiller Baar Switzerland]), and a focused echocardiography (Lumify [Philips]). Electrocardiograms and focused echocardiograms were analyzed under supervision of a board-certified cardiologist, supported by a US Food and Drug Administration–approved deep-learning workflow (Us2.ai Singapore).^26^ After randomization, participants received prepacked, open-labeled study medication for free until the next visit. Medication brand was selected according to local availabilities. Medication was purchased locally from national governmental drug supply stores or trusted private sector vendors. Medication was stored at the hospital pharmacy in temperature-controlled rooms or at the study room for daily dispensing.

Follow-up visits at 4, 8, 12, and 24 weeks after enrollment included standardized blood pressure measurement, adherence assessment (pill count and self-report), physical examination, assessment of adverse events, and pregnancy testing in women aged 18 to 45 years. Women with a positive pregnancy test were switched to an antihypertensive regimen as per local guidelines. At week 24, blood and urine tests, electrocardiography, and focused echocardiography were repeated. Participants who missed their appointment were tracked within a week of the missed appointment.

### Statistical Analysis

 Assuming blood pressure target attainment in the monotherapy strategy of 40%, an improvement of 15% (2-sided α of .05) for the superiority comparison between the triple and monotherapy strategy, and a noninferiority margin of 10% (1-sided α of .025) for the noninferiority comparison between the 2-pill and stepped monotherapy strategies, a sample size of 431 participants in each of the 2-pill strategy and stepped monotherapy strategy and 216 in the 3-pill strategy were required for a power of 85% and 95% for the noninferiority and superiority comparisons, respectively. Allowing for 15% loss to follow-up, we aimed to enroll 1268 participants. Noninferiority was compatible with the lower bound of the 95% CI for the odds ratio (OR) exceeding 0.64. No adjustments were made for multiple testing (eAppendix in Supplement 3).

Outcomes were analyzed within an estimand framework^27^ (eTables 2 and 3 in Supplement 3) by randomization allocation. For binary outcomes, we used logistic regression models adjusted for baseline systolic and diastolic blood pressure and randomization stratification factors (ie, country, HIV status, age). We estimated risk differences using the delta method.^28^ Continuous outcomes were assessed using linear regression models, and time-to-event outcomes were assessed using Kaplan-Meier estimation and Cox proportional hazards models. Effect modification of the primary outcome by country and HIV status was assessed a priori (and age, post hoc) by including an interaction accordingly. For blood pressure–related outcomes, missing data were imputed using chained equations with 50 imputed datasets separately by strategy.^29^ We performed predefined sensitivity analyses for the primary outcome. In post hoc analyses, we assessed the essential blood pressure target (≤140/90 mm Hg) regardless of age.^13^

Hypertension-mediated organ damage was based on the surrogate markers of kidney impairment and signs of left ventricular hypertrophy (eTable 4 in Supplement 3). Major cardiovascular events included heart failure, ischemic heart disease, stroke, and kidney failure (eTable 5 in Supplement 3). All *P* values were 2-sided, and data were analyzed using Stata software, version 16 (StataCorp).

## Results

### Participant Characteristics

**Figure 1.**
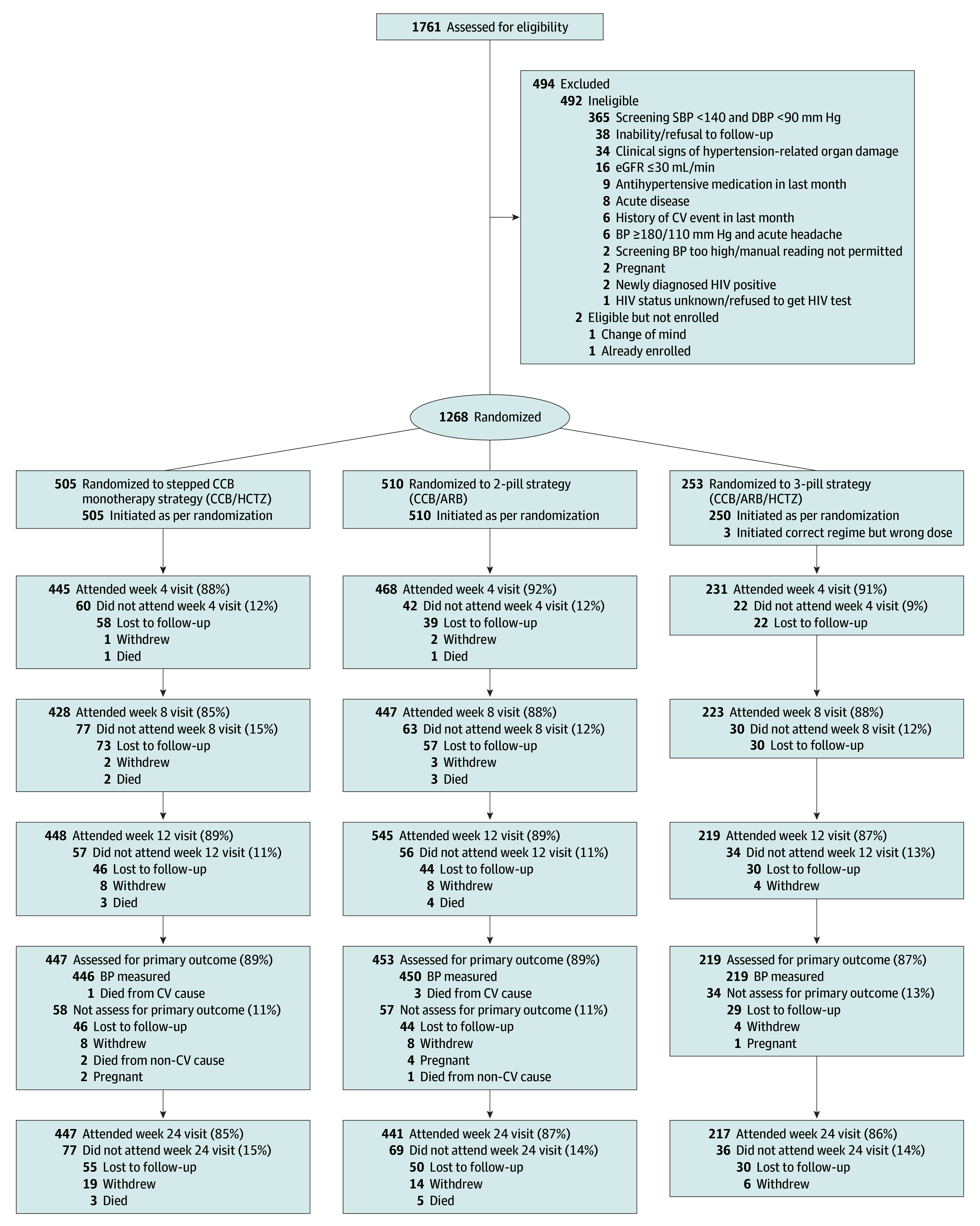
Participants Flowchart ARB indicates angiotensin II receptor blocker; BP, blood pressure; CCB, calcium channel blocker; CV, cardiovascular; DBP, diastolic blood pressure; eGFR, estimated glomerular filtration rate; HCTZ, hydrochlorothiazide; SBP, systolic blood pressure.

From March 5, 2020, to March 30, 2022, 1761 persons were screened, and 1268 were enrolled (median [IQR] age, 54 [45-65] years; 914 female [72%]; 354 male [28%]) and randomized. The main reason for exclusion was normalized values in the standardized office blood pressure measurement (n = 365) (Figure 1). At randomization, 505, 510, and 253 participants were allocated to the stepped monotherapy cohort, 2-pill cohort, and 3-pill cohort, respectively. Baseline characteristics (Table 1) were well balanced across the 3 strategies, except those in the 3-pill cohort being older (≥65 years, 62 of 253 [25%] vs 2-pill cohort, 124 of 510 [24%] and stepped monotherapy cohort, 123 of 505 [24%]), less likely female (171 of 253 [68%] vs 2-pill cohort, 364 of 510 [71%] and stepped monotherapy cohort, 379 of 505 [75%]), and more likely to have a history of hypertension (121 of 253 [48%] vs 2-pill cohort, 222 of 510 [44%] and stepped monotherapy cohort, 212 of 505 [42%]), compared with the stepped monotherapy and 2-pill cohorts. All but 3 participants started antihypertensive therapy according to the assigned treatment strategy. Visit attendance was 90%, 87%, 88%, and 86% at weeks 4, 8, 12, and 24, respectively, across strategies.

**Table 1.** Baseline Characteristics by Randomization Strategy

Characteristic	No. (%)		
	Stepped monotherapy strategy (CCB/HCZT) (n = 505)	Two-pill strategy (CCB/ARB) (n = 510)	Three-pill strategy (CCB/ARB/HCTZ) (n = 253)
Site			
Ifakara	266 (53)	268 (53)	134 (53)
Mokhotlong	239 (47)	242 (47)	119 (47)
Age, y^a^			
<45	146 (29)	125 (25)	57 (23)
45-64	236 (47)	261 (51)	134 (53)
≥65	123 (24)	124 (24)	62 (25)
Sex			
Female	379 (75)	364 (71)	171 (68)
Male	126 (25)	146 (29)	82 (32)
HIV status			
Positive^b^	183 (36)	185 (36)	93 (37)
Negative	322 (64)	325 (64)	160 (63)
Marital status			
Single	33 (7)	41 (8)	10 (4)
Married/cohabiting	275 (54)	265 (52)	140 (55)
Divorced/separated/widow(er)	197 (39)	204 (40)	103 (41)
Occupation			
None	161 (32)	165 (32)	73 (29)
Employed	195 (39)	184 (36)	88 (35)
Farmer/fisher	84 (17)	82 (16)	53 (21)
Small business	19 (4)	20 (4)	10 (4)
Farmer/fisher and small business	46 (9)	59 (12)	29 (11)
Education			
None	54 (12)	43 (10)	34 (15)
Some primary	125 (28)	134 (30)	60 (26)
Completed primary	182 (40)	185 (41)	91 (40)
Beyond primary	91 (20)	90 (20)	45 (20)
Missing	53 (10)	58 (11)	23 (9)
Health insurance	42 (8)	48 (9)	25 (10)
Toxic habits/CV risk factors			
Alcohol consumption (AUDIT score)			
No/mild (0-7)	485 (96)	494 (97)	243 (96)
Hazardous (8-15)	18 (4)	15 (3)	6 (2)
Harmful (≥16)	2 (<1)	1 (<1)	4 (2)
Cigarette smoking			
Current	38 (8)	28 (5)	16 (6)
Previous	30 (6)	43 (8)	28 (11)
Never	437 (87)	439 (86)	209 (83)
Family history of CV events^c^	53 (10)	42 (8)	22 (9)
Past medical history			
Previous diagnosis of hypertension^d^	212 (42)	222 (44)	121 (48)
History of tuberculosis^e^	46 (9)	59 (12)	24 (9)
Diabetes^f^	7 (1)	13 (3)	5 (2)
Other chronic diseases	15 (3)	12 (2)	9 (4)
Participants with ≥1 comedication at enrollment^g^	228 (45)	241 (47)	124 (49)
Current signs and symptoms			
Any complaints	170 (34)	191 (37)	92 (36)
BMI^h^			
<18.5	19 (4)	18 (4)	7 (3)
18.5-<25	188 (37)	185 (36)	106 (42)
25-<30	162 (32)	154 (30)	65 (26)
≥30	136 (27)	153 (30)	75 (30)
Working functional status	491 (97)	496 (97)	245 (97)

Abbreviations: ARB, angiotensin II receptor blocker; AUDIT, Alcohol Use Disorders Identification Test; BMI, body mass index; CCB, calcium channel blocker; CV, cardiovascular; HCTZ, hydrochlorothiazide.

^a^ One participant was wrongly stratified to age group younger than 65 years at randomization due to a typing mistake in his age and was corrected later to be 65 years and older for reporting and analysis.

^b^ All participants positive for HIV were taking antiretroviral treatment.

^c^ Heart attack or stroke in parents or siblings with event younger than 60 years. Not known for 22 participants in the monotherapy cohort, 26 in the 2-pill cohort, and 10 in the 3-pill cohort.

^d^ Never measured in 2 participants in the stepped CCB monotherapy cohort, 3 in the 2-pill cohort, and 1 in the 3-pill cohort.

^e^ One participant in each of the stepped CCB monotherapy cohort and 3-pill cohort did not know.

^f^ Never measured in 44 participants in the stepped CCB monotherapy cohort, 50 in the 2-pill cohort, and 22 in the 3-pill cohort.

^g^ 61% Being antiretroviral drugs.

^h^ Calculated as weight in kilograms divided by height in meters squared.

### Primary Outcome

Under a per-protocol approach for the noninferiority comparison of the 2-pill vs stepped monotherapy strategy, 370 participants (73%) in the 2-pill cohort and 338 participants (67%) in the stepped monotherapy cohort were included. Of those participants, 207 (56%) and 173 (51%), respectively, met the primary outcome, yielding an adjusted OR (aOR) of 1.18 (95% CI, 0.87-1.61) (Table 2 and eTable 6 in Supplement 3).^28^ As the lower bound of the 95% CI exceeded the predefined noninferiority threshold of 0.64, noninferiority was demonstrated, allowing assessment for superiority.

**Table 2.** Primary and Secondary Outcomes

Outcome	Stepped monotherapy strategy (CCB/HCZT) (n = 505)	Two-pill strategy (CCB/ARB) (n = 510)	Three-pill strategy (CCB/ARB/HCTZ) (n = 253)
**Primary outcome**			
Per-protocol approach for noninferiority comparison of 2-pill vs stepped monotherapy strategy (complete case analysis)			
Included, No.	338	370	NA
Reached primary outcome,^a^ No. (%)	173 (51)	207 (56)	NA
Odds ratio (95% CI)^b^	1 [Reference]	1.18 (0.87 to 1.61)	NA
Intention-to-treat approach for superiority comparisons			
Complete case analysis			
Included, No.	447	453	219
Reached primary outcome,^a^ No. (%)	229 (51)	257 (57)	131 (60)
Odds ratio (95% CI)^b^	1 [Reference]	1.21 (0.92 to 1.60)	1.32 (0.94 to 1.86)
*P* value	NA	.17	.11
Multiple imputation analysis			
Included, No.	501	505	252
Reached primary outcome,^a^ %	49	55	57
Odds ratio (95% CI)^b^	1 [Reference]	1.24 (0.94 to 1.63)	1.28 (0.91 to 1.79)
*P* value	NA	.12	.16
Risk ratio (95% CI)^c^	1 [Reference]	1.10 (0.97 to 1.23)	1.11 (0.95 to 1.27)
Risk difference (95% CI)^c^	1 [Reference]	0.05 (−0.01 to 0.11)	0.06 (−0.02 to 0.13)
**Secondary outcomes**			
Blood pressure results and changes at week 12 (multiple imputation analysis)			
Result available,^d^ No.	500	502	252
SBP, mean (SE), mm Hg	125 (0.7)	126 (0.9)	124 (1.2)
SBP change, mean (SE), mm Hg	−28 (0.9)	−27 (0.9)	−29 (1.3)
DBP, mean (SE), mm Hg	83 (0.4)	83 (0.5)	81 (0.6)
DBP change, mean (SE), mm Hg	−17 (0.5)	−17 (0.5)	−19 (0.8)
Time to BP target attainment			
Observed to achieve target BP, No. (%)	380 (75)	392 (77)	205 (81)
Time to achieving target BP, median (95% CI), wk	8.7 (8.0 to 12.0)	8.0 (8.0 to 8.3)	11.3 (8.3 to 12.0)
Hazard ratio (95% CI)^e^^,^^f^	1 [Reference]	1.12 (0.97 to 1.28)	0.95 (0.80 to 1.12)
*P* value	NA	.13	.53
Treatment adaptations by week 12			
Participants with at least 1 treatment adaptation, No. (%)	366 (72)	360 (71)	197 (78)
Odds ratio (95% CI)^e^^,^^g^	1 [Reference]	0.95 (0.72 to 1.26)	1.48 (1.03 to 2.13)
*P* value	NA	.71	.04
Treatment adaptations per participant, median (IQR)	1 (0-2)	1 (0-2)	2 (1-2)
Incidence rate ratio (95% CI)^e^^,^^h^	1 [Reference]	0.95 (0.87 to 1.07)	1.06 (0.94 to 1.20)
*P* value	NA	.47	.35
Adherence at week 12			
Adherence data available, No.	446	454	219
≥90% Adherent, No. (%)	342 (77)	375 (83)	173 (79)
Odds ratio (95% CI)^e^^,^^g^	1 [Reference]	1.45 (1.04 to 2.02)	1.12 (0.75 to 1.66)
*P* value	NA	.03	.59
Lost to follow-up or stopped treatment over 24 wk			
LTFU or stopped treatment, No. (%)	138 (27)	111 (22)	61 (24)
Hazard ratio (95% CI)^e^^,^^f^	1 [Reference]	0.77 (0.60 to 0.99)	0.85 (0.63 to 1.15)
*P* value	NA	.04	.30
Hypertension-mediated organ damage by 24 wk			
Overall, No. (%)			
Result available, No.	422	439	212
Never	207 (49)	204 (46)	98 (46)
Newly occurring	32 (8)	30 (7)	16 (8)
Worsening	5 (1)	6 (1)	5 (2)
Resolving	81 (19)	81 (18)	46 (22)
No change	97 (23)	118 (27)	47 (22)
Missing	83 (16)	71 (14)	41 (16)
Left ventricular hypertrophy,^i^ No. (%)			
Never	357 (85)	374 (86)	180 (86)
Newly occurring	1 (0)	3 (1)	1 (0)
Resolving	30 (7)	29 (7)	18 (9)
No change	30 (7)	30 (7)	11 (5)
Missing	83 (16)	71 (14)	41 (16)
Kidney impairment,^j^ No. (%)			
Never	220 (53)	224 (53)	110 (53)
Newly occurring	35 (8)	33 (8)	17 (8)
Worsening	5 (1)	6 (1)	5 (2)
Resolving	81 (20)	69 (16)	37 (18)
No change	74 (18)	94 (22)	40 (19)
Missing	90 (18)	84 (16)	44 (17)
Major cardiovascular end points			
Participants with at least 1 major CV event, No. (%)	4 (1)	3 (1)	3 (1)
CV events, total No.	6	4	3
Angina pectoris	1	0	1
Heart failure	1	0	0
Stroke	2	1	1
CV-related death^k^	2	3	1
**Adverse events**			
Adverse events			
Participants with ≥1 adverse event, No. (%)	179 (35)	174 (34)	71 (28)
Participants with ≥1 grade ≥3 adverse event, No. (%)	27 (5)	22 (4)	9 (4)
Odds ratio (95% CI)^e^^,^^g^	1 [Reference]	0.75 (0.42-1.35)	0.60 (0.28-1.32)
*P* value	NA	.34	.21
Severe adverse events			
Participants with ≥1 severe adverse event,^l^ No. (%)	8 (2)	7 (1)	6 (2)
Odds ratio (95% CI)^e^^,^^g^	1 [Reference]	0.83 (0.30 to 2.33)	1.36 (0.46 to 4.02)
*P* value	NA	.72	.57
Adverse events, total No.	303	303	126
Grade ≥3 events, No. (%)	35 (12)	26 (9)	12 (10)
Probable/definite relation to study drug, No. (%)	80 (26)	72 (24)	13 (10)
Possible/probable/definite relation to amlodipine, No. (%)	95 (31)	71 (23)	8 (6)
Possible/probable/definite relation to losartan, No. (%)	1 (0)	2 (1)	2 (2)
Possible/probable/definite relation to HCTZ, No. (%)	7 (2)	1 (0)	1 (1)

Abbreviations: ARB, angiotensin II receptor blocker; BP, blood pressure; CCB, calcium channel blocker; CV, cardiovascular; DBP, diastolic blood pressure; eGFR, estimated glomerular filtration rate; HCTZ, hydrochlorothiazide; KDIGO, Kidney Disease: Improving Global Outcomes; LTFU, lost to follow-up; NA, not applicable; SBP, systolic blood pressure.

^a^ Percentages of those included in the respective analysis.

^b^ Logistic regression model, adjusted for site, HIV status, age (linear), baseline SBP (linear), and baseline DBP (linear).

^c^ CI estimated using delta method.^28^

^d^ Values were multiply imputed for those who did not have BP measured, except for those pregnant or who died, hence values are not total number randomized.

^e^ Adjusted for site, HIV status, age (linear), baseline SBP (linear), and baseline DBP (linear).

^f^ Cox proportional hazards model.

^g^ Logistic regression model.

^h^ Poisson regression model. Test for overdispersion, *P* >.99; therefore, Poisson model appropriate.

^i^ Hypertension-mediated organ damage was defined as any new or worsening sign for (1) new kidney impairment to KDIGO stage 3a or higher or worsening to a higher grade or new albumin-creatinine ratio greater than or equal to 1 or worsening to a higher stage; (2) left ventricular hypertrophy: new left ventricular mass/body surface area greater than 95 g/m^2^ in women, greater than 115 g/m^2^ in men or if no echocardiography available new fulfillment of Multi-Ethnic Study of Atherosclerosis Index left ventricular hypertrophy criteria (eTable 4 in Supplement 3).

^j^ Using the most pathologic evaluation of eGFR and albumin-creatinine ratio, if both available or in case of missingness eGFR or albumin-creatinine ratio alone.

^k^ Five died of stroke, 1 of kidney failure.

^l^ One severe adverse event each.

**Figure 2.**
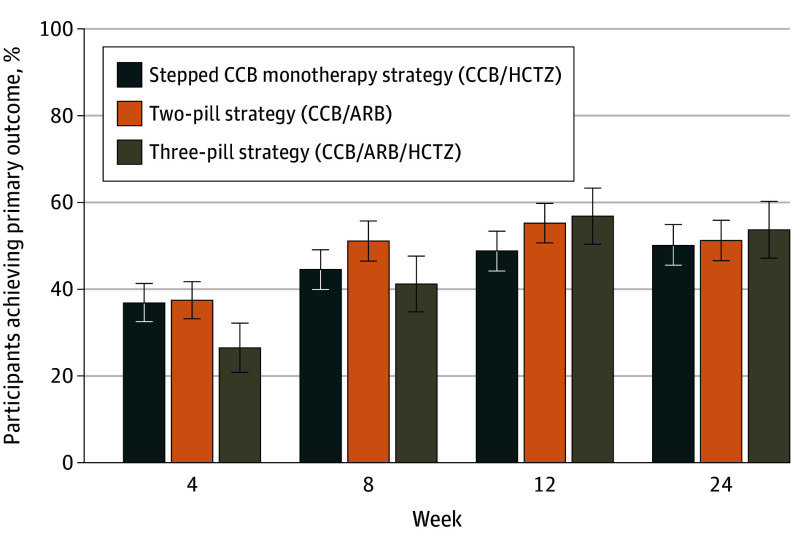
Target Blood Pressure Attainment Analysis based on multiply imputed data. The primary outcome of target blood pressure (defined as systolic blood pressure [SBP] ≤130 mm Hg and diastolic blood pressure [DBP] ≤80 mm Hg for participants aged <65 years and SBP ≤140 mm Hg and DBP ≤90 mm Hg for participants aged ≥65 years) is shown in bars at week 12 and additionally for secondary outcomes at week 4, 8, and 24. ARB indicates angiotensin II receptor blocker; CCB, calcium channel blocker; HCTZ, hydrochlorothiazide.

In the complete case analysis for superiority comparisons, the primary end point at 12 weeks was assessed in 447 participants (89%) receiving the stepped monotherapy strategy, 453 participants (89%) receiving the 2-pill strategy, and 219 participants (87%) receiving the 3-pill strategy, of whom 229 (51%), 257 (57%), and 131 (60%), respectively, attained the target blood pressure (Tables 2 and eTable 6 in Supplement 3).^28^ In the multiple imputation analysis, the proportions of participants achieving the primary outcome were 49% in the stepped monotherapy cohort, 55% in the 2-pill cohort, and 57% in the 3-pill cohort (Table 2 and Figure 2).^28^ aORs were 1.24 (95% CI, 0.94-1.63; *P* = .12) for the 2-pill strategy and 1.28 (95% CI, 0.91-1.79; *P* = .16) for the 3-pill strategy vs stepped monotherapy strategy, with risk differences of 0.05 (95% CI, −0.01 to 0.11) and 0.06 (95% CI, −0.02 to 0.13), respectively. Results were robust to sensitivity analyses (eTable 6 in Supplement 3). There was no effect modification by either country or HIV status (eTable 7 in Supplement 3).

### Secondary Outcomes

**Figure 3.**
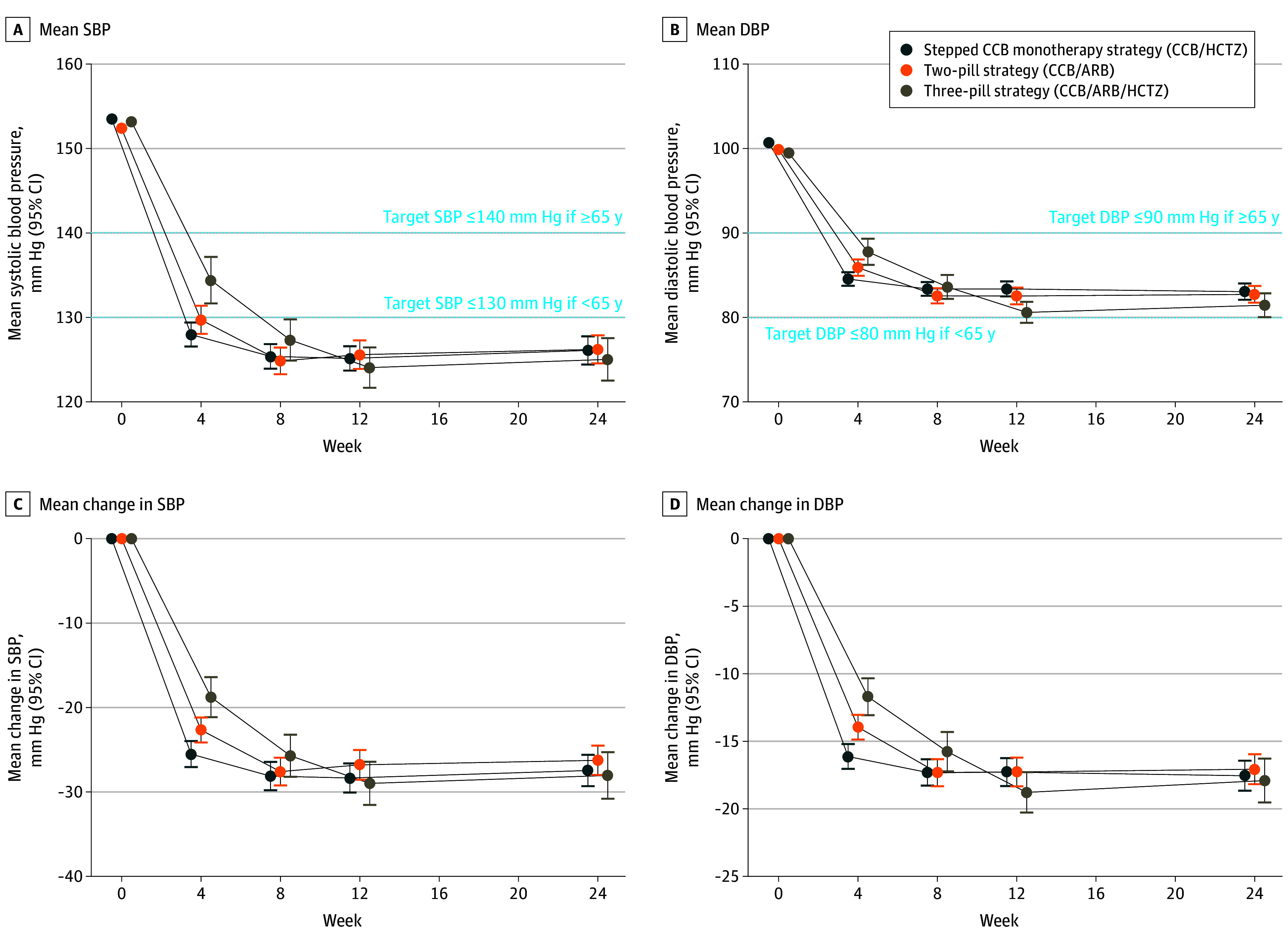
Blood Pressure Response Analysis based on multiply imputed data. The left upper graph shows mean systolic blood pressure (SBP) values and SD at the study visits (week 4, 8, 12, and 24), the right upper graph shows mean diastolic blood pressure (DBP) values and SD with targets for the different age populations as dashed lines. The lower 2 diagrams show changes of SBP and DBP at the respective time points. ARB, indicates angiotensin II receptor blocker; CCB, calcium channel blocker; HCTZ, hydrochlorothiazide.

In the stepped monotherapy strategy, 2-pill strategy, and 3-pill strategy, mean (SE) changes in the systolic blood pressure from baseline to 12 weeks were −28 (0.9) mm Hg, −27 (0.9) mm Hg, and −29 (1.3) mm Hg, respectively, and mean (SE) changes in diastolic blood pressure were −17 (0.5) mm Hg, −17 (0.5) mm Hg, and −19 (0.8) mm Hg, respectively (Table 2).^28^ Blood pressure dropped fastest with the stepped monotherapy strategy but was similar across strategies by week 24 (Figure 3 and eTables 8 and 9 in Supplement 3). Higher proportions of participants reached the systolic blood pressure target (74%-76%) at 12 weeks, compared with diastolic blood pressure (54%-63%) (eTable 8 in Supplement 3). The proportions of participants achieving target blood pressure at weeks 4, 8, and 24 were 37%, 44%, and 50% in the stepped monotherapy cohort; 37%, 51%, and 51% in the 2-pill cohort, and 26%, 41%, and 54% in the 3-pill cohort (Figure 2, Figure 3, and eTables 8 and 10 in Supplement 3). The median time to achieving target blood pressure was 8.7 (95% CI, 8.0-12.0 weeks), 8.0 (95% CI, 8.0-8.3) weeks, and 11.3 (95% CI, 8.3-12.0) weeks in the stepped monotherapy, 2-pill, and 3-pill cohorts, respectively, with no differences between strategies (Table 2 and eFigure 1 in Supplement 3).^28^

Prespecified treatment escalations by week 4 due to unmet blood pressure target were indicated in 264 participants (52%) in the stepped monotherapy cohort, 279 participants (55%) in the 2-pill cohort, and 164 participants (65%) in the 3-pill cohort, followed by a second treatment escalation by week 8 in 157 participants (31%) in the stepped monotherapy cohort, 144 participants (28%) in the 2-pill cohort, and 101 participants (40%) in the 3-pill cohort (eTable 11 and eFigure 2 in Supplement 3). At week 12, 25% (114 of 448 participants), 31% (140 of 454 participants), and 44% (97 of 219 participants) receiving the stepped monotherapy, 2-pill, and 3-pill strategies reached maximal prescription according to their randomization, and the numbers of participants with at least 1 treatment adaptation by week 12 were 366 (72%), 360 (71%), and 197 (78%) in each group, respectively (Table 2).^28^ Conversely, 93 participants (18.4%) in the stepped monotherapy cohort, 103 participants (20.2%) in the 2-pill cohort, and 35 participants (13.8%) in the 3-pill cohort were still taking their baseline regimen (eTable 11 in Supplement 3). Numbers of prescribed pills per group and time point were highest in those receiving the 3-pill strategy (eTable 12 in Supplement 3).

### Adherence

The proportions of participants with 90% or greater adherence at 12 weeks were 77% (342 of 446 participants with adherence data), 83% (375 of 454 participants), and 79% (173 of 219 participants) in the stepped monotherapy, 2-pill, and 3-pill cohorts, with aORs of 1.45 (95% CI, 1.04-2.02; *P* = .03) and 1.12 (95% CI, 0.75-1.66; *P* = .59), respectively, vs those in the stepped monotherapy cohort (Table 2 and eTable 13 in Supplement 3). The most frequent reason indicated for nonadherence was forgetting to take the medication. Determining adherence by pill count for each medication did not show differences (eTable 14 in Supplement 3).

Over 24 weeks, 138 participants (27%) in the stepped monotherapy cohort, 111 participants (22%) in the 2-pill cohort, and 61 participants (24%) in the 3-pill cohort were lost to follow-up or stopped treatment with evidence of slightly lower risk among those in the 2-pill cohort (Table 2).^28^

### Hypertension-Mediated Organ Damage

No difference in hypertension-mediated organ damage between strategies was noted (Table 2).^28^ At enrollment, left ventricular hypertrophy was documented in 4% to 6% of participants by focused echocardiography and 56% by electrocardiography (eTable 15 in Supplement 3). By week 24, 5 participants developed new left ventricular hypertrophy, while 30 (7%), 29 (7%), and 18 (9%) receiving the stepped monotherapy, 2-pill, and 3-pill strategies had diminished ventricular hypertrophy. Kidney impairment occurred newly at week 24 in 8% of participants receiving all 3 treatment strategies, worsened in 1% to 2%, and resolved in 16% to 20% (eTable 16 in Supplement 3). There were 13 major cardiovascular events and 7 deaths due to cardiovascular events (Table 2).^28^

### Adverse Events

There were 303, 303, and 126 adverse events in 179 participants (35%) in the stepped monotherapy cohort, 174 participants (34%) in the 2-pill cohort, and 71 participants (28%) in the 3-pill cohort (Table 2).^28^ In post hoc analyses, the number of participants with at least 1 adverse event was significantly lower in those receiving the 3-pill vs monotherapy strategy (71 of 253 [28%] vs 179 of 505 [35%]; *P* = .03) without a difference between those receiving the 2-pill vs monotherapy strategy (174 of 510 [34%] vs 179 of 505 [35%]; *P* = .58). The higher number of adverse events among participants in the stepped monotherapy and 2-pill cohorts was more often probably or definitely related to study drug, being mostly lower limb edema while taking amlodipine. During follow-up, 18 female participants (2%) became pregnant, resulting in the delivery of 7 healthy babies, 7 spontaneous abortions, 1 postpartum death, and 3 losses to follow-up.

### Post Hoc Analyses

In post hoc analyses, participants younger than 65 years receiving the 2- and 3-pill strategies were more likely to attain target blood pressure compared with those taking the stepped monotherapy strategy (aOR, 1.50; 95% CI, 1.10-2.06) and 1.55 (95% CI, 1.06-2.29), with no intervention benefits among those 65 years and older (eTable 17 and eFigure 3A-B in Supplement 3). Using the essential blood pressure target (≤140/90 mm Hg),^13^ 75% of participants in the stepped monotherapy cohort, 76% in the 2-pill cohort, and 79% in the 3-pill cohort met the target at 12 weeks, with no difference between the strategies (eTable 18 in Supplement 3).

## Discussion

In this large-scale, multisite, randomized clinical trial on different antihypertensive treatment strategies, starting low-dose CCB/ARB antihypertensive treatment was noninferior compared with full-dosed CCB monotherapy with stepped escalation for control of blood pressure in individuals with uncomplicated hypertension in Africa. Neither starting 2 pills (CCB/ARB) nor starting 3 pills (CCB/ARB/hydrochlorothiazide) with escalations as needed was superior to starting stepped monotherapy in terms of target blood pressure attainment at 12 weeks. In post hoc analyses, among participants younger than 65 years, those receiving the 2- and 3-pill strategies were more likely to achieve the blood pressure target. Adverse events were mild, more common in the stepped monotherapy strategy, and mostly attributable to amlodipine. More treatment adaptations were needed in the 3-pill strategy. Left ventricular hypertrophy and kidney impairment rarely occurred or worsened but were resolving in nearly a fifth of participants in all treatment cohorts.

The mean blood pressure reduction of nearly 30 mm Hg systolic in all 3 treatment strategies with a mean systolic blood pressure of 125 mm Hg within 8 to 12 weeks in all 3 groups was remarkable and more pronounced compared with other trials.^16,30^ This might be due to inclusion of amlodipine in all strategies, with some regression to the mean. A meta-analysis including studies from Asia, Australia, the US, and Europe showed better blood pressure control with low-dose triple combination compared with full-dose monotherapy.^31^ However, Black people were underrepresented, and starting drug dosages were higher without up titration. A recent trial from Nigeria randomizing 300 participants to a low-dose triple-combination pill (telmisartan, amlodipine, and indapamide) vs amlodipine, 5 mg, showed improved blood pressure control in the triple-combination group.^32^

Our study showed a nonsignificant trend of improved blood pressure response in those receiving the triple therapy compared with monotherapy strategy. Reasons for this might be the higher than expected treatment response in the stepped monotherapy strategy (51%), a high amlodipine starting dose,^33^ the use of amlodipine in all strategies, the up titration of drugs, and the choice of losartan, which might not be the most potent ARB.^34^ This might have led to a smaller difference in blood pressure control than initially assumed.

We based blood pressure targets on the ESC/ESH 2018 guidelines recommending less than or equal to 130/80 mm Hg for people younger than 65 years.^12,35^ A recent Cochrane review did not show reduction in mortality, cardiovascular events, or adverse effects when treating to a target of 135/85 mm Hg or less compared with 140/90 to 160/100 mm Hg or less.^36^ Therefore, the majority of guidelines worldwide recommend a target of 140/90 mm Hg.^37^ In contrast, the updated guidelines of the ESC 2023^38^ and of the ESH 2024^39^ continue to recommend a systolic blood pressure target of 120 to 130 mm Hg regardless of age and a diastolic target of 70 to 80 mm Hg, the latter with a class IIb C recommendation. In our study, not reaching a diastolic blood pressure of less than or equal to 80 mm Hg in those younger than 65 years was the most common reason not to reach the target. Using the target of less than or equal to 140/90 mm Hg regardless of age^13^ in a post hoc analysis led to higher blood pressure control rates similarly across the three treatment strategies.

Mild adverse events were more common with the stepped monotherapy and 2-pill strategies compared with the 3-pill strategy, mostly attributable to lower limb edema when taking higher-dose amlodipine.^40^ Serious adverse events were rare and similar in all strategies, in line with other studies.^16,31^ Despite the higher pill burden, the 2-pill strategy showed slightly better adherence compared with stepped monotherapy. On the other hand, in the 3-pill strategy, up-titrating steps were needed more frequently than in the stepped monotherapy strategy, indicating that the drug dosages chosen might have been too low.

We found no differences between the 3 strategies for left ventricular hypertrophy or kidney impairment, possibly due to the short follow-up time of 24 weeks. High rates of left ventricular hypertrophy were present in all strategies at baseline, confirming recent observational studies on hypertension-mediated heart failure in African individuals.^41^

### Limitations

This study has limitations. First, the primary outcome was not assessed in 12% of participants mainly due to loss to follow-up. However, the sample size calculation allowed for 15% loss to follow-up, and we used multiple imputation to account for missingness. Second, the trial was not blinded. Third, we did not use single-pill combinations as these were not available at the time in both rural settings. Thus, drug selection for the trial was based on availability and affordability in low- and middle-income countries. This might have affected adherence. However, adherence was high, with 77% to 83% of participants reaching 90% or greater correct pill intake in all groups. Fourth, although we powered for a difference of 15% target attainment, the study might have been underpowered for smaller differences, which could be clinically relevant. Fifth, the study was conducted during the COVID-19 pandemic with several interruptions in recruitment.

## Conclusions

In conclusion, results of this randomized clinical trial show that an antihypertensive strategy starting a low-dose 2-pill treatment was noninferior to starting a CCB monotherapy with stepped escalation to add hydrochlorothiazide if needed. We did not find improved blood pressure control when starting low-dose 2-pill (CCB/ARB) and 3-pill (CCB/ARB/hydrochlorothiazide) combinations compared with stepped CCB monotherapy. However, wide CIs preclude the ability to rule out potentially clinically important effects of the additional pill strategies for hypertension control. Initial monotherapy with full-dose amlodipine led to a rapid blood pressure reduction at the cost of increased risk of adverse events, whereas the combination strategies might improve blood pressure control in those younger than 65 years targeting a lower blood pressure. Future trials should examine higher starting triple-combination drug dosages, different ARBs, and evaluate single- vs multipill regimens.

## Notes

Supplement 1.
Trial Protocol.

Supplement 2.
Statistical Analysis Plan.

Supplement 3. **eAppendix.** Statistical Analysis **eTable 1.** Treatment Schedules **eTable 2.** Estimand Framework: Primary Outcome for Superiority Comparisons, Resembling Intention-to-Treat Approach **eTable 3.** Estimand Framework: Primary Outcome for Noninferiority Comparison, Resembling Per Protocol Approach **eTable 4.** Definition of Outcome of Surrogate Markers for Hypertension-Mediated Organ Damage **eTable 5.** Definition of Major Cardiovascular Events **eTable 6.** Primary Outcome: Additional Results **eTable 7.** Primary Outcome: Effect Modification **eTable 8.** Blood Pressure and Target Attainment Over Time **eTable 9.** Blood Pressure Changes at 4, 8, 12, and 24 Weeks **eTable 10.** Target Blood Pressure at 4, 8, and 24 Weeks (ITT Approach) **eTable 11.** Treatment Escalations Over Time **eTable 12.** Treatment Prescribed by Visit Week **eTable 13.** Adherence to Study Medication **eTable 14.** Adherence to Medication by Pill Count **eTable 15.** Left Ventricular Hypertrophy **eTable 16.** Renal Impairment **eTable 17.** Post Hoc Analysis of Effect Modification by Age **eTable 18.** Essential Target Blood Pressure Attainment **eFigure 1.** Time to Achieve Target Blood Pressure **eFigure 2.** Treatment Prescribed by Visit Week **eFigure 3.** Blood Pressure Target Attainment

Supplement 4.
Data Sharing Statement.
