# Supplementary material for: Treatment Strategies to Control Blood Pressure in People With Hypertension in Tanzania and Lesotho: A Randomized Clinical Trial
Source: JAMA Cardiol. 2025 Jan 29;10(4):321–33. doi: 10.1001/jamacardio.2024.5124 (PMC11780507; doi:10.1001/jamacardio.2024.5124)
Supplement: Supplement 1. — Trial Protocol. [file jamacardiol-e245124-s001.pdf]

Identifying most effective Treatment Strategies to control Arterial Hypertension in sub-Saharan Africa- A Randomized Controlled Trial

coArtHA

Study Type:	Clinical trial evaluating a drug treatment strategy
Risk Categorisation:	Risk category A
Study Registration:	Clinical Trials Gov (NCT04129840)
Sponsor-Investigator:	Prof Dr. Maja Weisser Rohacek<br>Division of Infectious Diseases & Hospital Epidemiology<br>University Hospital Basel, Petersgraben 4, 4031 Basel, CH<br><br>Department of Medicine, Swiss Tropical and Public Health Institute, Socinstrasse 57, 4051 Basel, CH<br><br>Ifakara Health Institute, Mlabani Passage, PO Box 53, Ifakara, TZ<br><br>Email: m.weisser@unibas.ch; mweisser@ihi.or.tz<br>Tel +41 (0)61 328 67 42 / +255 (0)62 102 95 69
Principle Investigator:	Jacqueline Nkouabi, Ifakara Health Institute<br>jnkouabi@ihi.or.tz
Investigated Intervention:	Open-label randomized trial on the best drug combination for arterial hypertension in sub-Saharan Africa
Protocol ID	coArtHA
Version and Date:	Version 1.1 (12.10.2022)

CONFIDENTIALITY STATEMENT

The information contained in this document is confidential and the property of the sponsor-investigator. The information may not - in full or in part - be transmitted, reproduced, published, or disclosed to others than the applicable Competent Ethics Committee(s) and Regulatory Authority(ies) without prior written authorisation from the sponsor except to the extent necessary to obtain informed consent from those who will participate in the study.

PROTOCOL SIGNATURE FORM

Study Title	Identifying most effective Treatment Strategies to control Arterial Hypertension in sub-Saharan Africa - A Randomized Controlled Trial
Study ID	coArtHA

The Sponsor-Investigator has approved the current amendment (protocol version 0.7 (22.10.2020) and confirm hereby to conduct the study according to the protocol, current version of the World Medical Association Declaration of Helsinki, and ICH-GCP guidelines as well as the local legally applicable requirements.

Sponsor-Investigator: Prof Dr. Maja Weisser
Division for Infectious Diseases, University hospital Basel, Switzerland
Ifakara Health Institute, Mlabani Passage, PO BOX 53, Ifakara, Morogoro, Tanzania

Date: 17.10.2022 Signature:

Principal Investigator and site PI Ifakara: Dr. Jacqueline Nkouabi
Ifakara Health Institute, Mlabani Passage, PO BOX 53, Ifakara, Morogoro, Tanzania

Date: 17.10.2022 Signature:

Site Principal Investigator at the Lesotho site: Dr. Ravi Gupta
SolidarMed Lesotho, Mokhotlong Government Hospital, Mokhotlong, Lesotho
Headquarter: SolidarMed, Partnership for health; House 3A Happy Villa, Maseru P.O.Box 0254, Maseru, Lesotho

Date: 17.10.2022 Signature:

TABLE OF CONTENTS

GLOSSARY OF ABBREVIATIONS **5**

1. STUDY SYNOPSIS **6**

2. BACKGROUND INFORMATION AND RATIONALE **11**

2.1. Current state of research in the field *11*
2.1.1 The burden of high blood pressure in sub-Saharan Africa *11*
2.1.2 The double burden of HIV and high blood pressure *11*
2.1.3 Pharmacologic blood pressure therapy in sub-Saharan Africa – an understudied topic *12*
2.1.4 Randomized trials on pharmacologic therapy of hypertension conducted in Africa *12*
2.1.5 Background Information on the 2 study sites *13*

3. STUDY OBJECTIVES AND DESIGN **14**

3.1. HYPOTHESIS AND OBJECTIVES 14
3.1.1. Overall objective *14*
3.1.2. Specific Objectives *14*
Primary Objective *14*
Secondary Objectives *14*
3.1.3. Study Arms and Rationale *15*
3.2. PRIMARY AND SECONDARY ENDPOINTS 18
3.2.1. Primary Endpoint *18*
3.2.2. Secondary Endpoints *18*
3.2.4. Effect modification *19*
3.3. STUDY DESIGN 19
3.3.1. General study design and justification of design *19*
3.3.2. Methods of minimizing bias *19*
Randomization *19*
Blinding *19*
3.4. STUDY INTERVENTION 19
3.4.1. Overview of the 3 treatment strategies *19*
3.4.2. Intervention and Control Arms *20*

4. STUDY POPULATION AND STUDY PROCEDURES **21**

4.1. THE RESEARCH SITES 21
4.1.1. Inclusion Criteria *22*
4.1.2. Exclusion Criteria *22*
4.2. RECRUITMENT STRATEGY, SCREENING AND INFORMED CONSENT PROCEDURE 22
Assignment to Study Arm *23*
4.3. STUDY PROCEDURES 23
4.3.1. Study Schedule *23*
4.3.2. Procedures at each visit *25*
Overview over study procedures *25*
Pre-Screening (D0) *25*
Screening (D0) *25*
Enrolment (D0) *26*
Follow-up visits (weeks 4, 8, 12) *26*
Follow-up visit (week 24) *27*
4.3.3. Measurements and definitions of blood pressure *27*
4.4. WITHDRAWAL/DISCONTINUATION 28

5. STATISTICS AND METHODOLOGY **28**

5.1. STATISTICAL PLAN AND SAMPLE SIZE CALCULATION 29
5.1.1. Determination of Sample Size *29*
5.1.2. Planned analyses *29*
Primary Analyses *29*

Interim Analyses 30
Cost Effectiveness 30
5.2. Handling on missing data and drop-outs 31

6. REGULATORY ASPECTS AND SAFETY 31

6.1. Ethical Conduct of the Study 31
6.2. (Serious) Adverse Events 31
Reporting of SAEs 32
6.3. Protocol amendments 32
6.4. Early termination of the Study 32
6.5. Insurance 33

7. FURTHER ASPECTS 33

7.1 Overall ethical considerations 33
7.2 Risk-benefit assessment 33

8. QUALITY CONTROL AND DATA PROTECTION 34

8.1. Quality Measures 34
8.2. Data Recording and Source Data 34
8.2.1. Case Report Forms 34
8.2.2. Data collection 35
8.2.3. Data management 35
8.2.4. Data Security and Storage 35
8.2.5. Specification of source documents 36
8.3. Confidentiality and coding 36
8.3.1. Participant privacy and confidentiality 36
8.4 Retention and destruction of study biological material 36

9. MONITORING AND REGISTRATION 37

9.1. Study Registration 37
9.2. Study monitoring 37
9.2.1. Monitoring Institution 37
9.2.2. Data Safety Monitoring 37

10. FUNDING/PUBLICATION/DECLARATION OF INTEREST 37

Schedule and milestones (2 years) 37
Declaration of interest 38

11. STUDY ADMINISTRATIVE INFORMATION 38

11.1. Investigators and Study Team 38

12. BIBLIOGRAPHY 39

Glossary of Abbreviations

ACE-I	Angiotensin converting enzyme inhibitors
AE	Adverse Event
ASR/DSUR	Annual Safety Repot / Development Safety Report
ARB	Angiotensin II Receptor Blocker
ART	Antiretroviral treatment
BP	Blood Pressure
BASEC	Business Administration System for Ethical Committees
CCB	Calcium Channel Blocker
CDCI	Chronic Diseases Clinic Ifakara
CK-EPI	Chronic Kidney Disease Epidemiology Formula
ClinO	Ordinance on Clinical Trials in Human Research (in German: KlinV, in French: OClin, in Italian: OSRUm)
CTCAE	Common Terminology Criteria for Adverse Events
CRF	Case Report Form
DBP	Diastolic Blood Pressure
eCRF	electronic Case Report Form
FADP	Federal Act on Data Protection (in German: DSG, in French: LPD, in Italian: LPD)
GCP	Good Clinical Practice
HCT	Hydrochlorothiazide
HRA	Human Research Act (in German: HFG, in French: LRH, in Italian: LRUm)
ICH	International Conference on Harmonisation
IHI	Ifakara Health Institute
MKGH	Mokhotlong Government Hospital
RAS-I	Renin-angiotensin system-inhibitor
SAE	Serious Adverse Event
SBP	Systolic Blood Pressure
SFRH	St. Francis Referral Hospital
SwissTPH	Swiss Tropical and Public Health Institute
USB	University Hospital Basel
TZD	Thiazide diuretic
WHO	World Health Organization

1. Study Synopsis

Sponsor-Investigator	Maja Weisser Rohacek
Study Title:	Identifying most effective Treatment Strategies to control Arterial Hypertension in sub-Saharan Africa - A Randomized Controlled Trial
Short Title / Study ID:	coArtHA
Protocol Version and Date:	Version V1.1; 12.10.2022
Study registration:	NCT04129840
Study category and Rationale	This study is classified risk category A because<br>1) All drugs are established, well studied and licensed<br>2) The intervention is a treatment algorithm and not an individual drug
Background and Rationale:	The rollout of antiretroviral treatment (ART) has led to a dramatic reduction in HIV/AIDS-associated deaths. With increased life-expectancy, HIV-positive individuals are at increased risk of cardiovascular diseases. The double burden of HIV and arterial hypertension - the most prevalent risk factor for cardiovascular disease in sub-Saharan Africa - is a major challenge especially in rural areas due to low awareness in communities, lack of screening programs, high cost of antihypertensive medication and the general lack of evidence regarding optimal antihypertensive treatment for Africans living in sub-Saharan Africa. The World Health Organization (WHO) recommends starting treatment of arterial hypertension with a thiazide diuretic (TZD) or a calcium channel blocker (CCB) and a dual combination, if the target blood pressure is not achieved. Newer antihypertensive drugs and treatment strategies are available but have not been compared to the WHO approach in Africa.<br>With the proposed randomized controlled 2-center trial, we aim at closing this evidence gap, comparing two combination treatment strategies with the WHO standard aiming at rapid control of arterial hypertension: a dual combination of a CCB and an angiotensin II receptor blocker (ARB) starting at half dose), and a triple combination of CCB, TZD and ARB starting at a quarter dose with optional dose titration after 4 and 8 weeks, if target blood pressure is not reached.
Risk / Benefit Assessment	Participants of this trial will benefit from blood-pressure treatment according to the current standard of care or benefit from newer drugs, which are included in international guidelines but have not been well studied. The risk of side-effects due to drugs is small, as all drugs are already licensed and available in the countries of both study sites.
Objectives:	The main objective is to compare efficacy of 3 antihypertensive treatment strategies using locally available antihypertensive drugs in HIV-positive and HIV-negative patients in an open-label, two-country, controlled randomized trial: the current WHO-recommended treatment and two alternatives, potentially more efficient treatments. The trial will focus on patients with uncomplicated arterial hypertension. It will be conducted in partnering hospitals in Ifakara, Tanzania and Mokhotlong, Lesotho and compare the effectiveness and safety of the three strategies. For one sub-study: To compare blood pressure measurement techniques (repeated clinic versus ambulatory blood pressure measurement.
Endpoints	<u>Primary endpoint</u>

	• The proportion of patients reaching a target blood pressure (clinic BP) of ≤130/80mmHg among patients <65years of age and ≤140/90mmHg among patients ≥65years of age with uncomplicated arterial hypertension in Tanzania and Lesotho at 12 weeks.<br><u>Secondary endpoints</u><br>• The proportion of patients reaching a target blood pressure of ≤130/80mmHg among patients <65years of age and ≤140/90mmHg among patients ≥65years of age at 4, 8, and 24 weeks<br>• The reduction in blood pressure at 4, 8, 12 and 24 weeks<br>• The proportion of patients with (and number of) treatment adaptations of primary treatment (dose increases and/or drug additions) within 12 weeks<br>• Time until target blood pressure of ≤130/80mmHg in patients <65years of age and ≤140/90mmHg in patients ≥65years of age is reached<br>• The proportion of patients with changes in surrogate markers for end-organ damage: kidney impairment measured by albumin/creatinine ratio in urine, serum creatinine, echocardiographic and electrocardiographic signs of hypertensive heart disease, signs of retinopathy) within 24 weeks (resolving, newly occurring or worsening)<br>• The proportion of patients with clinical endpoints (mortality, major cardiovascular events such as stroke, myocardial infarction, heart failure, end-stage kidney disease) in the 3 arms within 24 weeks<br>• The proportion of patients lost to follow-up or stopped treatment within 24 weeks<br>• The proportion of patients with grade 3/4 adverse events within 24 weeks<br>• The proportion of patients with serious adverse events within 24 weeks<br>• Adherence and reasons for non-adherence<br>• Cost-effectiveness of the treatment strategy to lower blood pressure<br>• Effect modification of the primary endpoint by site and HIV status
Study design:	Open-label, two-country, controlled randomized trial of 3 different treatment strategies. The trial is designed for a superiority comparison between the triple combination arm (quarter dose) versus control, and non-inferiority for the dual combination arm (half dose) versus control (see below).
Statistical Considerations:	Statistical analysis will follow intention-to-treat (ITT) principles. Baseline characteristics will be described by arm with summary statistics; no formal testing between arms will be performed. Outcomes will be described by arm using summary statistics. The primary outcome, and binary secondary outcomes, will be assessed using logistic regression models, reporting odds ratios with 95% confidence intervals (CI). Continuous secondary outcomes will be assessed using linear regression models, reporting adjusted mean differences between arms with 95% CIs. Time to event outcomes will be assessed using appropriate methods such as Kaplan-Meier estimation and Cox proportional hazards models. All models will be adjusted for baseline blood pressure and the stratification factors of site, HIV status and age. Primary analyses will be complete case. We will compare each of the intervention arms versus control. Primary analyses for the non-inferiority comparison will be performed on both the ITT and per protocol sets. If the

	dual combination is found to be non-inferior to the control, then we will assess for superiority.
Inclusion / Exclusion criteria:	<u>Inclusion:</u><br>• HIV-positive and negative patients ≥18 years of age of African descent and black ethnicity with a documented uncomplicated, untreated arterial hypertension (blood pressure ≥140/90mmHg) diagnosed at one of the 2 study sites<br><u>Exclusion</u>:<br>• Current hospitalization for any reason<br>• Not of African descent<br>• Refusal of an HIV-test or indeterminate HIV test result<br>• History of cardiovascular event in the last month (anginal pain, stroke, myocardial infarction or diagnosis by a doctor)<br>• Symptomatic arterial hypertension (blood pressure ≥180/110mmHg plus acute headache or chest pain) or acute cardiovascular event<br>• acute disease, e.g.:<br>◦ fever >37.5°C or other signs of acute concomitant infection<br>◦ Dyspnea/respiratory distress<br>◦ Acute pain<br>• Clinical signs of hypertension-mediated organ damage<br>◦ heart failure (bilateral pitting edema, bilateral crackles or pleural effusion, distended jugular veins)<br>◦ ischemic heart disease (anginal pain on exertion)<br>◦ signs of current ischemic/hemorrhagic stroke (hemiparesis, loss of consciousness)<br>• Pregnancy (test required for females 18-45y of age)<br>• Non-consenting or inability to come for follow-up visits<br>• creatinine clearance ≤30ml/min by Chronic Kidney Disease Epidemiology Formula (CK-EPI) estimation and measurement with a point-of care creatinine from capillary blood
Number of Participants with Rationale:	The response rate in the control arm is assumed to be 40%. For the superiority comparison between the triple combination and control arms, we assume an improvement in the triple combination arm of 15% (two-sided alpha of 0.05). For the non-inferiority comparison between the dual combination and control arms, we assume a non-inferiority margin of 10% (one-sided alpha of 0.025). A sample size of 431 patients in each of the control and dual combination arms, and 216 patients in the triple combination arm, yields a power of 85% for the non-inferiority comparison and a power of 95% for the superiority comparison. The overall sample size is therefore 1078 patients, with the randomization ratio of 2:1:2 for the dual combination, triple combination and control arms, respectively. Assuming 15% of participants will become lost-to-follow-up (60) brings the total required sample size to 1268 individuals. Each site will enrol at least 500 participants, after which enrolment will be competitive.
Study Intervention:	A) **Intervention 1:** dual combination of half-dose CCB and ARB, dosage increases at 4 and 8 weeks if target blood pressure* is not reached at the respective time point<br>B) **Intervention 2:** triple combination of quarter-dose of CCB, TZD and ARB with dosage increases of all drugs at 4 and 8 weeks, if target blood pressure* is not reached at the respective time point

	*target blood pressure (clinic BP):≤130/80mmHg for patients aged <65years and ≤140/90mmHg for patients aged ≥65years
Control Intervention:	C) **Standard of care:** start normal dose CCB, add TZD after 4weeks and increase of TZD dosage after 8, weeks if target blood pressure* is not reached at the respective time point
Study procedures:	Patients will be consecutively enrolled and randomized in the ratio 2:1:2 to the dual combination, triple combination or control arms, respectively, using a pre-generated randomization list stratified by site, HIV status and age with randomly varying block sizes. All patients will provide written informed consent. Enrolled patients will receive a clinical evaluation including an interview and physical examination, and a standardized assessment of blood pressure at every visit. Additionally, surrogate markers of hypertension-induced end organ damage will be assessed at baseline and at the end of the study at 24 weeks.
Study Duration and Schedule:	The initially planned study duration (recruitment 12 months, follow-up 6 months. Due to a delayed start and temporary stop of recruitment during the COVID-19 pandemic with reduced community activities and lower numbers of patients seen in the hospitals, the study duration needs extension (recruitment 26 months, follow-up 6 months, total 32 months)<br>05.03.2020 First-Participant recruited<br>30.10.2022 Last-Participant-Out (planned)
Investigator(s):	Maja Weisser Rohacek (Sponsor-Investigator)<br>*Staff Physician Div of Infectious Diseases, University Hospital Basel, CH, Clinical Research Coordinator, Chronic Diseases Clinic of Ifakara, Ifakara Health Institute, Ifakara, TZ; m.weisser@unibas.ch*<br><br>Jacqueline Nkouabi *(Principal Investigator)*<br>*Clinician and Researcher Chronic Diseases Clinic, Ifakara Health Institute, Ifakara, TZ; jnkouabi@ihi.or.tz*<br><br>***Herry Mapesi*** *(Principal Investigator)*<br>*Clinician and Researcher Chronic Diseases Clinic, Ifakara Health Institute, Ifakara, TZ; hmapesi@ihi.or.tz*<br><br>***Thilo Burkard*** *(Co-Investigator)*<br>*Head of Hypertension Centre, Staff physician, Medical Outpatient Department and Department of Cardiology, University Hospital Basel, CH; Thilo.burkard@usb.ch*<br><br>***Niklaus Labhardt*** *(Co-Investigator)*<br>*Project leader, Swiss Tropical & Public Health Institute, University Basel,CH; n.labhardt@unibas.ch*<br><br>***Martin Rohacek*** *(Co-Investigator)*<br>*Head of Emergency Dpt, St. Francis Referral Hospital; Research Scientist, Ifakara Health Institute, Ifakara, TZ, m.rohacek@ihi.or.tz*<br><br>***Ravi Shankar Gupta*** *(Co-Investigator (site Investigator Lesotho))*<br>*Project Manager, Health First Lesotho SolidarMed; LT,R.Gupta@solidarmed.ch*<br><br>***Herieth Wilson*** *(Site investigator, Ifakara)*

	Research officer (clinician), Chronic Diseases Clinic Ifakara, Ifakara Health Institute, Ifakara, TZ; hwilson@ihi.or.tz<br><br>***Elisabeth Senkoro*** *(Site investigator, Ifakara)*<br>*Research officer (clinician), Chronic Diseases Clinic Ifakara, Ifakara Health Institute, Ifakara, TZ; esenkoro@ihi.or.tz*<br><br>***Blaise Dilumbu Lukau*** *(Site investigator, Mokhotlong)*<br>*Mokhotlong Government Hospital, SolidarMed Lesotho, lukaublaise@yahoo.fr*<br><br>***Madavida Mphunyane (Co-investigator****; Lesotho)*<br>*MOH Lesotho NCD Program Manager. mphunyanem@gmail.com*<br><br>***Alain Amstutz*** *(Co-Investigator (co-site PI Lesotho))*<br>*PhD student Clinical Research, Swiss TPH, University Hospital Basel, University of Basel Alain.amstutz@unibas.ch*<br><br>***Fiona Vanobberghen*** *(Project Statistician)*<br>*Statistician at the Swiss Tropical and Public Health Institute, University of Basel, CH; Fiona.vanobberghen@swisstph.ch*<br><br>***Moniek Bresser*** *(Project Data Management))*<br>*Swiss Tropical and Public Health Institute, University of Basel, CH; Moniek bresser@swisstph.ch*<br><br>***Tracy Glass*** *(supervising statistician)*<br>*Lead Statistician of KIULARCO Swiss TPH, University of Basel, CH; tracy.glass@swisstph.ch*<br><br>***Günther Fink***<br>*Co-Investigator (cost-effectiveness analysis)*<br>*Head of Household Economics and Health Systems, Swiss Tropical and Public Health Institute, University of Basel, CH; Guenther.fink@swisstph.ch*<br><br>***Mark Lambrinis***<br>*Co-Investigator (cost-effectiveness analysis)*<br>*Research Scientist Household Economics and Health Systems, Swiss Tropical and Public Health Institute, University of Basel, CH; mark.lambrinis@swisstph.ch*
Study Centre(s):	1) St. Francis Referral Hospital/IfakaraHealthInstituteatIfakara, Morogoro, Tanzania<br>2) Mokhotlong Government Hospital, SolidarMed Lesotho, Mokhotlong, Lesotho
Data privacy	All data will be handled confidentially. Anonymity of the participants will be guaranteed from data entry to analysis and publication. Individual subject medical information is further ensured by utilizing subject identification code numbers.
Ethical consideration	This study will inform the best treatment option for arterial hypertension for people living in sub-Saharan Africa, where arterial hypertension is a relevant and prevalent health concern, being the major risk factor for cardiovascular endpoints. As previous studies have shown, mono-treatment of arterial hypertension among individuals of African descent is often insufficient –this trial aims at comparing different treatment

	strategies and analysing organ damage such as renal impairment, cardiac function and ocular manifestations as secondary endpoints.
GCP Statement:	This study will be conducted in compliance with the protocol, the current version of the Declaration of Helsinki, the ICH-GCP as well as all national legal and regulatory requirements.

2. Background Information and Rationale

2.1. Current state of research in the field

2.1.1 The burden of high blood pressure in sub-Saharan Africa

The global rollout of antiretroviral treatment (ART) programs has led to dramatic decreases in AIDS-related mortality and increases in life expectancy of people living with HIV, particularly in sub-Saharan Africa (1). The large reductions in AIDS-related mortality have been paralleled by an increase in cardiovascular morbidity and mortality in low-and middle-income countries (2, 3).

Black ethnicity is a risk factor for elevated blood pressure and associated cardiovascular events (4). In sub-Saharan Africa age-standardized mean systolic blood pressure is approximately 5-20mmHg higher compared to North America or Europe (2). Prevalence of elevated blood pressure ranges between 30-46% (5-11). While the causal determinants of this increased hypertension risk have not been fully determined yet, genetic factors, including epigenetic adaptation to climate (12, 13) and increased susceptibility to salt intake (14, 15) have been identified as factors potentially affecting the increased risk. Complications of arterial hypertension (stroke, kidney failure) have shown to be higher in Afro-Americans compared to white people, according to data coming mostly from comparisons within the USA (16).

A systematic review from 2007 found that less than 40% of hypertensive patients in sub-Saharan Africa have ever been diagnosed, less than 30% of those being diagnosed receiving medical treatment, and less than 20% of those being treated having good blood pressure control (8). On these grounds, the WHO released a Global Action Plan in 2013 to address arterial hypertension with the following goals to be reached by 2020: (1) relative reduction of the prevalence of raised BP by 25% by adequate treatment; (2) 30% relative reduction in salt/sodium intake and at least 50% of people receiving drug therapy and counselling for prevention of stroke and ischemic heart disease; (3) 80% availability of basic technologies and medicines, to treat non-communicable diseases (17).

2.1.2 The double burden of HIV and high blood pressure

Figure 1 displays estimates of the World Health Organization on prevalence of arterial hypertension and HIV: in sub-Saharan Africa both epidemics overlap, leading to a double burden of disease, which is expected to increase with higher live expectancy (18). Persons living with HIV have higher incidence rates of cardiovascular events as compared to HIV-negative individuals (19-22). Reasons might be HIV-associated immune activation, life style (e.g. use of tobacco) and treatment-related factors. Whether HIV and arterial hypertension are directly associated is still under debate (23-25). The effect of different antiretroviral treatment (ART) regimens on blood pressure (BP) is unclear. Within the 'Systemic Coronary Risk Evaluation (SCORE) system' antiretroviral treatment is considered a risk modifier increasing cardiovascular risk (26). However, studies did not find antiretroviral treatment to be a major risk factor for arterial hypertension (27-33), while interaction with antihypertensive treatments might affect antihypertensive treatment responses (34).

Figure 1 WHO prevalence maps on HIV and raised blood pressure

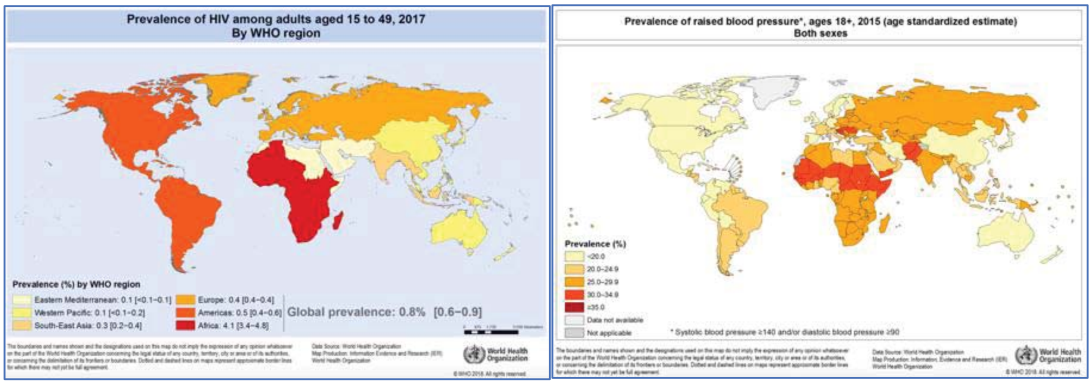

2.1.3 Pharmacologic blood pressure therapy in sub-Saharan Africa – an understudied topic

Life-style interventions (35) and pharmacotherapy (36) are the mainstay of recommended treatment for arterial hypertension. In high-income countries no clear superiority in blood pressure reduction has been shown for the different first-line drug classes, which are: Thiazide diuretic (TZD), long-active calcium channel blockers (CCB), renin-angiotensin system-inhibitor (RAS-I), including angiotensin-converting enzyme inhibitors (ACE-I) and angiotensin II receptor blockers (ARB))(37-44). A 2018 Cochrane analysis on first-line antihypertensive treatment found best evidence for lower reduction in morbidity and mortality with TZD, compared to CCB and RAS-I, although lower quality evidence found the three drug classes to be similarly effective (45). Also, most patients need a combination of at least two antihypertensive drugs to achieve BP control (46, 47) and guidelines nowadays recommend start of a combination pharmacologic treatment with at least two classes of hypertensive drugs for patients with a blood pressure ≥140/90mmHg (36). Choice of antihypertensive drug combination is further guided by comorbidities and side-effects, such as presence of kidney impairment and cardiac function (36).

Optimal treatment of hypertension across ethnically (and genetically) diverse groups has been identified as one of the major gaps in the literature (48). Studies conducted in the United States suggest that patients of African descent respond less to ACE-I and Betablockers than Asian or Caucasian patients (36, 49). The reduced efficacy of ACE-I among Afro-American patients compared to white ones could be due to a minor role of angiotensin-II dependency of blood pressure, whereas ACE-I at higher dose might work through additional mechanisms (50). For this reason, international guidelines recommend TZD or CCB in patients of African descent (4, 51-53). Since more than two thirds of patients need combination therapy to reach optimal treatment targets – which will be even more in case of newer and tighter targets – the question is less about the optimal first line drug class but rather the optimal drug combination and combination strategy to reach the target in an optimal time frame.

2.1.4 Randomized trials on pharmacologic therapy of hypertension conducted in Africa

Despite the very high burden of arterial hypertension in sub-Saharan Africa there is a paucity of data addressing optimal pharmacologic therapy in the region. WHO guidelines recommend a sequential treatment approach starting with a TZD, and adding a CCB in case of insufficient response (44, 52). There is, however, not a lot of evidence supporting this approach: Only five small and one recent bigger randomized trials conducted in sub-Saharan Africa compared effectiveness of different pharmacologic

regimens on blood pressure (54-58): A small study from South Africa from 1980 compared once daily atenolol 100mg to chlorthalidone 25mg alone or a combination of both drugs in 24 patients of Zulu origin. Only the combination achieved significant reduction in systolic blood pressure (54). A randomized controlled trial including 409 patients with diastolic blood pressure of 90mmHg or higher in Johannesburg found superiority of nifedipine compared to verapamil, hydrochlorothiazide (HCT) or enalapril (55). A study from Nigeria compared patients with start on HCT and CCB added, CCB start and HCT added and a combination of HCT/CCB from the beginning and found response rates (blood pressure of <140/90mmHg at 48 weeks) of 67%, 76% and 50%, respectively (59). A more recent study included patients with uncomplicated hypertension and ≤2 cardiovascular risk factors. Treatment with a single-pill regimen of daily bisoprolol/hydrochlorothiazide 5/6.25mg (B/H) was compared to amlodipine/valsartan 5/160 mg (A/V) (56, 57). After 6 months of treatment, blood pressure fell by 19.5/12.0mmHg in the B/H group versus 24.8/13.2mmHg in A/V group. The between-group differences were 5.2mmHg (p<0.0001) for systolic and 1.3mmHg (p=0.12) for diastolic pressure. Dosage had to be increased in 45% versus 37% of patients respectively (p=0.13), and methyldopa added in 33% versus 9% (p<0.0001). One trial studied the addition of low-dose spironolactone to antihypertensive treatment as per guidelines in patients with type 2 diabetes mellitus and treatment-resistant hypertension in Cameroon. Addition of spironolactone resulted in significant decrease in blood pressure (58).

Recently, a single-blind trial randomized 728 patients aged 30-79 years with an office blood pressure of 150-179mmHg (treatment-naïve) or 140-159mmHg (on monotherapy) in 10 centers in 6 African countries from 06-12/2017 into one of three antihypertensive treatment regimen: amlodipine 5mg plus hydrochlorothiazide 12.5 mg or amlodipine 5mg plus perindopril 4mg or perindopril 4mg plus hydrochlorothiazide 12mg and stratified according age (</>55years). Dosages were doubled after 2 months, unless side effects were present or the systolic pressure fell below 100mmHg. The primary outcome of change in ambulatory systolic blood pressure at 6 months was -18.1, -17.1 and -14.2, respectively in the 621 patients included in the final analysis (available ambulatory blood pressure measurement at study start and end)(60).

In summary, there is only moderate evidence which pharmacologic treatment approach might be best for achieving rapid and safe blood pressure control in patients with elevated blood pressure in sub-Saharan Africa. We plan to conduct an open-label two-country, superiority randomized controlled trial to address the best treatment algorithm in HIV-positive and HIV-negative patients with arterial hypertension in Tanzania and Lesotho. This trial aims to inform future guidelines, assessment of hypertension-mediated organ damage and cost-effectiveness of different strategies.

2.1.5 Background Information on the 2 study sites

Ifakara: Within a longstanding collaboration of the Swiss Tropical and Public Health Institute, the University Hospital Basel, the Ifakara Health Institute and the St. Francis Referral Hospital, an implementational project to build up and ensure HIV-services in rural Tanzania was started 2005. The 'Chronic Disesases Clinic Ifakara' (CDCI) at the St. Francis Referral Hospital in Ifakara hosts an HIV-positive patient cohort - the Kilombero and Ulanga Antiretroviral Cohort (KIULARCO) aiming at improving HIV care in rural settings through better understanding of patients' needs (61, 62). Among patients attending the clinic, we found a viral suppression rate of 91% (63) and a decrease in mortality over the years (61). At enrolment into care 12% of patients were hypertensive and a further 9.6% developed hypertension during follow-up. Age, higher BMI and renal impairment were associated with arterial hypertension (64). Among patients on ART for at least 2 months, period prevalence of arterial hypertension overall was even higher with 26%, and 43% among patients aged 50 years and older. Furthermore, we found a prevalence of renal impairment of 16% at enrolment and further 13% developed renal impairment during follow-up. Known factors such as hypertension, older age, and advanced HIV diseases were strongly associated with renal impairment (65).

The CDCI is experienced in management of clinical trials following Good Clinical Practice (GCP) standards within international collaborations. These trials include a study on Adjunctive Sertraline for the Treatment of HIV-Associated Cryptococcal Meningitis (ASTRO-CM, NCT01802385); Prospective Study of Lopinavir Based ART for HIV Infected childreN Globally (LIVING Study, NCT02346487; eFASH: Ultrasound in managing Tuberculosis: A randomized controlled two-center study; PACTR201712002829221).
Lesotho: Since 2014 the Swiss Tropical and Public Health Institute (SwissTPH) has a collaboration with SolidarMed, a Swiss health organization for Health in Africa including 23 public and missionary clinics in two districts in Northern Lesotho. N. Labhardt (project collaborator) heads the research group in Lesotho, which is experienced in conducting large multi-center trials. All group members are GCP trained and experienced in conducting randomized clinical trials (66, 67). The group's major focus has been on HIV. It has, however, also addressed non-communicable comorbidities among HIV patients (68, 69) and community-based surveys assessing prevalence of diabetes and dyslipidemia(70). A summary of the group's research activities is available under the following link: https://www.swisstph.ch/en/projects/hiv-care-research-in-lesotho/.

3. Study Objectives and Design

3.1. Hypothesis and Objectives

We hypothesize that the proportion of patients reaching the primary endpoint of blood pressure control will be higher among those receiving a triple combination regimen with CCB, TZD and ARB compared to those treated according to the current WHO standard of care (start of CCB, addition of TZD if needed). Additionally, we hypothesize that a dual treatment with CCB and ARB will be non-inferior to the standard of care.

3.1.1. Overall objective

We plan to conduct a randomized, two-center, open-label randomized controlled trial to compare the effectiveness of three different antihypertensive treatment strategies in HIV-positive and HIV-negative patients with uncomplicated arterial hypertension in rural Tanzania and Lesotho.

3.1.2. Specific Objectives

Primary Objective

- To compare the effectiveness of three antihypertensive treatment strategies for reaching a target blood pressure (clinic BP) of ≤130/80mmHg among patients <65years of age and ≤140/90mmHg among patients ≥65years of age with uncomplicated arterial hypertension in Tanzania and Lesotho within 12 weeks.

Secondary Objectives

- To compare the proportion of patients reaching a target blood pressure of ≤130/80mmHg among patients <65years of age and ≤140/90mmHg among patients ≥65years of age at 4, 8 and 24 weeks across the 3 arms
- To compare the reduction in blood pressure (change from beginning) after 4, 8, 12 and 24weeks across the 3 arms
- To compare proportion of (and number of) treatment adaptations (dose increases and/or drug additions) within12 weeks in the 3 arms
- To compare the time until target blood pressure of ≤130/80mmHg in patients <65years of age and ≤140/90mmHg in patients ≥65years of age is reached

- To compare the proportion of patients with changes in surrogate markers for end organ damage in the 3 treatment arms, namely signs of kidney impairment measured by albumin/creatinine in urine, serum creatinine, echocardiographic and electrocardiographic signs of hypertensive heart disease, signs of retinopathy) after 24 weeks (resolving, newly occurring or worsening)
- To compare the proportion of patients with clinical endpoints (mortality, major cardiovascular events such as stroke, myocardial infarction, heart failure, end-stage kidney disease) in the 3 armswithin24 weeks
- To compare the proportion of patients who are lost to follow up or stop taking drugs by24 weeks in the 3 arms
- To compare adherence measured by pillbox-return and self-report and reasons for non-adherence across the 3 arms
- To compare proportion of patients with at least one grade 3/4 adverse event across the 3 arms
- To compare proportion of patients with at least one serious adverse event across the 3 arms
- To compare cost-effectiveness of the three treatment algorithms
- To assess effect modification of the primary outcome by site and HIV status
- For a sub-study: To compare repeated clinic versus 24h ambulatory blood pressure measurement in 100 patients at the Ifakara site

3.1.3. Study Arms and Rationale

Standard of care (Arm C): Participants will be started on regular dose of CCB with a) addition of TZD at 4 weeks, if needed*, b) increase of dose of TZD after 8 weeks, if needed*.

i.e. if target blood pressure is not achieved at this time point (target blood pressure defined as clinic BP ≤130/80mmHg in patients <65years and ≤140/90mmHg in patients ≥65years). See Figure 3 for details including dosing.

***Rationale for Arm C**: This arm is Standard of care and follows the WHO guidelines as well as the National Guidelines of Tanzania and Lesotho, which recommend a calcium channel blocker or a thiazide diuretic as first line and the combination of both, if not sufficient(4, 36, 52, 71). Several studies indicate good treatment response to CCB and TZD in patients of African descent (4, 36, 52), which has been shown in small studies in sub-Saharan Africa(55). Recently published guidelines rate the evidence regarding treatment of hypertension in patients of African descent as shown in Table 1:*

Table 1. Guidelines for treatment of hypertension in patients of African descent.

<table>
<tr><th>ACC/AHA/AAPA Guidelines 2017 (36)</th><th>ESC/ESH Guidelines 2018 (48)</th></tr>
<tr><td>
10.1.1. Racial and Ethnic Differences in Treatment
<table>
<tr><th colspan="3">Recommendations for Race and Ethnicity
References that support recommendations are summarized in Online Data Supplement 51.</th></tr>
<tr><th>COR</th><th>LOE</th><th>Recommendations</th></tr>
<tr><td>I</td><td>B-R</td><td>1. In black adults with hypertension but without HF or CKD, including those with DM, initial antihypertensive treatment should include a thiazide-type diuretic or CCB.[S10.1.1-1–S10.1.1-4]</td></tr>
<tr><td>I</td><td>C-LD</td><td>2. Two or more antihypertensive medications are recommended to achieve a BP target of less than 130/80 mm Hg in most adults with hypertension, especially in black adults with hypertension.[S10.1.1-5–S10.1.1-7]</td></tr>
</table>
</td><td>
Hypertension in ethnic groups
<table>
<tr><th>Recommendations</th><th>Class[a]</th><th>Level[b]</th></tr>
<tr><td>It is recommended that a two-drug combination, usually as an SPC, is used as initial therapy for most black patients.[c]</td><td>I</td><td>C</td></tr>
<tr><td>In black patients, initial antihypertensive treatment should include a diuretic or a CCB, either in combination or with a RAS blocker.[d 316,469]</td><td>I</td><td>B</td></tr>
<tr><td>In other ethnic groups, BP-lowering treatment may be based on the core treatment algorithm (see Figure 4).</td><td>IIb</td><td>C</td></tr>
</table>
©ESC/ESH 2018
ACE = angiotensin-converting enzyme; ARB = angiotensin receptor blocker; BP = blood pressure; CCB = calcium channel blocker; RAS = renin–angiotensin system; SPC = single-pill combination.
[a]Class of recommendation.
[b]Level of evidence.
[c]Except in patients with low grade 1 hypertension or frail older patients, in whom initial treatment with a single drug may be more appropriate.
[d]Angioedema is more common with ACE inhibitors and thus ARBs may be preferred.
</td></tr>
</table>

Arm A=Intervention 1: Participants will be started on a dual therapy with half dose of CCB and an ARB. If needed*, a) the dose of the CCB will be increased at 4 weeks, and b) the dose of the ARB at 8 weeks, if blood pressure remains uncontrolled *.

i.e. if target blood pressure is not achieved at this time pint (target blood pressure defined as clinic BP ≤130/80mmHg in patients <65years and ≤140/90mmHg in patients ≥65years). See Figure 3 for details including dosing.

Rationale for Arm A:In hypertensive black patients of African descent monotherapy is recommended only for patients <10mmHg above target (4) and some studies show better response for combination therapy (72). During the last years European and American guidelines shifted towards an initial combination therapy strategy with 2 drugs as standard to achieve blood pressure control in 'most' patients with arterial hypertension including adults of African descent(36, 48). However, data on combination treatments in sub-Saharan Africa remain scarce, especially regarding optimal combinations (see Table 2).

Table 2. Data on combination hypertension treatments in sub-Saharan Africa.

Study	***Outcome measure***	***Thiaziddiuretic***	***Calcium Channel blocker***	***ACE-Inhibitor***
Studies in Americans of African descent (summarized)				
Materson, NEJM 1993*(73)*<br>*- Sub-analysis in US men of African descent*	*Patients reaching a DBP ≤90mmHg*	*64% (HCT)*	*40-58% (Dil)*	*20-32% (Cap)*
Brewster, Annals of Internal Medicine 2004 (53)<br>*- Systematic review on hypertension in Americans of African descent*	*BP reduction*	*Δ 9.8-14.8/5.5-7.28mmHg*	*Δ 7.9-12.8/3.7-10.1mmHg*	*Δ 3.9-9.8/3.4-3.9mmHg*
Studies from sub-Saharan Africa				
Seedat YK, Br Med J 1980*(54)*<br>*- RCT in South Africa (N=24)*<br>*- Grade I and II arterial hypertension*	*BP reduction*	*CTD 25mg vs ATL 100mg vs placebo vs combination (poor data quality)*<br>*→No effect of ATL, minor effect of CTD, combination superior*		
Sareli, Arch Intern Med 2001*(55)*<br>*- RCT in South Africa (N=409)*	*Decrease of SBP/DBP*<br>*Need of combination*	*Δ12/8mmHg (HCT)*<br>*56%*	*Δ22/14mmHg (Nif)*<br>*Δ17/11mmHg (Ver)*<br>*29% (Nif), 22% (Ver)*	*Δ5/3mmHg (Ena)*<br><br>*76%*
Iyalomhe, ClinExpHypert 2013 *(59)*<br>*- RCT in Nigeria (N=90)*	*BP <140/90mmHg at 6 weeks*	*0%*	*37%*	*NA*
		Best response step up from CCB + added HCT compared to HCT + added CCB and HCT+CCB from start (76% vs 67% vs 50%, respectively)		
M'Buyamba-Kabangu; J. Hum Hypertens 2013 (56)<br>*- RCT in the Democratic Republic of Congo (N=183)*	*Decreaseof SBP/DBP*	***amlodipine/valsartan***<br>*Δ 24.8/13.2mmHg*	***bisoprolol/hydrochlorothiazide***<br>*Δ 19.5/12.0mmHg*	
Djoumessi; BMC Res Notes 2016 *(58)*<br>*- RCT in Cameroon (N=17)*<br>*- patients with resistant arterial hypertension and diabetes mellitus*	*Decreaseof SBP/DBP*	***Spironolacton 25mg***<br>*Δ33/14mmHg*	***Alternative treatment***<br>*Δ14/5mmHg*	
Ojii; NEJM 2019(60)<br>*- RCT in 6 African countries*	*Decrease of SBP/DBP (24h BP)*	*amlodipine + hydrochlorothiazide Δ 17.1mmHg*<br>*amlodipine + perindopril Δ 18.1mmHg*<br>*perindopril + hydrochlorothiazide 14.2mmHg*		
Evidence for low-dose combination (exemplary)				
Chow, Lancet, 2017 (47)<br>*- quadruple/vs placebo (N=21)*	*Decrease in office BP*	***Quadruplepill****(Irbesartan37.5mg, amlodipine1.25mg, hydrochloorthiazde 6.25mg, atenolol 12.5mg)*<br>*Δ 22/13mmHg*	***Placebo***<br>*-/-*	

DBP diastolic blood pressure, SBP systolic blood pressure, HCT hydrochlorothiazide, Dil Diltiazid, Cap Captopril, Nif Nifedipin, Ver Verapamid, Ena Enalapril, CTD Chlorthalidon, ATL Atenolol

In the latest guidelines, cornerstone of combination therapies in most clinical settings are RAS-I combined with TZD or calcium-channel blockers. In black people monotherapy with RAS-I is less effective in blood pressure control than in white persons (42, 73), but in combination with a diuretic the effect seems to be similar (74, 75). In a recent trial, the combination of perindopril and hydrochlorothiazide was clearly inferior to regimens containing a calcium channel-blocking agent(60).

The benefits of RAS-I for kidney outcome have been well documented in individuals of African descent (53, 76, 77), which is important due to higher prevalence of renal impairment in sub-Saharan Africa(78).Cardiac hypertensive-mediated organ damage like hypertensive heart disease, which is highly prevalent in black people with hypertension had been linked to RAS activation, too (4). Therefore RAS-I based combination therapy may have an incremental value compared to other non-RAS based combination therapies.

Due to a higher risk of angioedema of ACE-I in individuals of African descent(79) we chose to use an ARB.

Arm B=Intervention 2: Participants will be started with low dose (1/4) triple combination treatment with CCB, TZD and ARB. If uncontrolled after 4 weeks*, dosages of all drugs will be doubled. If after 8 weeks still uncontrolled* dosage will be increased to full dose of all three drugs.

***i.e. if target blood pressure is not achieved at this time point (target blood pressure defined as clinic BP ≤130/80mmHg in patients <65years and ≤140/90mmHg in patients ≥65years). See Figure 3 for details including dosing.*

***Rationale for Arm B:** Combination treatments are more effective in reducing blood pressure compared to a single drug regimen (4, 46, 47, 56, 80), as pointed out above. Additionally, dosage increases in monotherapy are less effective than combination of drugs with different mechanisms of action(46) and prone to more side effects than lower dose combinations (81). Therefore, lower-dose combination treatments may achieve a better blood pressure control with better tolerability. Furthermore, it has been shown, that a rapid control of blood pressure is important for prevention of cardiovascular endpoints (82). Another reason to quickly achieve blood pressure control, specifically in a sub-Saharan African setting is a reduced number of necessary clinic visits, as transport to clinics and time for clinic visits are major issues for patients in rural Africa for financial reasons and absence from daily responsibilities. We decided not to include a betablocker as effect on arterial hypertension was minor in other studies (83, 84).*

Additional aspects

We chose as a target blood pressure a systolic pressure of ≤130/80mmHg in patients <65years of age and ≤140/90mmHg in patients ≥65years of age for the clinic blood pressure in line with updated guidelines and because of beneficial effect on cardiovascular endpoints(48).

In summary, evidence from high income countries shows better efficacy and tolerability of combination over monotherapy especially in single pill approaches. As very few studies have compared the different regimens to the WHO standard in sub-Saharan Africa, it remains to be proven that a combination treatment is more effective and cost-effective in blood pressure control.

3.2. Primary and Secondary endpoints

3.2.1. Primary Endpoint

- Proportion of patients reaching a target blood pressure (clinic blood pressure) of ≤130/80mmHg in patients <65years of age and ≤140/90mmHg in patients ≥65years of age at 12 weeks after enrolment (with age at enrolment defining the target BP throughout the study)

3.2.2. Secondary Endpoints

- Proportion of patients reaching a target blood pressure of ≤130/80mmHg in patients <65years of age and ≤140/90mmHg in patients ≥65years of age at 4, 8 and 24 weeks after enrolment
- Reduction in blood pressure (change from enrolment) at 4, 8, 12, 24 weeks
- Proportion of patients with treatment adaptations made to the primary treatment (dose increases and/or drug additions) within 12 weeks
- Number of treatment adaptations per patient made to the primary treatment within 12 weeks
- Time until (first*) target blood pressure of ≤130/80mmHg in patients <65years of age and ≤140/90mmHg in patients ≥65years of age, with censoring at last visit for those not observed to reach the target (*we ignore any subsequent rebounds above target blood pressure)
- Proportion of patients with changes in surrogate markers for hypertension-mediated organ damage within 24 weeks (resolving, newly occurring or worsening):
    - Kidney impairment: changes in glomerular filtration rate, measured by CK EPI formula, increase in proteinuria according albumin/creatinine ratio
    - Signs of hypertensive heart disease assessed by electrocardiogram (positive Sokolow-Lyon Index defined as Sokolow-Lyon voltage (SV_1 + RV_5/V_6 ≥ 3.5 mV and/or RaVL ≥ 1.1 mV)(85, 86))
    - Hypertensive cardiopathy assessed by remote echocardiography showing signs of left ventricular hypertrophy(87)
    - Left atrial remodeling assessed by remote echocardiography showing left atrial enlargement(88, 89)
    - retinopathy assessed by retinal picture (90)
- Proportion of patients with major cardiovascular endpoints such as death, stroke, myocardial infarction, heart failure) within 24 weeks
- Proportion of patients lost to follow up or stopped treatment within 24 weeks
- Proportion of patients with at least one grade 3/4 adverse event within 24 weeks
- Proportion of patients with at least one severe adverse event within 24 weeks
- Proportion of patients who were non-adherent to drugs at 12 weeks (<90% pill count or missing pills ≥ 2 times in the last month, or drug intake different from prescription ~~<90% of self-reported drug intake,~~)
- Reasons for non-adherence assessed by pill count and self-report (descriptive analysis)
- Cost-effectiveness of the 3 treatment algorithms

<u>Substudy A (100 patients in Ifakara)</u>

- Proportion of patients with white coat hypertension, as determined by 24h ambulatory blood pressure measurement
- Proportion of patients with blood pressure control determined by 24h ambulatory blood pressure measurement (24h mean blood pressure <130/80mmHg irrespective of age)

3.2.4. Effect modification

We will assess effect modification of the primary outcome (proportion of patients reaching target blood pressure as defined above at 12 weeks) by:

- Site (Tanzania/Lesotho)
- HIV status (positive/negative). While HIV-infection is not expected to change response to anti-hypertensive drugs, comedication with antiretrovirals might affect drug levels

3.3. Study Design

3.3.1. General study design and justification of design

We will conduct an open-label, randomized controlled two-country trial to compare the effectiveness and cost-effectiveness of 3 different antihypertensive treatment strategies in HIV-positive and negative patients with uncomplicated arterial hypertension in Tanzania and Lesotho.

3.3.2. Methods of minimizing bias

Randomization

Eligible and consented patients will be consecutively enrolled and randomized in a 2:1:2 ratio to the dual combination, triple combination or control arms, respectively. Randomisation will be stratified by site (Tanzania, Lesotho), HIV status (positive, negative), and age (<65 years/≥65 years of age), using permuted blocks with varying block size. A randomisation list will be prepared in advance by an independent statistician. The randomisation list will be stored securely on a server with restricted access.

The sequence will be concealed by using envelopes prepared by an independent person (based on the list prepared by the independent statistician). The envelopes will be opaque and sealed, labelled only with the stratification information and a sequential identification number. Randomisation of a patient will be performed once the eligibility and consent of the patient have been confirmed and entered into the database, therefore maintaining concealment of allocation. Inside the envelope will be a piece of paper confirming the stratification information and sequential identification number, and indicating the arm allocation and subject randomization number. The envelopes will be stored securely. Checks will be performed intermittently during enrolment to ensure that the randomization sequence is respected.

Blinding

This will be an open-label study without blinding. Persons who perform tracking of patients who have missed scheduled visits will remain blinded to the randomised allocation of the patient. Also, the cardiologist and ophthalmologist interpreting results of examinations such as echocardiography,24h blood pressure measurement and retinal picture will be blinded to the study group.

3.4. Study Intervention

3.4.1. Overview of the 3 treatment strategies

Figure 2 gives an overview of the three treatment strategies.

Figure 2. Overview of the three treatment strategies.

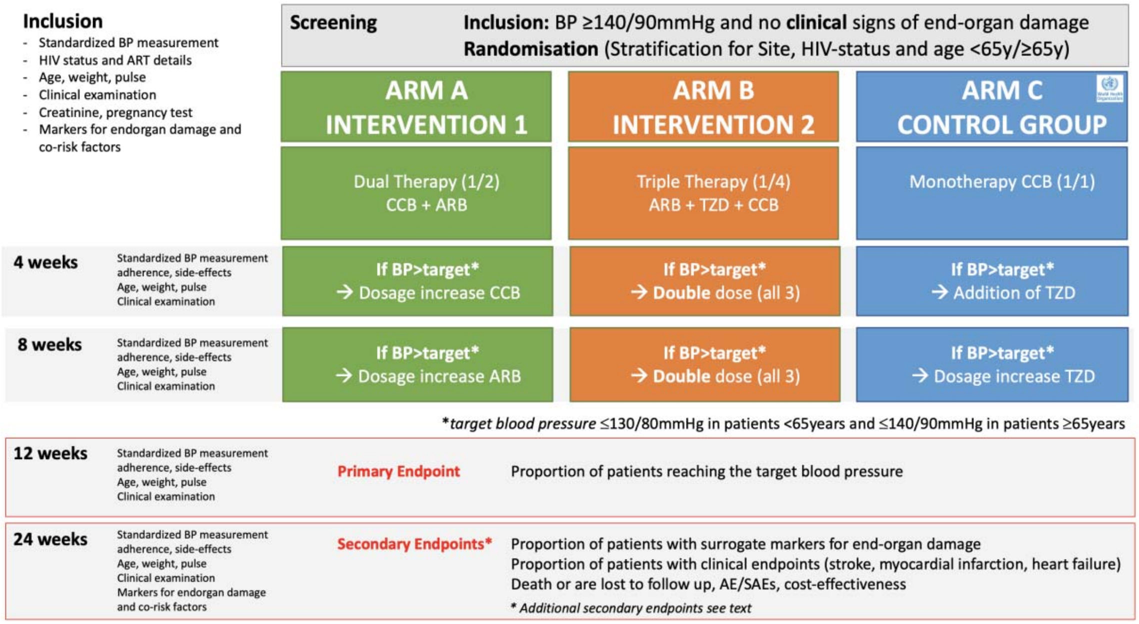

3.4.2. Intervention and Control Arms

The choice of drugs is adapted to available drugs in both settings following a pragmatic approach (Figure 3):

Figure 3. Dosing for each arm.

	Arm A<br>Intervention 1	Arm B<br>Intervention 2	Arm C<br>Control (Standard of Care)
Start	Amlodipine 5mg OD +<br>Losartan 50mg OD	Amlodipine 2.5mg OD +<br>Losartan 12.5mg OD +<br>Hydrochlorothiazide 6.25mg OD	Amlodipine 10mg OD
Week 4$	Amlodipine 10mg OD +<br>Losartan 50mg OD<br>*	Amlodipine 5mg OD +<br>Losartan 25mg OD +<br>Hydrochlorothiazide 12.5mg OD<br>*	Amlodipine 10mg OD +<br>Hydrochlorothiazide 25mg OD<br>*
Week 8$	Amlodipine 10mg OD +<br>Losartan 100mg OD<br>*	Amlodipine 10mg OD +<br>Losartan 50mg OD +<br>Hydrochlorothiazide 25mg OD<br>*	Amlodipine 10mg OD +<br>Hydrochlorothiazide 50mg OD<br>*
Week 12-24$	Amlodipine 10mg OD +<br>Losartan 100mg OD<br>*, **	Amlodipine 10mg OD +<br>Losartan 50mg OD +<br>Hydrochlorothiazide 25mg OD<br>*,**	Amlodipine 10mg OD +<br>Hydrochlorothiazide 50mg OD<br>*, **

$ Increases in dosages only if target BP is not reached (see above)
* In case of orthostatic hypotension or adverse events, medication will be reduced to the prior step – or to half of the initial dosage.
**if regimen shows insufficient effect, individualized adaptation possible according the treating physician

Patients not reaching blood pressure control at week 12

After the primary outcome of blood pressure response at 12 weeks, treatment will be continued until week 24 for all patients. For patients not responding to treatment at this time point, a recommendation for adaption will be made to the treating physician. Drug dosages can be increased or additional drugs added, e.g. arm A and C to triple therapy and Spironolactone 25mg once daily in arm B might be added according to the decision of the treating physician.

Accountability of drugs will be assured using drug logs of purchased and dispensed drugs. All drugs used within this trial are locally available in both countries and will be dispensed to patients for free for the duration of 6 months. After closure of the study, patients will be directed to routine care. This will be fully explained in the patient information sheets provided before informed consent is sought. Healthcare authorities will be informed on the trial results in order to be integrated into National Guidelines, if applicable.

4. Study population and Study Procedures

4.1. The research sites

The trial will be conducted in two sites: the St. Francis Referral Hospital, Ifakara, Tanzania and Mokhotlong District Hospital in Lesotho:

Chronic Diseases Clinic and Emergency Department St. Francis Referral Hospital, Ifakara

The Chronic Diseases Clinic of Ifakara (CDCI) was funded 2004 as the Care and Treatment Center for patients with HIV from Kilombero and Ulanga Districts in rural Tanzania. The CDCI is located in close proximity to the Emergency department of the St. Francis Referral Hospital and is a collaborative project of the hospital, the Government of Tanzania, the Ifakara Health Institute, the Swiss Tropical and Public Health Institute and the University Hospital Basel. It offers clinical services according National AIDS Control Programme, has integrated TB services and a program specializing on care for HIV-affected families (One Stop Clinic). Clinical data is captured within an electronic database (OpenMRS). The CDCI runs a patient cohort since its start – the Kilombero and Ulanga Antiretroviral Cohort (KIULARCO), which serves as a basis to better understand needs of patients living with HIV in the Kilombero and Ulanga District. It contains clinical and laboratory information of more than 10,000 included patients connected to a biobank. Currently about 4,500 HIV-positive patients are under active care. Details of the cohort have been published elsewhere (61, 62). In the Emergency department approximately 36’000 patients are seen per year (91).

Mokhotlong Government Hospital (MKGH), Lesotho

Butha-Buthe and Mokhotlong district are located in northern Lesotho, Southern Africa. Both districts are characterized by mostly rural settings with an estimated population of 220,000, mainly living in villages scattered over a mountainous area of 5,842 km^2. Each district has a small town with about 20,000 habitants where the district hospitals are located. The outpatient department of Butha-Buthe hospital attends to 200 to 300 patients per day, the one in Mokhotlong 150 to 250 patients per day.Both sites are supported by the Swiss not-for-profit organization SolidarMed and have a close research collaboration with the Swiss Tropical and Public Health Institute (see section 2.2). Recruitment will take place at MKGH. In case of recruitment challenges, recruitment can be extended to Butha-Buthe district.

In both sites, the pre-screening procedures might be expanded to community events by implementation of the following: a) radio announcement of the study with invitation of people to come for free blood pressure measurement b) information of the study at churches and mosques c) information of nearby health centres to bring patients with high blood pressure to the study site. Study-related procedures such as screening and enrolment will remain at the study sites.

4.1.1. Inclusion Criteria

Adults (≥18 years of age) of African descent and black ethnicity with confirmed uncomplicated and currently untreated arterial hypertension (definition and measurement see chapter 4.3.3) diagnosed at one of the two sites.

4.1.2. Exclusion Criteria

- Current hospitalization for any reason
- Refusal of an HIV-test or indeterminate HIV test result
- History of cardiovascular event in the last month (anginal pain, stroke, myocardial infarction or diagnosis by a doctor)
- Symptomatic arterial hypertension (blood pressure ≥180/110mmHg plus acute headache or chest pain) or acute cardiovascular event (see above)
- acute disease, e.g.:
    - fever >37.5°C or other signs of acute concomitant infection
    - dyspnea/respiratory distress
    - acute pain
- Clinical signs of hypertension-mediated organ damage
    - heart failure (bilateral pitting edema, bilateral crackles or pleural effusion, distended jugular veins)
    - ischemic heart disease (anginal pain on exertion)
    - signs of current ischemic/hemorrhagic stroke (hemiparesis, loss of consciousness)
- Pregnancy (test required for females 18-45y of age)
- Non-consenting or inability to come for follow-up visits
- Creatinine clearance ≤30ml/min by the Chronic Kidney Disease Epidemiology Formula (CK-EPI) estimation and measurement with a point-of care creatinine from capillary blood

4.2. Recruitment strategy, screening and informed consent procedure

In Ifakara, patients will be recruited from the Chronic Diseases Clinic Ifakara (HIV-positive) and from the Emergency Department (HIV-negative) of the SFRH. Currently, about 26% of patients under regular care within KIULARCO have an arterial hypertension (429 adults). Many of these patients are not taking a blood pressure lowering therapy due to financial constraints, even if prescribed, and will be eligible for this study. Within the Emergency department of non-HIV-diagnosed patients we expect a similar if not higher frequency, as the yearly consultation numbers of the Emergency department is 36'000 patients, of those 75% adults and 1148 with a hypertensive emergency (91). From a community survey (http://ihi.eprints.org/2872/1/LONGITUDINAL_COHORT_STUDY.pdf prevalence of arterial hypertension is high at 30% (>120/80mmHg). In view of these high numbers we are confident to include the targeted sample size of 634 patients for this site within 12 months.

In Lesotho, adult prevalence of arterial hypertension has been reported to be 31% among 25 to 64 year olds(92). Among adult persons living with HIV in Butha-Buthe the prevalence was 28% and 22% in females and males respectively during a study conducted in 2014 (69). The two study sites, Butha-Buthe and Mokhotlong district hospitals, have about 6,000 patients living with HIV in regular care. Moreover, as mentioned under section 2.3.5, in addition to patients living with HIV, both hospitals have an outpatient department with 200 to 300 outpatient visits per day.

At Ifakara, patients receive routine BP measurement in the triage of the Emergency Department. In Lesotho, patients receive routine BP measurement at different units of outpatient department at MKGH. In both sites, the pre-screening procedures might be expanded to community events. Patients with a BP measurement ≥140/90 mmHg will be referred to the study nurse at each site who thereafter

will remeasure blood pressure as outlined in section 4.3.3. If elevated BP is confirmed, the study nurse will assess the patient for eligibility and if eligible enrolment will be done by the study physician. Given the high prevalence of elevated BP in the general as well as in the HIV infected population, we are confident to recruit about 6-8 study participants per day in both sites (3-4 per site), aiming at completion of targeted sample size within 1 year.

Each site will enrol at least 500 participants, after which enrolment will be competitive.

The study nurse will obtain written informed consent from all study participants, before the participant is undergoing screening. All study participants are 18 years or older. The investigators will explain to each participant the nature of the study, its purpose, the procedures involved, the expected duration, the potential risks and benefits and any discomfort it may entail. Each participant will be informed that the participation in the study is voluntary and that he/she may withdraw from the study at any time and that withdrawal of consent will not affect his/her subsequent medical assistance and treatment. Study participants will not receive any payment to be part of the study besides compensation for transport expenses for additional clinic visits.

The participant will be informed that his/her medical records may be examined by authorized individuals other than their treating physician (e.g. domestic and foreign regulatory authorities, sponsor's monitors and auditors). All participants for the study will be provided a participant information sheet and a consent form describing the study and providing sufficient information for participant to make an informed decision about their participation in the study. There will be enough time given to the participant to decide whether to participate or not. The patient information sheet and the consent form has been submitted to the CEC and to the competent authority to be reviewed and approved. The formal consent of a participant, using the approved consent form, will be obtained before the participant is submitted to any study procedure.
The participant will read and consider the statement before signing and dating the informed consent form, and will be given a copy of the signed document. Consenting will include separate checkboxes for screening procedures, enrolment and participation for 24h BP measurement for the subgroup study in Ifakara. The consent form will also be signed and dated
by the study nurse and investigator (or his designee) and it will be retained as part of the study records. All patients will sign an informed consent form, which is available in local and English language (see APPENDIX I).
If the patient is illiterate, the study information will be read to him or her in the presence of a valid witness. To document consent of an illiterate participant, the signature of a witness and the fingerprint of the participant will be obtained. Non-consenting patients will not be included in the study.

Assignment to Study Arm

The study nurse will assess participant eligibility and obtain consent. After confirmation of eligibility by the study investigator he/she opens the envelope according site, HIV status and age, randomization. The envelope contains the information on the allocation to the treatment arm, which the study nurse will enter directly in the database and be shown by MACRO on any subsequent visit. Since this study is not blinded, the study team, the treating physicians, and the patient will know the arm to which he or she has been allocated.

4.3. Study Procedures

4.3.1. Study Schedule

Figure 4 illustrates the study schedule (procedures and CRFs)

Figure 4: Study schedule

Study Periods	Baseline (screen & Enrolment)		Intervention Period			End of Study
Visit	SV01	ER01	FUP01	FUP02	FUP03	EOS01
Timepoint week<br>day<br>window for visit (days)	Day 0*	Day 1*	Week 4<br>Day 28<br>(21-42)	Week 8<br>Day 56<br>(50-70)	Week 12<br>Day 84<br>(78-98)	Week 24<br>Day 168<br>(141-196)
Patient Information &Informed Consent	x					
Demographics	x					
Medical History	x					
In- /Exclusion Criteria	x					
Physical Examination	x	X	X	X	x	x
Vital Signs		X	X	X	x	x
Blood pressure measurement	x		X	X	x	x
Screening blood tests (fingerpick)<br>- HIV Test (rapid test as per national GL)<br>- Point of care creatinine	x					
Urine pregnancy test in females 18-45y	x		$X^{£}$	$x^{£}$	$x^{£}$	$x^{£}$
Laboratory testing (venipuncture)<br>- Blood: Full blood picture, creatinine, ALT,<br>- Blood storage for biomarkers<br>- Urine: Alb/Creat ratio, dipstick		X				x
Remote echocardiography		X				x
Remote fundoscopy		X				x
24hour blood pressure measurement		x**			x**	
Randomization		X				
Administer Study Medication		X	x	X	x	
Assess concomitant therapy or interventions		X	x	X	x	
Assessment of adverse Events		X	x	X	x	x

**day 0 (screening) and day1(enrolment) can be the same day, £for women of childbearing age, ** in 100 patients from Ifakara (substudy).*

eCRFs							
000	Pre-Screening sheet (routine)	X					
001	Screening Form (SN)	X					
002	Enrolment Form (SN)		X				
003	Examination Form (SD)		X	x	X	x	x
004	Lab request sheet (paper) (SN)		X				x
005	24h BP form (SN)		X			x	
006	Randomization form (SN)		X				
007	Drug prescription form (SD)		X	x	X	x	
008	Drug dispensing form (SN)		X	x	X	x	
009	Lab result form (SN)		X				x
010	Follow-up Vital Form (SN)			x	X	x	x
011	FUP vital form (SN)			x	X	x	x
012	Visit form (SN)			x	X	x	x
013	Continuing form (SN)		X	x	X	x	
014	End of study form (SN)			**	**	**	x
015	AE form (SD)			*	*	*	*

016	SAE form (SD) (paper and eCRF)			*	*	*	*
017	Tracking form (SN)			***	***	***	***

SN study nurse, SD study doctor
**to be completed in the event of an AE/SAE*
***to be completed as needed, ie in event of death, lost to follow up or patient withdrawal*
****to be completed as needed, ie if patient does not attend visit*

4.3.2. Procedures at each visit

Overview over study procedures

Figure 5 gives an overview of the study procedures.

Figure 5. Overview of study procedures.

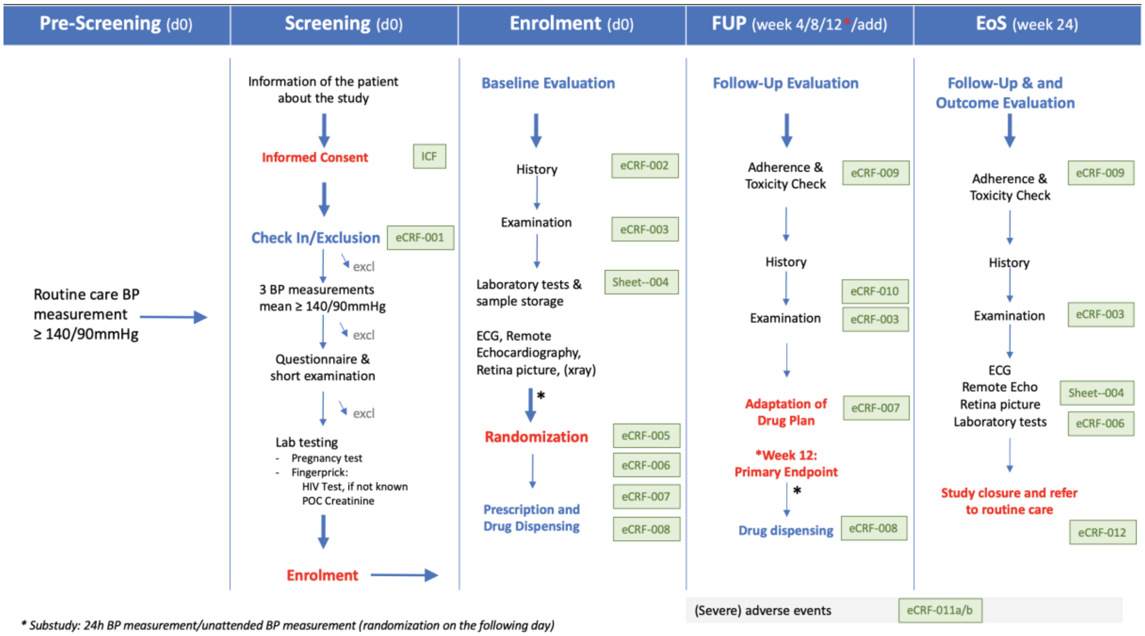

Pre-Screening (D0)

During a pre-screening visit, patients get a blood pressure measurement during routine care at the Emergency and Outpatient department or specifically planned screening events in the community or at other health centers. In patients with a BP ≥140/90mmHg, the measurement will be documented on a numbered paper form and the patient will be referred to the study nurse for further blood pressure measurement and additional evaluations.

Screening (D0)

Patients will be informed about the study and consented first. Non-consenting patients or consenting patients with present exclusion criteria will be directed to routine care for further management if needed.

Screening of patients will be done in a stepwise procedure by the study nurse and the MD. The study nurse takes a short history, measures clinic BP according to a detailed SOP (see below). In a specific study room and she confirms the patients' ability to come back for follow-up visits. If qualifying, the MD will do a short clinical examination (exclusion of symptomatic hypertension, heart failure, ischemic heart diseases or other acute conditions). The nurse will perform and a blood glucose from capillary blood

(fingerprick) and for women of child-bearing age (18-45 years) a urine pregnancy test. If the patient is not known HIV positive or has a documented HIV test within the last 3 months (written documentation), at the Ifakara site an HIV test according government recommendations is done. At Lesotho site, the patient will be sent to the testing facility. In addition, a point-of care creatinine test is done from capillary blood or a venepuncture to exclude patients with a creatinine clearance of ≤30ml/min (CK-EPI, calculated from creatinine value entered in MACRO) for all patients who do not have a documented creatinine test within the last 2 weeks before enrolment.

Enrolment (D0)

Baseline information containing demographic information and clinical evaluation including BP measurements as well as vital signs are filled into standardized questionnaires within the electronic data management system. Five ml of blood are then withdrawn and sent with a routine lab request form to the laboratory for full blood picture, creatinine, and liver values. Blood will be stored for further analyses at -80° (e.g. HbA1c, lipid panel, brain natriuretic peptide, troponin T). The patient will be instructed on how to deliver a mid-stream urine sample and the urine sample will be sent to the laboratory for urine dipstick analyses. The Albumin/Creatinine ratio will be determined using the POC Alere Afinion AS100 Analyzer. In Ifakara site the test is done in the laboratory. In Mokhotlong site it will be done at the study room by the study nurse. The urine sample can be kept for 7 days at -4° or stored for longer time periods at -80°.

A 12-lead-ECG and a focused focused echocardiography according to a standard operating procedure will be done and evaluated by the study team. The site investigator evaluates the ECG for the presence of and ST elevation or a conduction block and if one of these signs is present, a senior physician is contacted for advice on whether drugs can be administered safely. ECG and echocardiography will be stored and interpretation done at a later time point by a blinded cardiologist. A retinal picture (iExaminer, Welch-Allyn) will be done by the study nurse or site investigator. Pictures will be stored electronically and interpreted at a later time point by a designated ophthalmologist. They will not affect further management within the study.

The lab results will be collected on a paper form as per laboratory routine and entered, once completed, into the MACRO database and confirmed by the study nurse. For Ifakara site lab technician enters the lab results while at Lesotho site, the study nurse enters the lab results once confirmed by MD. Source files remain at the laboratory and a copy in the patient file. The results will be communicated to the patient during the next visit, if within normal or clinically irrelevant range. If abnormal and clinically relevant, the patient will be called to come back, and he/she will be attended by the study doctor.

Patients in Ifakara will be offered a 24h blood pressure and unattended office blood pressure measurements (up to a number of 100 patients). If patients do consent, these measurements will be done as per description in section 4.3.3. Consent needs to be given in a separate field in the informed consent form, separate from the general study participation informed consent. Also, patients can withdraw from the 24h BP measurement without being stopped for other study-specific procedures.

The patient will be randomized after assessment of these baseline information and allocated to one of the 3 study arms by opening the envelope with the allocation. The patient receives drug for a full month with clear instructions on intake and an appointment for the next follow-up visits (leaflet). Drug packages will be labelled as study medication using stickers. All information and results will be entered into the MACRO database.

Follow-up visits (weeks 4, 8, 12)

Follow-up visits will be scheduled 4, 8 and 12 weeks after enrolment. Time windows will be allowed as follows: week 4=day 28 (window day 21-42), week 8= day 56 (window day 50-70), week 12=day 84 (day 77-98), week 24=day 168 (day 141-196). On these follow-up visits, the study nurse assesses adherence as follows: what is the pill count left, how many times did the patient miss pills and what were the

reasons for not taking pills). Also, she will ask for symptoms relating to side-effects and other adverse events). Standardized BP measurements to determine clinic BP will be done as per SOP and the MD will examine the patient clinically. In patients who do not reach the target BP and who do not report side effects despite intake of medication as prescribed, medication will be adapted as per study protocol. If the patient reports being off drugs for the whole period, the previous regimen and dosage will be restarted. In patients who reach the BP target, medication will be continued in the same dosage. In patients with side-effects or other clinical events an adverse event form will be filled. Drug prescription will be adapted individually by the study physician. In case of orthostatic hypotension or adverse events, medication will be reduced to the prior step – or to half of the initial dosage. In women of childbearing age (18-45years), a pregnancy test will be repeated at every visit. If the test is positive, Losartan and Hydrochlorothiazide must be stopped, Amlodipine can be continued. If Amlodipine is not sufficient for blood pressure control (dosage can be increased to 10mg), a combined alpha/betablocker, preferable labetalol is the preferred agent to add if available. If not, then metoprolol (long-acting formulation) or labetalol as well as Methyldopa are treatment options according National Guidelines of the respective country. The patient remains in the study, costs for drugs are to be reimbursed (labetalol or metoprolol), if not provided by the government for free and the patient has to be scheduled as planned until 6 months. The sponsor-investigator must be informed and the email printout filed in the patients file.

Drugs will be dispensed as per study protocol for one month at week 4 and 8 and for 3 months at week 12, if the patient is clinically stable. Additional visits can be scheduled if clinically indicated.
For above mentioned subset of patients included in the 24h BP substudy, the 24h blood pressure and unattended office measurements will be repeated at week 12.
Patients missing their appointment will be tracked within a week of the missed scheduled appointment. A phone call will be done first, if the patient is not reachable, tracking with the help of community health workers and physical tracking will be done. For this, an automatically generated list from MACRO will be dispensed to a tracking person, who is blinded to the intervention arm. Tracking activities are documented in a specific MACRO form.

Follow-up visit (week 24)

On the last follow-up visit, which is scheduled for 24 weeks (allowing a time window of +/-4 weeks, in addition to the procedures as described under 9.3.4., the patient will receive laboratory and clinical examination, an ECG, Echo and retinal picture, as described for baseline visit. After successful completion of the study, he/she will be referred to the local medical team for ongoing management including further prescription of drugs.

4.3.3. Measurements and definitions of blood pressure

Correct Measurement of Blood pressure

Correct measurement of blood pressure is a complex task with a 1C Grade evidence for a standardized office measurement and 1A Grade for Out-Of-Office and Self-Monitoring of blood pressure (36). White coat hypertension has shown to be high in an African setting at 15% in a recent meta-analysis (93). On the other hand, 24h-ambulatory blood pressure measurement in a rural population coming from distant areas to the clinic is often not feasible. One randomized study showed comparable differences in blood pressure reduction resulting from office and ambulatory blood pressure measurement (60). For the determination of clinic blood pressure we will use a standard operating procedure based on the ESC-ESH Guidelines for the prevention of arterial hypertension 2018 recommendation (48).
In brief, before BP measurement, arm circumference in cm is measured and the size of cuff is determined according recommendation of the BP machine manufacturer. Blood pressure will be measured in sitting position after 5 minutes of rest with feet on floor, back supported, no caffeine, exercise or smoking in the 30min before measurement, emptied bladder, no talking during

measurement, comfortable clothes, arm supported (e.g. on table) with a validated blood pressure machine. At the screening visit,2 parallel measurements will be done on each arm to determine the arm with higher values. The reference arm (with the higher blood pressure) will be noted and used for all further clinic BP measurements during the study. For the determination of clinic BP three consecutive measurements will be done, spaced 1-2 min apart and the mean value of the 2nd and 3rd measurement will be calculated. This type of clinic BP has been used in several recent clinical trials and epidemiological studies and is based on the ESH 2018 recommendation(94-96). For better comparability and standardization of clinic BP results during the trial, we do not implement the recommendation of a fourth measurement when the delta of BP is more than 10 mmHg between a first and a second reading.

For blood pressure measurement we will use Omron M6 Comfort [HEM-7321-E] using individualized cuff size, which has been validated in similar settings (97).

In a subset of patients in Ifakara (the first 100 patients enrolled, living close to the clinic), we will offer 24hour blood pressure and unattended blood pressure measurement. For consenting patients (special section on informed consent) these additional measurements (see below) will be started immediately after enrolment, before randomization and drug dispensing. The aim is a comparison between these BP measurement methods in order to assess the proportion of patients with white coat hypertension(60). For 24 h ambulatory BP measurement, the device will be programmed to take measurements every 20 min between 6 and 22:00 and every 30 min between 22:00 and 6:00 (98).

Before supplying the patient with the 24h blood pressure measurement device an unattended automated office blood pressure measurement will be done: patients will be at rest in a separate room and after an initial test measurement to ensure device functionality the patient will be left alone. The AOBP device will be programmed to take 5 consecutive measurements after 5 min of rest, spaced 1 min apart and will calculated the mean out of all three measurements (98-102). Both 24h blood pressure measurement and unattended blood pressure measurement will be repeated at 12 weeks. Results of these additional measurements will be entered into MARCO, but will not affect randomization and drug dispensing. Patients will be informed of the result at the end of the study

Arterial hypertension:

We chose to use the ESC-ESH Guidelines for the prevention of arterial hypertension 2018 (48) for definition of arterial hypertension, which is in line with the previous guidelines and the definition by the WHO:

- Grade I hypertension: systolic blood pressure of 140-159mmHg or diastolic blood pressure of 90-99mmHg.
- Grade II hypertension: systolic blood pressure of 160-179mmHg or diastolic blood pressure of 100-109mmHg.
- Grade III hypertension: systolic blood pressure of ≥180mmHg or diastolic blood pressure of ≥110mmHg.

4.4. Withdrawal/discontinuation

All patients will remain within the study, unless they specifically withdraw informed consent, which will be documented accordingly. Withdrawal will be recorded separately for withdrawal from treatment, from follow-up, from using data, or from 24h blood pressure measurements.

5. Statistics and Methodology

5.1. Statistical Plan and sample size calculation

We hypothesize that the proportion of patients reaching the primary endpoint will be higher in the triple combination arm compared to the control arm. Additionally, we hypothesize that the dual combination will be non-inferior to the control arm.

5.1.1. Determination of Sample Size

Based on evidence coming from literature mentioned under 3.1.3 we made the assumptions as shown in Table 3.

Table 3. Assumptions for sample size calculation.

	**ARM A (Intervention 1)**<br><br>Guideline + incremental value of ARB in African patients	**ARM B (Intervention 2)**<br><br>Quadruple quarter dose	**Arm C (control)**<br><br>WHO standard of care
EVIDENCE FROM LITERATURE			
Literature in Individual of African descent	**67%**<br>*(response=DBP<90mmHg or 10% decrease(4, 103))*	**83%**<br>*(low-dose quad pill response =<135/85 mm Hg (47))*	**67%**<br>*(response= BP<140/90mmHg (59))*
Conservative effect estimation for higher target BP[£]	**60%**	**75%**	**50%**
Conservative effect estimation for lower target BP[$]	**40%**	**55%**	**40%**
Notes	- Assumption of a smaller effect due to lower BP target	- Effect might be lower (3 drugs instead of 4; no single pill)<br>- Effect might be higher as allowance to increase dosage	- Effect might be lower due to lower target

[£]≤140/90mmHg; [$]≤130/80mmHg

The response rate in the control arm is assumed to be 40%. For the superiority comparison between the triple combination and control arms, we assume an improvement in the triple combination arm of 15% (two-sided alpha of 0.05). For the non-inferiority comparison between the dual combination and control arms, we assume a non-inferiority margin of 10% (one-sided alpha of 0.025). A sample size of 431 patients in each of the control and dual combination arms, and 216 patients in the triple combination arm, yields a power of 85% for the non-inferiority comparison and a power of 95% for the superiority comparison. The overall sample size is therefore 1078 patients, with the randomization ratio of 2:1:2 for the dual combination, triple combination and control arms, respectively. Assuming 15% of participants will become lost-to-follow-up(60) brings the total required sample size to 1268 individuals.

5.1.2. Planned analyses

Primary Analyses

Statistical analysis will be performed by the trial statistician using Stata. Analyses will follow CONSORT guidelines (104-106) and intention-to-treat principles, that is including all participants as randomized

except as detailed below. A flowchart will describe the inclusion and follow-up of participants by study arm. Baseline characteristics will be described by study arm with summary statistics such as median and interquartile range or number and percentage; no formal testing between arms will be performed (107). Outcomes will be described by arm using summary statistics. The primary outcome, the proportion of patients reaching the target blood pressure within 12 weeks, will be assessed using a logistic regression model, reporting odds ratios. Binary secondary outcomes will be evaluated in the same way. Continuous secondary outcomes will be assessed using linear regression models, reporting adjusted mean differences between arms. Time to event outcomes will be assessed using appropriate methods, such as Kaplan-Meier estimation and Cox proportional hazards models. All estimates will be reported with 95% confidence intervals (CI). Effect modification of the primary outcome by site and HIV status will be assessed by incorporating an interaction between arm and site or HIV status, respectively, acknowledging that power will be low. All models will be adjusted for baseline blood pressure and the stratification factors of site, HIV status and age (108). Primary analyses will be complete case; we may consider appropriate methods to account for missingness such as adjusting for further baseline variables which are associated with missing outcome data or multiple imputation as appropriate (109). We will exclude pregnant women from the primary analyses, since blood pressure control and recommended medication might be different; few pregnancies are expected. We will compare each of the intervention arms versus control. For the non-inferiority comparison between the dual combination and control arms, a CI approach will be used. A figure illustrating the CIs and the non-inferiority margin will be presented. Primary analyses for the non-inferiority comparison will be performed on both the ITT and per protocol sets (109). If the dual combination is found to be non-inferior to the control, then we will assess for superiority using the ITT set. Further details will be provided in the statistical analysis plan.

Interim Analyses

An independent data monitoring committee (IDMC) will be established to monitor the trial for efficacy and safety. An IDMC charter will be developed. The IDMC will consist of at least three people, including at least one clinical expert and at least one statistician. Before or soon after the trial start, the IDMC will meet to agree on the charter, determine the schedule for subsequent meetings, and discuss the format of the interim analysis reports. A (first) interim analysis is planned for when 50% of the target sample size should have completed their primary outcome assessment at 12 weeks, which is expected to be approximately one year after the start of the trial. Whether further analyses are needed, and the timing of such analyses, will be determined by the IDMC. The interim analyses will be prepared by the trial statistician and will include efficacy and safety data. Results by arm will only be seen by the IDMC, and in particular not by any members of the trial team who are involved in the recruitment or follow up of participants. No p-value adjustment will be done to account for the interim analyses. A conservative approach such as the Haybittle-Peto rule will be adopted; more details will be included in the charter.

Cost Effectiveness

We will follow the JAMA guidelines on cost-effectiveness and calculate incremental cost-effectiveness of the three regimens from both a health-systems and from a societal perspective (110). Health systems cost will include total medication cost as well as staff time and a fixed cost for each facility visit, which will be compared to the total health benefits achieved by the three arms (111). Medical cost will be directly collected at the facility level in the two sites; we will also obtain WHO reference prices for the respective drugs and treatments for comparison. For the societal perspective, we will include additional private cost of patients, with a particular focus on out-of-pocket expenditure for visits to facilities (transport, overnight stays) as well as costs for additional medication needed and days of work lost due to sickness (110). To compute incremental cost effectiveness ratios (ICERs), we will use the control arm as our reference case, and then compute the additional costs and benefits of the two intervention arms relative to this baseline scenario. Health outcomes will directly be observed over a 24 week period;

reduced morbidity will be converted to disability-adjusted life years using the 2013 Global Burden of Disease disability weight estimates (112). A separate analysis plan will be developed for the cost-effectiveness.

5.2. Handling on missing data and drop-outs

Missing baseline and outcome data will be summarized by study arm. As outlined above, primary analyses will be complete case; we may consider appropriate methods to account for missingness such as adjusting for further baseline variables which are associated with missing outcome data or multiple imputation as appropriate (109). Details will be provided in the statistical analysis plan.

6. Regulatory Aspects and Safety

6.1. Ethical Conduct of the Study

The study will be carried out in accordance to the protocol and with principles enunciated in the current version of the Declaration of Helsinki, the guidelines of Good Clinical Practice (GCP) issued by ICH, the European Directive on medical devices 93/42/EEC and the ISO Norm 14155 and ISO 14971, and the law and regulatory authority's requirements from the participating countries. The CEC and regulatory authorities will receive annual safety and interim reports and be informed about study stop/end in agreement with local requirements.

6.2. (Serious) Adverse Events

Adverse events will be captured at every regular visit and on additional unplanned visits after randomization with record of time of onset, duration, resolution, action taken, assessment of intensity, relationship with study treatment and outcome.

An Adverse Event (AE) is any untoward medical occurrence in a patient or a clinical investigation subject which does not necessarily have a causal relationship with the trial procedure. An AE can therefore be any unfavourable or unintended finding, symptom, or disease temporally associated with a trial procedure, whether or not related to it.

A Serious Adverse Event (SAE) (GCP) is any untoward medical occurrence that

- Results in death or is life-threatening,
- Requires hospitalization or prolongation of existing hospitalization
- Results in persistent or significant disability or incapacity
- Results in persistent or significant disability or incapacity, or
- Causes a congenital anomaly or birth defect

Both Investigator and Sponsor-Investigator make a causality assessment of the event to the trial intervention, (see table below based on the terms given in ICH E2A guidelines). Any event assessed as possibly, probably or definitely related is classified as related to the trial intervention.

Relationship	Description

Definitely	Temporal relationship<br>Improvement after dechallenge*<br>Recurrence after rechallenge<br>(or other proof of drug cause)
Probably	Temporal relationship<br>Improvement after dechallenge<br>No other cause evident
Possibly	Temporal relationship<br>Other cause possible
Unlikely	Any assessable reaction that does not fulfil the above conditions
Not related	Causal relationship can be ruled out
*Improvement after dechallenge only taken into consideration, if applicable to reaction	

Both Investigator and Sponsor-Investigator make a severity assessment of the event as mild, moderate or severe. Mild means the complication is tolerable, moderate means it interferes with daily activities and severe means it renders daily activities impossible

Reporting of SAEs

All SAEs are documented and reported immediately (within a maximum of 24 hours) to the Sponsor-Investigator of the study and to the local Ethical Boards and regulatory authorities. Other AEs will be recorded and Follow-up until they are resolved.

The study will be submitted to the following Ethical Committees:

- The Ethikkomission der Nordwest und Zentralschweiz, Switzerland
- the Institutional review board of IHI (IHI-RB), Tanzania
- the National Institute for medical research, Tanzania,
- the Research Coordination Unit, Ministry of Health of Lesotho
- local regulatory authorities: District medical offices in both sites

6.3. Protocol amendments

Substantial changes to the study setup and study organization, the protocol and relevant study documents are submitted to the Ethics Committee for approval before implementation. Under emergency circumstances, deviations from the protocol to protect the rights, safety and well-being of human subjects may proceed without prior approval of the Ethics Committee. Such deviations shall be documented and reported to the Ethics Committee as soon as possible. A list of all non-substantial amendments will be submitted once a year to the competent EC together with the ASR.

6.4. Early termination of the Study

The Sponsor-Investigator may terminate the study prematurely according to certain circumstances, for example:

- ethical concerns
- insufficient participant recruitment
- when the safety of the participants is doubtful or at risk
- alterations in accepted clinical practice that make the continuation of a clinical trial unwise
- early evidence of benefit or harm of the experimental intervention

6.5. Insurance

The sponsor-investigator is responsible for issuing an appropriate insurance to cover for trial-related damage for all participants (risk category A).

7. Further Aspects

7.1 Overall ethical considerations

Cardiovascular diseases are an emerging problem in SSA and fall on a health system that is only poorly prepared to respond to the challenge of CVD risk factor management in under-resourced settings (113, 114). The WHO 2013 global action plan for non-communicable diseases calls for a strong action addressing the silent epidemic of arterial hypertension and a strong pillar of action will be pharmacologic therapy for individuals with elevated blood pressure (17). However, as outlined above, current WHO guidelines on treatment of arterial hypertension are not endorsed by strong evidence from studies conducted in sub-Saharan Africa. Results of the proposed randomized controlled trial will allow improving care of patients with elevated blood pressure and thus has the potential to contribute in long-term to the reduction of cardiovascular events in sub-Saharan Africa.

Furthermore, health systems in many sub-Saharan African countries are working towards universal health coverage, increasing the chances of patients being able to adhere to long-term medication. Prices of medication heavily depend on negotiations of stakeholders with companies, if drug treatment programs are to be rolled-out.

7.2 Risk-benefit assessment

Table 4 describes the risk-benefit assessment.

Table 4. Risk-benefit assessment.

Potential Problem	Solution/Alternative	Comment
**Slow recruitment**	Extend recruitment period	Based on expected failure rates, recruitment expected to be feasible within a 12 months period
**Screening for surrogate markers not successful due to lack of local competence**	Planned on-site mentoring for several weeks each year	Capacity building for all newly applied laboratory analyses planned
**Screening for surrogate markers not successful due to lack of local competence**	Samples temporarily shipped to another laboratory facility (IHI Bagamoyo in Tanzania, South Africa for Lesotho)	
**Shortage of drug supply**	Planning of drug supply at the beginning of the trial to ensure availability throughout the study period	We will provide all drugs for the duration of the study. We will encourage and clarify options for healthcare insurance to ensure continuation of treatment after study termination
**Recruited study-participants change district or move to other areas during study**	Sample-size adjusted accordingly	A 15% LTFU-rate is included in the sample-size
**High loss to follow-up (LTFU)**	We plan tracking of patients who miss appointments with SMS reminder, telephone and physical tracking.	We expect a LTFU rate of about 15% and add this in the sample size calculation
**Drugs for NCDs are not covered vertical health programs in Tanzania and Lesotho (only in HIV-pos). During the study we provide drugs, but thereafter patients will have to buy them**	Inform and counselling about health insurance options and community funds	We expect community funds and health insurance systems to be slowly implemented over time

8. Quality Control and Data Protection

8.1. Quality Measures

Study personnel will be trained on all important study related aspects, including a trial-specific training, GCP training, training on data entry and handling and study interventions (blood pressure measurement, ECG application, Echocardiographic minimal scanning, retina scanning). During the study, we have planned quality visits and independent data review through an independent trial monitor.

For quality assurance the Ethics Committee or an independent trial monitor may visit the research sites. Direct access to the source data and all study related files is granted on such occasions. All involved parties keep the participant data strictly confidential.

8.2. Data Recording and Source Data

8.2.1. Case Report Forms

All data will be captured online in electronic case report forms (eCRFs) into the MACRO Electronic Data Capture (EDC) tool. CRFs are kept current to reflect subject status at each phase during the course of study. Participants will not be identified in the CRF by name or initials. Participants will be assigned a unique identifier at screening and randomization, and only these identifiers will be used on all study documentation. If the internet is not working, data will be captured on paper forms until electronic upload is possible. All of the information shown in Table 5 will be captured.

Table 5. Data captured:

	Baseline	Follow-up at 4, 8, and 12 weeks	Follow-up at 24 weeks (end of study)
Demographics	Age, sex, living place	Age, living place	Age, living place
Medical history	HIV status, alcohol and smoking habits, previous and current medication, previous cardiovascular events, important comorbidities	current medication, events since enrolment, side effects of drugs	alcohol and smoking habits, current medication, events since enrolment, side effects of drugs
Patient's symptoms	chest pain, dyspnea, cough, weight loss headache, edema, appetite and others	chest pain, dyspnea, cough, weight loss headache, edema, appetite and others	chest pain, dyspnea, cough, weight loss headache, edema, appetite and others
Adherence		pill count, self-reported adherence	pill count, self-reported adherence
Vital signs (exc BP)	Body weight, height, oxygen saturation, pulse	Body weight, height, oxygen saturation, pulse	Body weight, height, oxygen saturation, pulse
BP Measurement	Determination of reference arm by one measurement each, followed by 2 measurements on the reference arm (subgroup with 24h BP and unattended measurement), see specified indications section 4.3.3	3 measurements on the reference arm (week 12: subgroup with 24h BP and unattended measurement), see specified indications section 4.3.3	3 measurements on the reference arm, see specified indications section 4.3.3
General clinical body examination	Clinical findings of lungs, heart, abdomen, edema, jugular veins	Clinical findings of lungs, heart, abdomen, edema, jugular veins	Clinical findings of lungs, heart, abdomen, edema, jugular veins
Blood test	HIV test if not known HIV-positive, full blood picture, creatinine, alanine aminotransferase, random blood glucose	Lab testing according clinical indication	full blood picture, creatinine, alanine aminotransferase, random blood glucose<br>Plasma biobank for additional analyses (e.g. biomarkers such as BNP, lipids, HBA1c and others)

	Plasma biobank for additional analyses (e.g. biomarkers such as BNP, lipids, HBA1c and others)		
Urine tests	Albumin/Creatinine ratio,	Urine testing according clinical indication	Albumin/Creatinine ratio,
Imaging	remote echocardiography(87), electro cardiogram, retinal picture<br><br>chest x-ray on clinical indication only	Imaging according clinical indication	Remote echocardiography, electrocardiogram, retinal picture<br><br>chest x-ray on clinical indication only

8.2.2. Data collection

All clinical data except laboratory and results from ECG, echocardiography, fundoscopy, x-ray, 24h BP measurements will be captured directly in a MACRO database during the participant visit by the study staff using computers or tablets without other source data. The blood pressure measurements are additionally noted on a hardcopy log kept within the patient file. The MACRO study database will be built and managed centrally by the Swiss TPH. Data from laboratory results will be captured within the lab as per laboratory SOPs. Source documents remain within the laboratory. Results are transferred to the clinician by the study lab technician as per local SOP on paper (source data). Results will be entered by the study lab technician (for Ifakara site) and study nurse (for Lesotho site) into MACRO and confirmed by study nurse (for Ifakara site)/ MD (for Lesotho site) and signed. Results of ECG, remote echocardiogram and retinal pictures will be collected directly (ECG on paper, echocardiogram as stored loops, retinal picture as an electronic picture stored in the analyzing device), scanned and uploaded for later evaluation. If x-rays are done, they will be photographed and stored as well electronically. These results will be analyzed at a later time point offline by an experienced cardiologist and ophthalmologist blinded for study arm and type of visit.

8.2.3. Data management

A study specific Data Management Plan will be in place before the start of data collection that describes all data management products and procedures in detail. This includes but is not limited to software details and procedures specific to data collection, management and review. The Data Management Plan has been submitted in mySNF. All investigators will receive training for data capture and management. The Principal Investigator is responsible for data quality and will take reasonable measures to ensure complete and accurate data. Study data will be reviewed regularly by monitors and data managers and queries will be raised within the electronic data capture system to clarify inconsistencies, incoherencies and missing data for the purpose of data cleaning. A computer-generated time stamped audit trail will keep track of all user-specific data processing operations such as creation, modification and deletion.

8.2.4. Data Security and Storage

Data will be kept in compliance with local legal requirements, for a minimum of ten years. All relevant recorded data will be encrypted and password secured, using MACRO electronic database, according to the guidelines of good clinical practice. In each country, a master list linking the participant unique identifier and the participant details such as name will be kept by the site principal investigator in a locked cupboard. All investigators will have access to the data. Swiss TPH servers are located in Basel with a defined policy in place for server set-up, maintenance and security. This includes processes regarding server qualification, back-ups, disaster recovery and restricted server access. Archiving details, such as archival format, retention schedule and secure storage according to GCP guidelines, are agreed upon with sponsor before archival. The study database in MACRO allows for database freeze and database lock for either interim or final statistical analyses. Database lock will prevent any further

changes after final data entry and query resolution and will be implemented according to agreed timelines.

All data will be stored securely in a way that allows future access for the research team. We will establish procedures for approved access to the data as permitted by the informed consent provided. All data will be owned by the sponsor-investigator. A minimal verified and anonymized dataset will be made available to a public data repository. Submission of the primary manuscript will be done within one year of trial completion, and will be published as open access. Authorship of publications will be based on accepted standards as contained in the guideline "Uniform Requirements for Manuscripts Submitted to Biomedical Journals of the International Committee of Medical Journal Editors" (www.icmje.org/urm_main.html). In line with standard data access principles, we will ensure that all relevant data related to the publication will be made available such as through a data repository, and that anonymization and other measures will be taken to protect individual and personally identifiable information in the datasets. The full study protocol will be made available as part of the publication in a scientific journal to enhance transparency, reproducibility, and interpretation of trial results and to support future clinical trials in this field.

8.2.5. Specification of source documents

Source data includes the original documents relating to the study, as well as the medical treatment and medical history of the participant. For data that are entered directly into the MACRO database, the electronic data are the source data. For data such as laboratory results, the source data will be the documentation produced by the laboratory. A paper copy of any such results will be kept in the patient file for this trial. Additional paper documents will be available at the site to document the existence of the study participants.

8.3. Confidentiality and coding

8.3.1. Participant privacy and confidentiality

Trial and participant data will be handled with uttermost discretion and is only accessible to authorized personnel who require the data to fulfill their duties within the scope of the study. On the CRFs and other study specific documents, participants are only identified by a unique participant number. The investigator affirms and upholds the principle of the participant's right to privacy and that they shall comply with applicable privacy laws. Especially, anonymity of the participants will be guaranteed when presenting the data at scientific meetings or publishing them in scientific journals. Individual subject medical information obtained as a result of this study is considered confidential and disclosure to third parties is prohibited. Subject confidentiality will be further ensured by utilizing subject identification code numbers. For data verification purposes, a competent authority or an ethics committee may require direct access to parts of the medical records relevant to the study, including participants' medical history. Identification lists and patients file will be kept at a locked cupboard in a study room, accessible only by study staff. The eCRF will be entered in laptop computers or tablets, which are password protected.

Biological material in this study is not identified by participant name but by a unique participant number. Biological material is appropriately stored in a restricted area only accessible to the authorized personnel. Blood samples stored in freezers for further analysis will be accessible only to study team and lab staff. A log for freezer room entry ensures documentation of access.

8.4 Retention and destruction of study biological material

The time-period of sample storage at the local laboratory will last until finalization of planned laboratory

workup, which will be after a maximum time of 10 years after study closure. After such finalization, all samples will be destroyed according to local regulations.

9. Monitoring and Registration

9.1. Study Registration

The Study will be registered with https://clinicaltrials.gov as soon as ethical approval is obtained

9.2. Study monitoring

9.2.1. Monitoring Institution

Monitoring will be done by the Quality Management team of the Ifakara Health Institute for the Ifakara Site and by the Clinical Operations Unit, Swiss Tropical and Public Health Institute for the Lesotho Site.

The study sites will be visited by the trial monitor team (in Ifakara by Ifakara Health Institute Quality team and in Lesotho by SwissTPH Clinical Operations) for site initiation, during the trial and at study closure according to a separate monitoring plan. In addition to on-site monitoring, central monitoring checks will be put into place. Data quality checks will be built into the MACRO study database that will flag inconsistent and erroneous data. This MACRO Elsevier interface will be done every two months by the trial monitoring unit of the Swiss Tropical and Public Health Institute

9.2.2. Data Safety Monitoring

The safety profile of all involved drugs is well-known, and the interventions do not include any new drugs. Safety outcomes will be assessed by Adverse events/Serious adverse events questionnaires and will be documented systematically at the earliest possible time point. A separate, detailed safety monitoring plan will be developed to handle (Serious) Adverse Events ([S]AE), in-line with Swiss and local ethics regulations. (S)AEs will be separately documented, graded according to the CTC grading and managed according to study sites standard procedure following the national guidelines (Tanzanian and Lesotho Guidelines). The study physicians are responsible for all safety procedures. If a participant develops an AE of Grade 2 or higher at last study visit, he/she will remain under observation by the study physicians even after study termination, until the AE is resolved or stabilized.

10. Funding/Publication/declaration of Interest

The Swiss National Research Foundation has granted 640’273 CHF for this trial. Additional funding will be sought.
An agreement between the different institutions (SNF), the University of Basel, the Ifakara Health Institute and SolidarMed will regulate the contractual responsibilities and duties.

Schedule and milestones (2 years)

Figure 6 shows the planned trial schedule and milestones. Due to the COVID-19 pandemic recruitment was paused temporarily in both sites. Therefore, the trial schedule has been adapted and as shown in Figure 7

Figure 6. Initial Schedule and milestones.

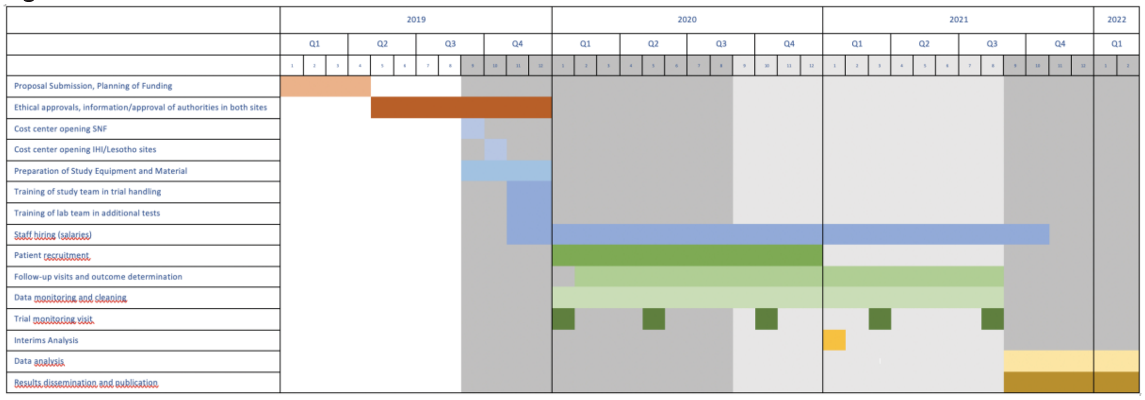

Figure 7. Adapted Schedule and milestones.

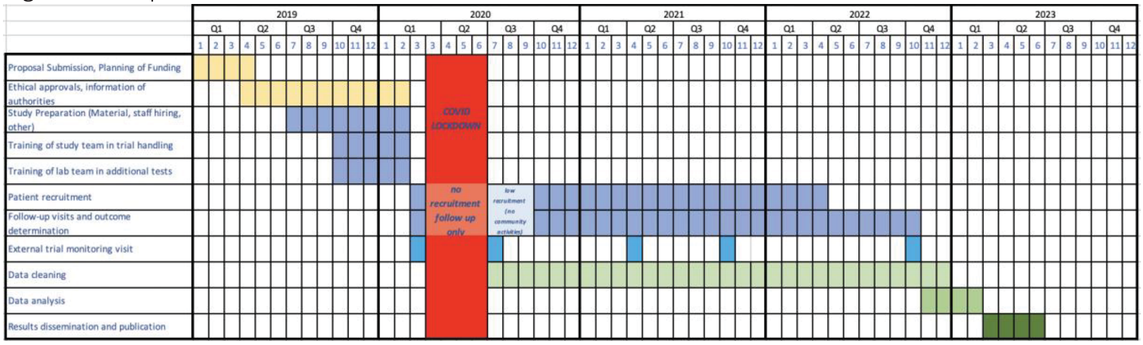

Declaration of interest

No conflict of interest is to be reported

11. Study Administrative Information

11.1. Investigators and Study Team

Name	*Role for Study*	*Position and Affiliation*	*Email*
Maja Weisser Rohacek	*Sponsor-Investigator*	*Staff Physician Division of Infectious Diseases, University Hospital Basel, Basel, Switzerland*<br>*Clinical Research Coordinator, Chronic Diseases Clinic of Ifakara, Ifakara Health Institute*	*m.weisser@unibas.ch*
Jacqueline Nkouabi	*Principal Investigator*	*Researcher and MD of Chronic Diseases Clinic, Ifakara Health Institute, Ifakara, Tanzania,*	*jnkouabi@ihi.or.tz*
Herry Mapesi	*Co-investigator*	*Research Collaborator of Chronic Diseases Clinic, Ifakara Health Institute, Ifakara, Tanzania*	*hmapesi@ihi.or.tz*
Niklaus Labhardt	*Co-Investigator*	*Project leader, Swiss Tropical and Public Health Institute, University of Basel, Basel, Switzerland*	*n.labhardt@unibas.ch*
Thilo Burkard	*Co- Investigator*	*Head of Hypertension Centre, Staff physician, Medical Outpatient Department and Department of Cardiology, University Hospital Basel, Basel, Switzerland*	*Thilo.burkard@usb.ch*
Martin Rohacek	*Co- Investigator*	*Head of Emergency Dpt, St. Francis Referral Hospital; Research Scientist, Ifakara Health Institute, Ifakara, Tanzania*	*mrohacek@ihi.or.tz*
Study site team TANZANIA			
Henrieth Wilson	*Co-Investigator, study physician Ifakara*	*Researcher and MD of Chronic Diseases Clinic, Ifakara Health Institute, Ifakara, Tanzania,*	*hwilson@ihi.or.tz*

Dr. Elizabeth Senkoro	*Co-Investigator, study physician Ifakara*	*Researcher and MD of Chronic Diseases Clinic, Ifakara Health Institute, Ifakara, Tanzania,*	*esenkoro@ihi.or.tz*
Geofrey Mbunda	*Study nurse*	*Site study nurse of the CDCI/IHI*	*gmbunda@ihi.or.tz*
Jamali Bashiri	*Study nurse*	*Site study nurse of the CDCI/IHI*	*jbashiri@ihi.or.tz*
Theonestina Byakuzana	*Study Lab technician*	*Site study lab technician of the CDCI/IHI*	*tbyakuzana@ihi.or.tz*
Study site team LESOTHO			
Ravi Shankar Gupta	*Co-Investigator Lesotho*	*Project Manager, Health First Lesotho SolidarMed*	*R.Gupta@solidarmed.ch*
Alain Amstutz	*Collaborator (co-site PI Lesotho)*	*PhD student Clinical Research, Swiss Tropical and Public Health Institute, University Hospital Basel, University of Basel*	*Alain.amstutz@unibas.ch*
Blaise Dilumbu Lukau	*Co-investigator, Study physician, Mokhotlong*	*Researcher and MD of Solidarmed Lesotho, Mokhotlong Hospital, Mokhotlong, Lesotho*	*lukaublaise@yahoo.fr*
Ayesha Tarr	*Study nurse*	*Site study nurse of Solidarmed Lesotho*	shashutarr@gmail.com
Elsie Ramapepe.	*Study nurse assistant*	*Site study nurse assistant of Solidarmed Lesotho*	elsiemotsoaole@gmail.com
Thato Maqhama	*Study Lab technician*	*Site study lab technician of Mokhotlong Government Hospital*	tmaqhama@gmail.com
Other Collaborators			
Winfried Gingo	*Collaborator*	*Hospital Director, St. Francis Referral Hospital*	*wgingo@yahoo.com*
Josephine Muhairwe	*Collaborator*	*Country Director SolidarMed Lesotho*	*J.Muhairwe@solidarmed.ch*
Fiona Vanobberghen	*Co-Investigator Project Statistician*	*Statistician at the Swiss Tropical and Public Health Institute, University of Basel, Basel, Switzerland*	*Fiona.vanobberghen@swisstph.ch*
Tracy Glass	*Co-Investigator (supervising statistician)*	*Lead Statistician of KIULARCO at the Swiss Tropical and Public Health Institute, University of Basel, Basel, Switzerland*	*Tracy.glass@swisstph.ch*
Moniek Bresser	*Co-investigator (data manager)*	*Scientific Collaborator, Swiss Tropical and public Health Institute, University of Basel, Switzerland*	*Moniek.bresser@swisstph.ch*
Mark Lambiris	*Co-Investigator (cost-effective-ness analysis*	*Postdoc Scientific Collaborator, Swiss Tropical and Public Health Institute, University of Basel, Basel, Switzerland*	Mark.lambiris@swisstph.ch
Günther Fink	*Collaborator (cost-effective-ness analysis*	*Head of Household Economics and Health Systems, Swiss Tropical and Public Health Institute, University of Basel, Basel, Switzerland*	*Guenther.fink@swisstph.ch*
Jarmila Hanekova	*Study monitor*	*Clinical research associate for monitoring, Swiss Tropical and Public Health Institute, University of Basel, Basel, Switzerland*	*Jarmila.Hanekova@swisstph.ch*
Sonja Bernhard	*Study Monitor*	*Group leader of monitoring, Swiss Tropical and Public Health Institute, University of Basel, Basel, Switzerland*	*Sonja.Bernhard@swisstph.ch*
Astrid Mattes	*Quality responsible*	*Head of Quality Management, Department of Medicine Swiss Tropical and Public Health Institute, University of Basel, Basel, Switzerland*	*Astrid.Mattes@swisstph.ch*
Elisabeth Reus	*Quality responsible*	*Head of Clinial Operation Unit, Swiss Tropical and Public Health Institute, University of Basel, Basel, Switzerland*	*Elisabeth.Reus@swisstph.ch*
Manuel Battegay	*Collaborator*	*Head of the Division of Infectious Diseases, University Hospital Basel, Basel, Switzerland*	*Manuel.batttegay@usb.ch*
Daniel H. Paris	*Collaborator*	*Head of Department of Medicine, Swiss Tropical and Public Health Institute, University of Basel, Basel, Switzerland*	*Daniel.paris@swisstph.ch*

12. Bibliography

1. UNAIDS Data 2018. 2018.
2. Forouzanfar MH, Liu P, Roth GA, Ng M, Biryukov S, Marczak L, et al. Global Burden of Hypertension and Systolic Blood Pressure of at Least 110 to 115 mm Hg, 1990-2015. JAMA. 2017;317(2):165-82.
3. Mokdad AH, Forouzanfar MH, Daoud F, Mokdad AA, El Bcheraoui C, Moradi-Lakeh M, et al. Global burden of diseases, injuries, and risk factors for young people's health during 1990-2013: a systematic analysis for the Global Burden of Disease Study 2013. Lancet. 2016;387(10036):2383-401.
4. Flack JM, Sica DA, Bakris G, Brown AL, Ferdinand KC, Grimm RH, Jr., et al. Management of high blood pressure in Blacks: an update of the International Society on Hypertension in Blacks consensus statement. Hypertension. 2010;56(5):780-800.
5. Organization WH. Raised blood pressure: Situations and Trends 2018 [Available from: http://www.who.int/gho/ncd/risk_factors/blood_pressure.

6. Perkovic V, Huxley R, Wu Y, Prabhakaran D, MacMahon S. The burden of blood pressure-related disease: a neglected priority for global health. Hypertension. 2007;50(6):991-7.
7. Reddy KS, Yusuf S. Emerging epidemic of cardiovascular disease in developing countries. Circulation. 1998;97(6):596-601.
8. Addo J, Smeeth L, Leon DA. Hypertension in sub-saharan Africa: a systematic review. Hypertension. 2007;50(6):1012-8.
9. Kearney PM, Whelton M, Reynolds K, Muntner P, Whelton PK, He J. Global burden of hypertension: analysis of worldwide data. Lancet. 2005;365(9455):217-23.
10. Opie LH, Seedat YK. Hypertension in sub-Saharan African populations. Circulation. 2005;112(23):3562-8.
11. Ataklte F, Erqou S, Kaptoge S, Taye B, Echouffo-Tcheugui JB, Kengne AP. Burden of undiagnosed hypertension in sub-saharan Africa: a systematic review and meta-analysis. Hypertension. 2015;65(2):291-8.
12. Adeyemo A, Gerry N, Chen G, Herbert A, Doumatey A, Huang H, et al. A genome-wide association study of hypertension and blood pressure in African Americans. PLoS Genet. 2009;5(7):e1000564.
13. Young JH, Chang YP, Kim JD, Chretien JP, Klag MJ, Levine MA, et al. Differential susceptibility to hypertension is due to selection during the out-of-Africa expansion. PLoS Genet. 2005;1(6):e82.
14. Tiffin N, Meintjes A, Ramesar R, Bajic VB, Rayner B. Computational analysis of candidate disease genes and variants for salt-sensitive hypertension in indigenous Southern Africans. PLoS One. 2010;5(9):e12989.
15. Falkner B, Kushner H. Effect of chronic sodium loading on cardiovascular response in young blacks and whites. Hypertension. 1990;15(1):36-43.
16. Howard G, Lackland DT, Kleindorfer DO, Kissela BM, Moy CS, Judd SE, et al. Racial differences in the impact of elevated systolic blood pressure on stroke risk. JAMA Intern Med. 2013;173(1):46-51.
17. Assembly. S-sWH. Follow-up to the Political Declaration of the high-level meeting of the General Assembly on the prevention and control of non-communicable diseases (Resolution WHA66/10). 2013 [Available from: http://apps.who.int/gb/ebwha/pdf.
18. Yoruk A, Boulos PK, Bisognano JD. The State of Hypertension in Sub-Saharan Africa: Review and Commentary. Am J Hypertens. 2018;31(4):387-8.
19. Obel N, Thomsen HF, Kronborg G, Larsen CS, Hildebrandt PR, Sorensen HT, et al. Ischemic heart disease in HIV-infected and HIV-uninfected individuals: a population-based cohort study. Clin Infect Dis. 2007;44(12):1625-31.
20. McDonald CL, Kaltman JR. Cardiovascular disease in adult and pediatric HIV/AIDS. J Am Coll Cardiol. 2009;54(13):1185-8.
21. Islam FM, Wu J, Jansson J, Wilson DP. Relative risk of cardiovascular disease among people living with HIV: a systematic review and meta-analysis. HIV Med. 2012;13(8):453-68.
22. Data Collection on Adverse Events of Anti HIVdSG, Smith C, Sabin CA, Lundgren JD, Thiebaut R, Weber R, et al. Factors associated with specific causes of death amongst HIV-positive individuals in the D:A:D Study. AIDS. 2010;24(10):1537-48.
23. Schouten J, Wit FW, Stolte IG, Kootstra NA, van der Valk M, Geerlings SE, et al. Cross-sectional comparison of the prevalence of age-associated comorbidities and their risk factors between HIV-infected and uninfected individuals: the AGEhIV cohort study. Clin Infect Dis. 2014;59(12):1787-97.
24. Mattana J, Siegal FP, Sankaran RT, Singhal PC. Absence of age-related increase in systolic blood pressure in ambulatory patients with HIV infection. Am J Med Sci. 1999;317(4):232-7.
25. Bergersen BM, Sandvik L, Dunlop O, Birkeland K, Bruun JN. Prevalence of hypertension in HIV-positive patients on highly active retroviral therapy (HAART) compared with HAART-naive and HIV-negative controls: results from a Norwegian study of 721 patients. Eur J Clin Microbiol Infect Dis. 2003;22(12):731-6.
26. Piepoli MF, Hoes AW, Agewall S, Albus C, Brotons C, Catapano AL, et al. 2016 European Guidelines on cardiovascular disease prevention in clinical practice: The Sixth Joint Task Force of the European Society of Cardiology and Other Societies on Cardiovascular Disease Prevention in Clinical Practice (constituted by representatives of 10 societies and by invited experts)Developed with the special contribution of the European Association for Cardiovascular Prevention & Rehabilitation (EACPR). Eur Heart J. 2016;37(29):2315-81.
27. Seaberg EC, Munoz A, Lu M, Detels R, Margolick JB, Riddler SA, et al. Association between highly active antiretroviral therapy and hypertension in a large cohort of men followed from 1984 to 2003. AIDS. 2005;19(9):953-60.
28. Thiebaut R, El-Sadr WM, Friis-Moller N, Rickenbach M, Reiss P, Monforte AD, et al. Predictors of hypertension and changes of blood pressure in HIV-infected patients. Antivir Ther. 2005;10(7):811-23.
29. Palacios R, Santos J, Garcia A, Castells E, Gonzalez M, Ruiz J, et al. Impact of highly active antiretroviral therapy on blood pressure in HIV-infected patients. A prospective study in a cohort of naive patients. HIV Med. 2006;7(1):10-5.
30. Jerico C, Knobel H, Montero M, Sorli ML, Guelar A, Gimeno JL, et al. Hypertension in HIV-infected patients: prevalence and related factors. Am J Hypertens. 2005;18(11):1396-401.
31. Crane HM, Van Rompaey SE, Kitahata MM. Antiretroviral medications associated with elevated blood pressure among patients receiving highly active antiretroviral therapy. AIDS. 2006;20(7):1019-26.
32. Ogunmola OJ, Oladosu OY, Olamoyegun AM. Association of hypertension and obesity with HIV and antiretroviral therapy in a rural tertiary health center in Nigeria: a cross-sectional cohort study. Vasc Health Risk Manag. 2014;10:129-37.
33. Hatleberg CI, Ryom L, d'Arminio Monforte A, Fontas E, Reiss P, Kirk O, et al. Association between exposure to antiretroviral drugs and the incidence of hypertension in HIV-positive persons: the Data Collection on Adverse Events of Anti-HIV Drugs (D:A:D) study. HIV Med. 2018.
34. Peyriere H, Eiden C, Macia JC, Reynes J. Antihypertensive drugs in patients treated with antiretrovirals. Ann Pharmacother. 2012;46(5):703-9.
35. Elmer PJ, Obarzanek E, Vollmer WM, Simons-Morton D, Stevens VJ, Young DR, et al. Effects of comprehensive lifestyle modification on diet, weight, physical fitness, and blood pressure control: 18-month results of a randomized trial. Ann Intern Med. 2006;144(7):485-95.
36. Whelton PK, Carey RM, Aronow WS, Casey DE, Jr., Collins KJ, Dennison Himmelfarb C, et al. 2017 ACC/AHA/AAPA/ABC/ACPM/AGS/APhA/ASH/ASPC/NMA/PCNA Guideline for the Prevention, Detection, Evaluation, and Management of High Blood Pressure in Adults: A Report of the American College of Cardiology/American Heart Association Task Force on Clinical Practice Guidelines. Hypertension. 2018;71(6):e13-e115.
37. Hebert PR, Moser M, Mayer J, Glynn RJ, Hennekens CH. Recent evidence on drug therapy of mild to moderate hypertension and decreased risk of coronary heart disease. Arch Intern Med. 1993;153(5):578-81.
38. Law MR, Morris JK, Wald NJ. Use of blood pressure lowering drugs in the prevention of cardiovascular disease: meta-analysis of 147 randomised trials in the context of expectations from prospective epidemiological studies. BMJ. 2009;338:b1665.

39. Rosendorff C, Black HR, Cannon CP, Gersh BJ, Gore J, Izzo JL, Jr., et al. Treatment of hypertension in the prevention and management of ischemic heart disease: a scientific statement from the American Heart Association Council for High Blood Pressure Research and the Councils on Clinical Cardiology and Epidemiology and Prevention. Circulation. 2007;115(21):2761-88.
40. Blood Pressure Lowering Treatment Trialists C, Turnbull F, Neal B, Ninomiya T, Algert C, Arima H, et al. Effects of different regimens to lower blood pressure on major cardiovascular events in older and younger adults: meta-analysis of randomised trials. BMJ. 2008;336(7653):1121-3.
41. Reboussin DM, Allen NB, Griswold ME, Guallar E, Hong Y, Lackland DT, et al. Systematic Review for the 2017 ACC/AHA/AAPA/ABC/ACPM/AGS/APhA/ASH/ASPC/NMA/PCNA Guideline for the Prevention, Detection, Evaluation, and Management of High Blood Pressure in Adults: A Report of the American College of Cardiology/American Heart Association Task Force on Clinical Practice Guidelines. Hypertension. 2018;71(6):e116-e35.
42. Wright JT, Jr., Dunn JK, Cutler JA, Davis BR, Cushman WC, Ford CE, et al. Outcomes in hypertensive black and nonblack patients treated with chlorthalidone, amlodipine, and lisinopril. JAMA. 2005;293(13):1595-608.
43. Li EC, Heran BS, Wright JM. Angiotensin converting enzyme (ACE) inhibitors versus angiotensin receptor blockers for primary hypertension. Cochrane Database Syst Rev. 2014(8):CD009096.
44. Organization WH. Prevention of cardiovascular disease : guidelines for assessment and management of total cardiovascular risk. 2007.
45. Wright JM, Musini VM, Gill R. First-line drugs for hypertension. Cochrane Database Syst Rev. 2018;4:CD001841.
46. Wald DS, Law M, Morris JK, Bestwick JP, Wald NJ. Combination therapy versus monotherapy in reducing blood pressure: meta-analysis on 11,000 participants from 42 trials. Am J Med. 2009;122(3):290-300.
47. Chow CK, Thakkar J, Bennett A, Hillis G, Burke M, Usherwood T, et al. Quarter-dose quadruple combination therapy for initial treatment of hypertension: placebo-controlled, crossover, randomised trial and systematic review. Lancet. 2017;389(10073):1035-42.
48. Williams B, Mancia G, Spiering W, Agabiti Rosei E, Azizi M, Burnier M, et al. 2018 ESC/ESH Guidelines for the management of arterial hypertension. Eur Heart J. 2018.
49. Peck RN, Smart LR, Beier R, Liwa AC, Grosskurth H, Fitzgerald DW, et al. Difference in blood pressure response to ACE-Inhibitor monotherapy between black and white adults with arterial hypertension: a meta-analysis of 13 clinical trials. BMC Nephrol. 2013;14:201.
50. Weir MR, Gray JM, Paster R, Saunders E. Differing mechanisms of action of angiotensin-converting enzyme inhibition in black and white hypertensive patients. The Trandolapril Multicenter Study Group. Hypertension. 1995;26(1):124-30.
51. Twagirumukiza M, Van Bortel LM. Management of hypertension at the community level in sub-Saharan Africa (SSA): towards a rational use of available resources. J Hum Hypertens. 2011;25(1):47-56.
52. Whitworth JA, World Health Organization ISoHWG. 2003 World Health Organization (WHO)/International Society of Hypertension (ISH) statement on management of hypertension. J Hypertens. 2003;21(11):1983-92.
53. Brewster LM, van Montfrans GA, Kleijnen J. Systematic review: antihypertensive drug therapy in black patients. Ann Intern Med. 2004;141(8):614-27.
54. Seedat YK. Trial of atenolol and chlorthalidone for hypertension in black South Africans. Br Med J. 1980;281(6250):1241-3.
55. Sareli P, Radevski IV, Valtchanova ZP, Libhaber E, Candy GP, Den Hond E, et al. Efficacy of different drug classes used to initiate antihypertensive treatment in black subjects: results of a randomized trial in Johannesburg, South Africa. Arch Intern Med. 2001;161(7):965-71.
56. M'Buyamba-Kabangu JR, Anisiuba BC, Ndiaye MB, Lemogoum D, Jacobs L, Ijoma CK, et al. Efficacy of newer versus older antihypertensive drugs in black patients living in sub-Saharan Africa. J Hum Hypertens. 2013;27(12):729-35.
57. Odili AN, Ezeala-Adikaibe B, Ndiaye MB, Anisiuba BC, Kamdem MM, Ijoma CK, et al. Progress report on the first sub-Saharan Africa trial of newer versus older antihypertensive drugs in native black patients. Trials. 2012;13:59.
58. Djoumessi RN, Noubiap JJ, Kaze FF, Essouma M, Menanga AP, Kengne AP, et al. Effect of low-dose spironolactone on resistant hypertension in type 2 diabetes mellitus: a randomized controlled trial in a sub-Saharan African population. BMC Res Notes. 2016;9:187.
59. Iyalomhe GB, Omogbai EK, Isah AO, Iyalomhe OO, Dada FL, Iyalomhe SI. Efficacy of initiating therapy with amlodipine and hydrochlorothiazide or their combination in hypertensive Nigerians. Clin Exp Hypertens. 2013;35(8):620-7.
60. Ojji DB, Mayosi B, Francis V, Badri M, Cornelius V, Smythe W, et al. Comparison of Dual Therapies for Lowering Blood Pressure in Black Africans. N Engl J Med. 2019.
61. Vanobberghen F, Letang E, Gamell A, Mnzava DK, Faini D, Luwanda LB, et al. A decade of HIV care in rural Tanzania: Trends in clinical outcomes and impact of clinic optimisation in an open, prospective cohort. PLoS One. 2017;12(7):e0180983.
62. Letang E, Kalinjuma AV, Glass TR, Gamell A, Mapesi H, Sikalengo GR, et al. Cohort profile: The Kilombero and Ulanga Antiretroviral Cohort (KIULARCO) - A prospective HIV cohort in rural Tanzania. Swiss Med Wkly. 2017;147:w14485.
63. Ntamatungiro AJ, Muri L, Glass TR, Erb S, Battegay M, Furrer H, et al. Strengthening HIV therapy and care in rural Tanzania affects rates of viral suppression. J Antimicrob Chemother. 2017;72(7):2069-74.
64. Rodriguez-Arboli E, Mwamelo K, Kalinjuma AV, Furrer H, Hatz C, Tanner M, et al. Incidence and risk factors for hypertension among HIV patients in rural Tanzania - A prospective cohort study. PLoS One. 2017;12(3):e0172089.
65. Mapesi H, Kalinjuma AV, Ngerecha A, Franzeck F, Hatz C, Tanner M, et al. Prevalence and Evolution of Renal Impairment in People Living With HIV in Rural Tanzania. Open Forum Infect Dis. 2018;5(4):ofy072.
66. Labhardt ND, Ringera I, Lejone TI, Klimkait T, Muhairwe J, Amstutz A, et al. Effect of Offering Same-Day ART vs Usual Health Facility Referral During Home-Based HIV Testing on Linkage to Care and Viral Suppression Among Adults With HIV in Lesotho: The CASCADE Randomized Clinical Trial. JAMA. 2018;319(11):1103-12.
67. Labhardt ND, Motlomelo M, Cerutti B, Pfeiffer K, Kamele M, Hobbins MA, et al. Home-based versus mobile clinic HIV testing and counseling in rural Lesotho: a cluster-randomized trial. PLoS Med. 2014;11(12):e1001768.
68. Cerutti B, Broers B, Masetsibi M, Faturiyele O, Toti-Mokoteli L, Motlatsi M, et al. Alcohol use and depression: link with adherence and viral suppression in adult patients on antiretroviral therapy in rural Lesotho, Southern Africa: a cross-sectional study. BMC Public Health. 2016;16:947.
69. Labhardt ND, Muller UF, Ringera I, Ehmer J, Motlatsi MM, Pfeiffer K, et al. Metabolic syndrome in patients on first-line antiretroviral therapy containing zidovudine or tenofovir in rural Lesotho, Southern Africa. Trop Med Int Health. 2017;22(6):725-33.

70. Cerutti Bernard AA, Ringera Isaac, Thin Kyaw, Glass Tracy, Labhardt Niklaus D. . Comparison of Lipid Profile and Glycosylated Hemoglobin Levels among HIV-infected and Non-HIV-Infected Individuals in Lesotho: A Community- based Cross-sectional Study 2017.
71. STG L. Standard Treatment Guidelines (STG) for Lesotho. In: Ministery of Health L, editor. 2017.
72. Pool JL, Glazer R, Weinberger M, Alvarado R, Huang J, Graff A. Comparison of valsartan/hydrochlorothiazide combination therapy at doses up to 320/25 mg versus monotherapy: a double-blind, placebo-controlled study followed by long-term combination therapy in hypertensive adults. Clin Ther. 2007;29(1):61-73.
73. Materson BJ, Reda DJ, Cushman WC, Massie BM, Freis ED, Kochar MS, et al. Single-drug therapy for hypertension in men. A comparison of six antihypertensive agents with placebo. The Department of Veterans Affairs Cooperative Study Group on Antihypertensive Agents. N Engl J Med. 1993;328(13):914-21.
74. Middlemost SJ, Tager R, Davis J, Sareli P. Effectiveness of enalapril in combination with low-dose hydrochlorothiazide versus enalapril alone for mild to moderate systemic hypertension in black patients. Am J Cardiol. 1994;73(15):1092-7.
75. Weir MR, Ferdinand KC, Flack JM, Jamerson KA, Daley W, Zelenkofske S. A noninferiority comparison of valsartan/hydrochlorothiazide combination versus amlodipine in black hypertensives. Hypertension. 2005;46(3):508-13.
76. Agodoa LY, Appel L, Bakris GL, Beck G, Bourgoignie J, Briggs JP, et al. Effect of ramipril vs amlodipine on renal outcomes in hypertensive nephrosclerosis: a randomized controlled trial. JAMA. 2001;285(21):2719-28.
77. Fogo A, Breyer JA, Smith MC, Cleveland WH, Agodoa L, Kirk KA, et al. Accuracy of the diagnosis of hypertensive nephrosclerosis in African Americans: a report from the African American Study of Kidney Disease (AASK) Trial. AASK Pilot Study Investigators. Kidney Int. 1997;51(1):244-52.
78. Stanifer JW, Jing B, Tolan S, Helmke N, Mukerjee R, Naicker S, et al. The epidemiology of chronic kidney disease in sub-Saharan Africa: a systematic review and meta-analysis. Lancet Glob Health. 2014;2(3):e174-81.
79. Brown NJ, Ray WA, Snowden M, Griffin MR. Black Americans have an increased rate of angiotensin converting enzyme inhibitor-associated angioedema. Clin Pharmacol Ther. 1996;60(1):8-13.
80. Taddei S. Combination therapy in hypertension: what are the best options according to clinical pharmacology principles and controlled clinical trial evidence? Am J Cardiovasc Drugs. 2015;15(3):185-94.
81. Law MR, Wald NJ, Morris JK, Jordan RE. Value of low dose combination treatment with blood pressure lowering drugs: analysis of 354 randomised trials. BMJ. 2003;326(7404):1427.
82. Xu W, Goldberg SI, Shubina M, Turchin A. Optimal systolic blood pressure target, time to intensification, and time to follow-up in treatment of hypertension: population based retrospective cohort study. BMJ. 2015;350:h158.
83. Carlberg B, Samuelsson O, Lindholm LH. Atenolol in hypertension: is it a wise choice? Lancet. 2004;364(9446):1684-9.
84. Messerli FH, Bangalore S, Julius S. Risk/benefit assessment of beta-blockers and diuretics precludes their use for first-line therapy in hypertension. Circulation. 2008;117(20):2706-15; discussion 15.
85. Jain A, Tandri H, Dalal D, Chahal H, Soliman EZ, Prineas RJ, et al. Diagnostic and prognostic utility of electrocardiography for left ventricular hypertrophy defined by magnetic resonance imaging in relationship to ethnicity: the Multi-Ethnic Study of Atherosclerosis (MESA). Am Heart J. 2010;159(4):652-8.
86. Sokolow M, Lyon TP. The ventricular complex in left ventricular hypertrophy as obtained by unipolar precordial and limb leads. 1949. Ann Noninvasive Electrocardiol. 2001;6(4):343-68.
87. Nascimento BR, Beaton AZ, Nunes MCP, Tompsett AR, Oliveira KKB, Diamantino AC, et al. Integration of echocardiographic screening by non-physicians with remote reading in primary care. Heart. 2018.
88. Cuspidi C, Rescaldani M, Sala C. Prevalence of echocardiographic left-atrial enlargement in hypertension: a systematic review of recent clinical studies. Am J Hypertens. 2013;26(4):456-64.
89. Su G, Cao H, Xu S, Lu Y, Shuai X, Sun Y, et al. Left atrial enlargement in the early stage of hypertensive heart disease: a common but ignored condition. J Clin Hypertens (Greenwich). 2014;16(3):192-7.
90. Maamari RN, Keenan JD, Fletcher DA, Margolis TP. A mobile phone-based retinal camera for portable wide field imaging. Br J Ophthalmol. 2014;98(4):438-41.
91. McHomvu E, Mbunda G, Simon N, Kitila F, Temba Y, Msumba I, et al. Diagnoses made in an Emergency Department in rural sub-Saharan Africa. Swiss Med Wkly. 2019;149:w20018.
92. Ministry of Health of Lesotho aWHO. Lesotho STEPS Survey 2012 (WHO STEPS chronic disease risk factor surveillance). . 2012.
93. Noubiap JJ, Nansseu JR, Nkeck JR, Nyaga UF, Bigna JJ. Prevalence of white coat and masked hypertension in Africa: A systematic review and meta-analysis. J Clin Hypertens (Greenwich). 2018.
94. Appel LJ, Wright JT, Jr., Greene T, Agodoa LY, Astor BC, Bakris GL, et al. Intensive blood-pressure control in hypertensive chronic kidney disease. N Engl J Med. 2010;363(10):918-29.
95. Williams B, MacDonald TM, Morant S, Webb DJ, Sever P, McInnes G, et al. Spironolactone versus placebo, bisoprolol, and doxazosin to determine the optimal treatment for drug-resistant hypertension (PATHWAY-2): a randomised, double-blind, crossover trial. Lancet. 2015;386(10008):2059-68.
96. Beaney T, Burrell LM, Castillo RR, Charchar FJ, Cro S, Damasceno A, et al. May Measurement Month 2018: a pragmatic global screening campaign to raise awareness of blood pressure by the International Society of Hypertension. Eur Heart J. 2019.
97. Belghazi J, El Feghali RN, Moussalem T, Rejdych M, Asmar RG. Validation of four automatic devices for self-measurement of blood pressure according to the International Protocol of the European Society of Hypertension. Vasc Health Risk Manag. 2007;3(4):389-400.
98. O'Brien E, Parati G, Stergiou G, Asmar R, Beilin L, Bilo G, et al. European Society of Hypertension position paper on ambulatory blood pressure monitoring. J Hypertens. 2013;31(9):1731-68.
99. Myers MG, Valdivieso M, Chessman M, Kiss A. Can sphygmomanometers designed for self-measurement of blood pressure in the home be used in office practice? Blood Press Monit. 2010;15(6):300-4.
100. Group SR, Wright JT, Jr., Williamson JD, Whelton PK, Snyder JK, Sink KM, et al. A Randomized Trial of Intensive versus Standard Blood-Pressure Control. N Engl J Med. 2015;373(22):2103-16.
101. Kjeldsen SE, Mancia G. The Un-Observed Automated Office Blood Pressure Measurement Technique Used in the SPRINT Study Points to a Standard Target Office Systolic Blood Pressure <140 mmHg. Curr Hypertens Rep. 2017;19(1):3.
102. Schiffrin EL, Calhoun DA, Flack JM. SPRINT Proves that Lower Is Better for Nondiabetic High-Risk Patients, but at a Price. Am J Hypertens. 2016;29(1):2-4.

103. Jamerson K, Weber MA, Bakris GL, Dahlof B, Pitt B, Shi V, et al. Benazepril plus amlodipine or hydrochlorothiazide for hypertension in high-risk patients. N Engl J Med. 2008;359(23):2417-28.
104. Schulz KF, Altman DG, Moher D, Group C. CONSORT 2010 Statement: updated guidelines for reporting parallel group randomised trials. Trials. 2010;11:32.
105. Juszczak E, Altman DG, Hopewell S, Schulz K. Reporting of Multi-Arm Parallel-Group Randomized Trials: Extension of the CONSORT 2010 Statement. JAMA. 2019;321(16):1610-20.
106. Piaggio G, Elbourne DR, Pocock SJ, Evans SJ, Altman DG, Group C. Reporting of noninferiority and equivalence randomized trials: extension of the CONSORT 2010 statement. JAMA. 2012;308(24):2594-604.
107. Pocock SJ, Assmann SE, Enos LE, Kasten LE. Subgroup analysis, covariate adjustment and baseline comparisons in clinical trial reporting: current practice and problems. Stat Med. 2002;21(19):2917-30.
108. Kahan BC, Morris TP. Reporting and analysis of trials using stratified randomisation in leading medical journals: review and reanalysis. BMJ. 2012;345:e5840.
109. Committee for Proprietary Medicinal P. Points to consider on switching between superiority and non-inferiority. Br J Clin Pharmacol. 2001;52(3):223-8.
110. Sanders GD, Neumann PJ, Basu A, Brock DW, Feeny D, Krahn M, et al. Recommendations for Conduct, Methodological Practices, and Reporting of Cost-effectiveness Analyses: Second Panel on Cost-Effectiveness in Health and Medicine. Jama. 2016;316(10):1093-103.
111. World Health Organization. Making choices in health: WHO guide to cost-effectiveness analysis. Geneva, Switzerland: World Health Organization,; 2003.
112. Salomon JA, Haagsma JA, Davis A, de Noordhout CM, Polinder S, Havelaar AH, et al. Disability weights for the Global Burden of Disease 2013 study. The Lancet Global health. 2015;3(11):e712-23.
113. Smit M, Olney J, Ford NP, Vitoria M, Gregson S, Vassall A, et al. The growing burden of noncommunicable disease among persons living with HIV in Zimbabwe. AIDS. 2018;32(6):773-82.
114. Kruk ME, Gage AD, Arsenault C, Jordan K, Leslie HH, Roder-DeWan S, et al. High-quality health systems in the Sustainable Development Goals era: time for a revolution. Lancet Glob Health. 2018.
