# Supplementary material for: Treatment Strategies to Control Blood Pressure in People With Hypertension in Tanzania and Lesotho: A Randomized Clinical Trial
Source: JAMA Cardiol. 2025 Jan 29;10(4):321–33. doi: 10.1001/jamacardio.2024.5124 (PMC11780507; doi:10.1001/jamacardio.2024.5124)
Supplement: Supplement 2. — Statistical Analysis Plan. [file jamacardiol-e245124-s002.pdf]

DocuSign Envelope ID: 880810C0-2986-4529-AFF3-3AB6B2CC01E3

COARTHA: identifying most effective treatment strategies to control arterial hypertension in sub-Saharan Africa – a randomized controlled trial

STATISTICAL ANALYSIS PLAN

Trial registration: NCT04129840

Protocol version 1.0, 19 May 2022

SAP version 1.0, 30 January 2023

(Statistical analysis plan drafted following Gamble et al (1))

1. Administrative information

Revision history

Version	Date	Who	Comments
0.1	-	Fiona Vanobberghen	Summary in the protocol
0.2	28 June 2022	Fiona Vanobberghen	First draft
0.3	24 October 2022	Fiona Vanobberghen	Following feedback from Maja Weisser, Herry Mapesi and Thilo Burkard; updates further to new version 0.8 of the protocol
0.4	8 November 2022	Fiona Vanobberghen	Following verbal feedback from Maja Weisser and Herry Mapesi; updated to protocol version 1.0; added Tracy Glass in Roles and responsibilities table; removed Basel location for Swiss TPH
0.5	3 January 2023	Fiona Vanobberghen	Added estimands; incorporated feedback from Tracy Glass
1.0	23 January 2023	Fiona Vanobberghen	Incorporated further feedback from Maja Weisser and Tracy Glass, particularly with respect to estimands, missing data, and ACR data

Roles and responsibilities

Name	Affiliation	Role
Fiona Vanobberghen	Swiss TPH, Switzerland	Trial statistician
Tracy Glass	Swiss TPH, Switzerland	Supervising statistician
Maja Weisser	USB, Switzerland<br>Swiss TPH, Switzerland	Sponsor-investigator
Herry Mapesi	IHI, Tanzania<br>SwissTPH, Switzerland	Principal investigator and Ifakara site principal investigator
Herieth Wilson	IHI, Tanzania	Ifakara site principal investigator until 08/22
Jacqueline Nkouabi	IHI Tanzania	Ifakara site principal investigator from 09/22
Ravi Gupta	SolidarMed Lesotho	Mokhotlong site principal investigator

Signatures

Name	Signature	Date
Fiona Vanobberghen	DocuSigned by: Fiona Vanobberghen 99EE806461C740F...	30-Jan-2023 | 15:13:43 CET
Maja Weisser	DocuSigned by: M. Weisser 6F9ADEF4B824455...	30-Jan-2023 | 15:25:57 MEZ

DocuSign Envelope ID: 880810C0-2986-4529-AFF3-3AB6B2CC01E3

Abbreviations

ACR	Albumin creatinine ratio
AE	Adverse event
AUDIT	Alcohol Use Disorders Identification Test
BMI	Body mass index
BP	Blood pressure
CCB	Calcium channel blocker
CI	Confidence interval
CKD-EPI	Chronic Kidney Disease Epidemiology Collaboration
CRF	Case report form
CTCAE	Common Terminology Criteria for Adverse Events
CV	Cardiovascular
DBP	Diastolic blood pressure
ECG	Electrocardiography
eGFR	Estimated glomerular filtration rate
HR	Hazard ratio
LLN	Lower limit of normal
LTFU	Lost to follow up
OR	Odds ratio
SAE	Serious adverse event
SBP	Systolic blood pressure
Swiss TPH	Swiss Tropical and Public Health Institute
TZD	Thiazide diuretic
ULN	Upper limit of normal
UTI	Urinary tract infection
WHO	World Health Organization

Data management and sharing

Data are captured by study staff directly into an electronic MACRO database, hosted at Swiss TPH. Data export is done regularly by data management staff at Swiss TPH. Queries are raised by the data management and statistics team and resolved by the site team.

Key trial data will be made available just before publication of the main manuscript through an appropriate data repository such as Zenodo, and will be referenced accordingly in the main manuscript.

DocuSign Envelope ID: 880810C0-2986-4529-AFF3-3AB6B2CC01E3

Contents

1. Administrative information ... 2
Revision history ... 2
Roles and responsibilities ... 2
Signatures ... 2
Abbreviations ... 3
Data management and sharing ... 3
2. Introduction ... 6
2.1 Background and rationale ... 6
2.2 Objectives ... 6
3. Study methods ... 6
3.1 Trial design ... 6
Trial population ... 6
Trial procedures ... 7
Trial arms ... 7
Table 1. Trial arms. ... 7
Outcomes ... 7
3.2 Randomisation ... 9
3.3 Sample size ... 9
3.4 Framework ... 9
3.5 Statistical interim analyses and stopping guidance ... 9
3.6 Timing of final analysis ... 9
3.7 Timing of outcome assessments ... 9
Table 2. Nominal visits and permitted windows. ... 10
4. Statistical principles ... 10
4.1 Confidence intervals and p-values ... 10
4.2 Adherence and protocol deviations ... 10
4.3 Analysis populations ... 11
Table 3a. Estimand framework: primary outcome for superiority comparisons, resembling intention to treat approach. ... 12
Table 3b. Estimand framework: primary outcome for non-inferiority comparison, resembling per protocol approach [1]. ... 13
5. Trial population ... 14
5.1 Screening data ... 14
5.2 Eligibility ... 14

DocuSign Envelope ID: 880810C0-2986-4529-AFF3-3AB6B2CC01E3

5.3 Recruitment ... 14
5.4 Withdrawal/follow-up ... 14
5.5 Baseline patient characteristics ... 14
6. Analysis ... 14
6.1 Outcome definitions ... 15
Blood pressure ... 15
Adherence ... 15
Laboratory parameters ... 16
Table 4. Laboratory parameter categorisations as per CTCAE version 5. ... 16
Kidney impairment ... 16
Table 5a. KDIGO eGFR stages. ... 16
Table 5b. KDIGO albumin creatinine ratio (ACR). ... 17
Retinopathy ... 17
Table 6. Hypertensive retinopathy features and grades. ... 17
Changes in surrogate markers for hypertension-mediated organ damage ... 17
Table 7. Surrogate markers for hypertension-mediated organ damage. ... 17
Clinical end organ damage ... 18
Table 8. Major cardiovascular events ... 18
Adverse events ... 19
Other definitions ... 19
6.2 Analysis methods ... 19
6.3 Missing data ... 22
Table 9. Variables to be included in multiple imputation. ... 22
6.4 Additional analyses ... 23
6.5 Harms ... 23
6.6 Statistical software ... 24
7. References ... 24
8. Shell tables and figures ... 25
8.1 Flowchart. ... 26
8.2 Enrolment by stratification factors and randomised group. ... 27
8.3 Baseline characteristics by randomised group. ... 27

2. Introduction

2.1 Background and rationale

Arterial hypertension is a major challenge in sub-Saharan Africa due to low awareness in communities, lack of screening programs, high cost of antihypertensive medication and the general lack of evidence regarding optimal antihypertensive treatment for Africans living in sub-Saharan Africa. The World Health Organization (WHO) recommends starting treatment of arterial hypertension with a thiazide diuretic (TZD) or a calcium channel blocker (CCB) and a dual combination, if the target blood pressure is not achieved. Newer antihypertensive drugs and treatment strategies are available but have not been compared to the WHO approach in Africa. With the proposed trial, we aim to close this evidence gap.

2.2 Objectives

The main objective is to compare the efficacy and safety of two combination antihypertensive treatment strategies using locally available antihypertensive drugs, versus the WHO standard, in HIV-positive and HIV-negative people with uncomplicated arterial hypertension.

In a sub-study, we will compare blood pressure measurement techniques (repeated clinic versus ambulatory blood pressure measurement).

In a second sub-study, we will perform qualitative assessment of patients' perception and concepts of arterial hypertension in patients, on treatment challenges, and reasons for non-adherence.

3. Study methods

3.1 Trial design

This is an open-label, two-centre, three-arm, parallel-group randomised controlled trial, conducted at St. Francis Referral Hospital/Ifakara Health Institute, Ifakara, Morogoro, Tanzania, and Mokhotlong Government Hospital, SolidarMed Lesotho, Mokhotlong, Lesotho. Full details of the trial design are available in the published protocol (2).

Trial population

Eligible persons are HIV-positive and -negative adults aged ≥18 years of African descent and black ethnicity with a documented uncomplicated, untreated arterial hypertension (blood pressure ≥140/90mmHg) diagnosed at one of the two study sites. Exclusion criteria are:

- Current hospitalization for any reason
- Not of African descent
- Refusal of an HIV-test or indeterminate HIV test result
- History of cardiovascular event in the last month (anginal pain, stroke, myocardial infarction or diagnosis by a doctor)
- Symptomatic arterial hypertension (blood pressure ≥180/110mmHg plus acute headache or chest pain) or acute cardiovascular event
- acute disease, e.g.:

DocuSign Envelope ID: 880810C0-2986-4529-AFF3-3AB6B2CC01E3

  - fever >37.5°C or other signs of acute concomitant infection
  - Dyspnea/respiratory distress
  - Acute pain
- Clinical signs of hypertension-mediated organ damage
  - heart failure (bilateral pitting edema, bilateral crackles or pleural effusion, distended jugular veins)
  - ischemic heart disease (anginal pain on exertion)
  - signs of current ischemic/hemorrhagic stroke (hemiparesis, loss of consciousness)
- Pregnancy (test required for females 18-45y of age)
- Non-consenting or inability to come for follow-up visits
- creatinine clearance ≤30ml/min by Chronic Kidney Disease Epidemiology Formula (CK-EPI) estimation and measurement with a point-of care creatinine from capillary blood

Trial procedures

Participants will be consecutively enrolled and randomized in the ratio 2:1:2 to the dual combination, triple combination or control arms, respectively. All participants will provide written informed consent. Enrolled participants will receive a clinical evaluation including an interview and physical examination, and a standardized assessment of blood pressure at every visit. Additionally, surrogate markers of hypertension-induced end organ damage will be assessed at baseline and at the end of the study at 24 weeks.

Trial arms

The three arms of the trial are detailed in Table 1.

Table 1. Trial arms.

	Arm A<br>Dual combination	Arm B<br>Triple combination	Arm C<br>Control (standard of care)
Start	Amlodipine 5mg OD +<br>Losartan 50mg OD	Amlodipine 2.5mg OD +<br>Losartan 12.5mg OD +<br>Hydrochlorothiazide 6.25mg OD	Amlodipine 10mg OD
Week 4$	Amlodipine 10mg OD +<br>Losartan 50mg OD<br>*	Amlodipine 5mg OD +<br>Losartan 25mg OD +<br>Hydrochlorothiazide 12.5mg OD<br>*	Amlodipine 10mg OD +<br>Hydrochlorothiazide 25mg OD<br>*
Week 8$	Amlodipine 10mg OD +<br>Losartan 100mg OD<br>*	Amlodipine 10mg OD +<br>Losartan 50mg OD +<br>Hydrochlorothiazide 25mg OD<br>*	Amlodipine 10mg OD +<br>Hydrochlorothiazide 50mg OD<br>*
Week 12-24$	Amlodipine 10mg OD +<br>Losartan 100mg OD<br>*, **	Amlodipine 10mg OD +<br>Losartan 50mg OD +<br>Hydrochlorothiazide 25mg OD<br>*,**	Amlodipine 10mg OD +<br>Hydrochlorothiazide 50mg OD<br>*, **

$ Increases in dosages only if target BP is not reached (see below)

* In case of orthostatic hypotension or adverse events, medication will be reduced to the prior step – or to half of the initial dosage.

**if regimen shows insufficient effect, individualized adaptation possible according the treating physician

Outcomes

As per the protocol, the primary outcome of the trial is:

- The proportion of patients reaching a target blood pressure (clinic BP) of ≤130/80mmHg among patients <65 years of age and ≤140/90mmHg among patients ≥65 years of age at 12 weeks.

As per the protocol, the secondary outcomes of the trial are:

- The proportion of patients reaching a target blood pressure of ≤130/80mmHg among patients <65 years of age and ≤140/90mmHg among patients ≥65 years of age at 4, 8, and 24 weeks
- The reduction in blood pressure (change from enrolment) at 4, 8, 12 and 24 weeks
- Proportion of patients with treatment adaptations made to the primary treatment (dose increases and/or drug additions) within 12 weeks
- Number of treatment adaptations per patient made to the primary treatment within 12 weeks
- Time until (first*) target blood pressure of ≤130/80mmHg in patients <65 years of age and ≤140/90mmHg in patients ≥65 years of age, with censoring at last visit for those not observed to reach the target (*we ignore subsequent rebounds)
- The proportion of patients with changes in surrogate markers for hypertension-mediated organ damage within 24 weeks (resolving, newly occurring or worsening):
    - Kidney impairment: changes in glomerular filtration rate, measured by CK EPI formula, increase in proteinuria according albumin/creatinine ratio
    - Signs of hypertensive heart disease assessed by electrocardiogram (positive Sokolow-Lyon Index defined as Sokolow-Lyon voltage (SV1 + RV5/V6 ≥ 3.5 mV and/or RaVL ≥ 1.1 mV)
    - Hypertensive cardiopathy assessed by remote echocardiography showing signs of left ventricular hypertrophy
    - Left atrial remodeling assessed by remote echocardiography showing left atrial enlargement
    - retinopathy assessed by retinal picture
- The proportion of patients with major cardiovascular endpoints such as death, stroke, myocardial infarction, heart failure) within 24 weeks
- The proportion of patients lost to follow-up (LTFU) or stopped treatment within 24 weeks
- The proportion of patients with at least one grade 3/4 adverse event within 24 weeks
- The proportion of patients with at least one serious adverse event within 24 weeks
- Proportion of patients who were non-adherent to drugs at 12 weeks (<90% pill count or missing pills ≥2 times in the last month, or drug intake different from prescription)
- Reasons for non-adherence (descriptive analysis)
- Cost-effectiveness of the 3 treatment algorithms.

We will assess effect modification of the primary endpoint (proportion of patients reaching target blood pressure as defined above at 12 weeks) by:

- Site (Tanzania/Lesotho)
- HIV status (positive/negative). While HIV-infection is not expected to change response to anti-hypertensive drugs, comedication with antiretrovirals might affect drug levels.

In a substudy of 100 patients in Ifakara, we plan to assess:

- Proportion of patients with white coat hypertension, as determined by 24h ambulatory blood pressure measurement
- Proportion of patients with blood pressure control determined by 24h ambulatory blood pressure measurement (24h mean blood pressure <130/80mmHg irrespective of age).

DocuSign Envelope ID: 880810C0-2986-4529-AFF3-3AB6B2CC01E3

3.2 Randomisation

Randomisation was stratified by site (Tanzania, Mokhotlong), HIV status (positive, negative) and age (<65, ≥65 years) with randomly varying block sizes (details on block sizes held by trial statistician). The randomisation list was generated by computer by an independent statistician (Jan Hattendorf, Swiss TPH). Randomisation was performed using sealed, opaque envelopes, prepared by person(s) independent to the trial. Monitoring of the randomisation process was performed in real time at the start of the trial by data management at Swiss TPH. Randomisations were checked thereafter through regular querying processes.

3.3 Sample size

As per the protocol: The response rate in the control arm is assumed to be 40%. For the superiority comparison between the triple combination and control arms, we assume an improvement in the triple combination arm of 15% (two-sided alpha of 0.05). For the non-inferiority comparison between the dual combination and control arms, we assume a non-inferiority margin of 10% (one-sided alpha of 0.025). A sample size of 431 patients in each of the control and dual combination arms, and 216 patients in the triple combination arm, yields a power of 85% for the non-inferiority comparison and a power of 95% for the superiority comparison. The overall sample size is therefore 1078 patients, with the randomization ratio of 2:1:2 for the dual combination, triple combination and control arms, respectively. Assuming 15% of participants will become lost-to-follow-up (60) brings the total required sample size to 1268 individuals. Each site will enrol at least 500 participants, after which enrolment will be competitive.

3.4 Framework

The comparison between the triple combination and control arms is superiority. The comparison between dual combination and control arms is non-inferiority. If the dual combination is found to be non-inferior to the control, then we will assess for superiority.

3.5 Statistical interim analyses and stopping guidance

An IDMC was formed to monitor the progress of the trial. Further details are provided in the IDMC charter. Of note, no formal stopping rules are applied, and the criteria for the strength of evidence is left to the judgement of the IDMC. The final statistical report will include a summary of the IDMC meetings held along with the outcomes of those meetings.

3.6 Timing of final analysis

All outcomes will be analysed after the last participant-last visit.

3.7 Timing of outcome assessments

Baseline is defined as the date of randomisation. Time will be measured from randomisation.

Baseline laboratory results are defined as those measured up to 30 days before randomisation and up to the date of randomisation (inclusive). For consistency, the same window will be applied to creatinine measurements, although according to protocol this should have been repeated at enrolment if not

DocuSign Envelope ID: 880810C0-2986-4529-AFF3-3AB6B2CC01E3

measured within the previous two weeks. Of note, the time of a laboratory result is defined as the time when the sample was taken, not the time when the sample was processed or result available.

Table 2 shows the nominal visit weeks and the permitted ranges as defined in the protocol, and as defined for analysis. The analysis windows are chosen to be as inclusive (wide) as possible while retaining clinical relevance, and without overlap between the windows for different visits. If there is more than one measurement within a given interval, then the main result will be that closest to the nominal week, with preference given to earlier values if equidistant. For analyses by scheduled visits, only time points where there are at least 10 participants in each group with a measurement will be included. Sensitivity analyses will be performed using the protocol-defined windows.

Table 2. Nominal visits and permitted windows.

Nominal visit week	Nominal visit day	Protocol defined window, days (inclusive) [1]	Analysis window, days (inclusive)	Analysis window, weeks
4	28	20-41	21-41	3-<6
8	56	50-70	42-69	6-<10
12	84	78-98	70-125	10-<18
24	168	141-196	126-252	18-36

[1] Days edited to count randomisation as day 0 (instead of day 1 as shown in protocol).

4. Statistical principles

4.1 Confidence intervals and p-values

Statistical tests and confidence intervals will be two-sided. Estimates will be presented with 95% confidence intervals. P-values will be presented where appropriate. No adjustments will be made for multiple testing nor interim analyses. Interpretations will be based on the strength of evidence of effect size and consistency of results for related outcomes.

4.2 Adherence and protocol deviations

Immediately following randomisation, participants are expected to initiate on the drug regimens indicated by their randomisation as per Table 1. Any deviations will be reported.

During follow up, dose increases or drug additions are expected if the participant has not yet reached the target blood pressure (see Table 1). Further, dose reductions or other drug adaptations are permitted in case of adverse events or insufficient effects as determined by the treating physician. Treatment adaptations are a secondary outcome, and their analysis is described further in section 6.2.

Adherence is captured in two ways: pill count and self-report. Adherence is a secondary outcome, and is further described in sections 6.1 and 6.2.

Any protocol deviations as reported by the trial team or detected at the time of data cleaning or analysis will be described by group.

DocuSign Envelope ID: 880810C0-2986-4529-AFF3-3AB6B2CC01E3

4.3 Analysis populations

As per the published protocol (2): "Analyses and reporting will follow CONSORT guidelines and intention-to-treat (ITT) principles, that is including participants as randomized… Appropriate methods such as multiple imputation will be considered to account for participants with missing outcome data… Primary analyses for the non-inferiority comparison will be performed on both the ITT and per protocol sets" (3).

Our approaches for the primary outcome are further detailed in the context of the estimands framework in Tables 3a and 3b (4).

For secondary outcomes related to blood pressure (achieving target blood pressure at other time-points, and changes in blood pressure), we will employ similar methods as for the primary outcome (both superiority and non-inferiority comparisons); however the summary measure for the changes in blood pressure will be mean difference.

For the remaining secondary outcomes, we will employ similar methods as for the primary outcome but under the superiority comparison only.

DocuSign Envelope ID: 880810C0-2986-4529-AFF3-3AB6B2CC01E3

Table 3a. Estimand framework: primary outcome for superiority comparisons, resembling intention to treat approach.

Estimand attribute	Definition	Comments
Population	HIV-positive and -negative adults aged ≥18 years of African descent and black ethnicity with a documented uncomplicated, untreated arterial hypertension (blood pressure ≥140/90mmHg), among those who do not become pregnant nor die from a non CV cause within 12 weeks	Exclusion of pregnant women as defined in the protocol. Exclusion of participants who died from non CV causes within 12 weeks added since we are not interested in treatment effects among such participants.
Treatment conditions	Triple combination vs. control, and dual combination vs. control	With dose increases according to the protocol (see Table 1)
Endpoint	Target blood pressure ≤130/80mmHg among patients <65 years of age and ≤140/90mmHg among patients ≥65 years of age at 12 weeks, and did not die from CV cause	Endpoint clarified to include those who died from a CV cause as "failures", i.e. did not achieve target blood pressure
Summary measure	Odds ratio and risk difference	
Handling of intercurrent events (IE) [1]		
Failure to initiate treatment	"Treatment policy" approach	That is, such IE are ignored and considered part of the intention to treat strategy
Treatment interruption due to AE	"Treatment policy" approach	That is, such IE are ignored and considered part of the intention to treat strategy
<80% adherent (not for AE reason) [2]	"Treatment policy" approach	That is, such IE are ignored and considered part of the intention to treat strategy
Death due to CV	"Composite" approach	That is, death due to CV cause has been included as part of the endpoint definition above
Death due to non CV cause	"While on treatment/while alive" approach	That is, the endpoint prior to the occurrence of the IE (death due to non CV cause) is of interest, and therefore in the definition of the population above we have excluded those who died due to non CV cause within 12 weeks

[1] Intercurrent events are post-randomisation events which affect the interpretation or occurrence of outcome data (missing data or LTFU is not in itself an intercurrent event; see instead section 6.3 Missing data). [2] See section 6.1, adherence definition.

Table 3b. Estimand framework: primary outcome for non-inferiority comparison, resembling per protocol approach [1].

Estimand attribute	Definition	Comments
Population	HIV-positive and -negative adults aged ≥18 years of African descent and black ethnicity with a documented uncomplicated, untreated arterial hypertension (blood pressure ≥140/90mmHg), among those who do not become pregnant nor die from a non CV cause within 12 weeks	Exclusion of pregnant women as defined in the protocol. Exclusion of participants who died from non CV causes within 12 weeks added since we are not interested in treatment effects among such participants.
Treatment conditions	Triple combination vs. control, and dual combination vs. control	With dose increases according to the protocol (see Table 1)
Endpoint	Target blood pressure ≤130/80mmHg among patients <65 years of age and ≤140/90mmHg among patients ≥65 years of age at 12 weeks, and did not die from CV cause, and did not interrupt treatment due to AE	Endpoint clarified to include those who died from a CV cause as "failures", i.e. did not achieve target blood pressure
Summary measure	Odds ratio and risk difference	
Handling of intercurrent events (IE) [2]		
Failure to initiate treatment	"Principal stratum" approach	That is, we are interested in the treatment effect of participants who would start the treatment under either treatment arm. Given the similarity of treatment arms and therefore assuming that failure to initiate treatment is independent of the randomisation, we will exclude participants who do not start treatment in each arm
Treatment interruption due to AE	"Composite" approach	That is, treatment interruption due to AE has been included as part of the endpoint definition above
<80% adherent (not for AE reason) [3]	"Principal stratum" approach	That is, we are interested in the treatment effect of participants who would adhere under either treatment arm. Under the assumption that the reasons for non-adherence are not related to the treatment itself, we will exclude non-adherent participants in each arm
Death due to CV	"Composite" approach	That is, death due to CV cause has been included as part of the endpoint definition above
Death due to non CV cause	"While on treatment/while alive" approach	That is, the endpoint prior to the occurrence of the IE (death due to non CV cause) is of interest, and therefore in the definition of the population above we have excluded those who died due to non CV cause within 12 weeks

[1] Text which differs from Table 3a is shown in blue. [2] Intercurrent events are post-randomisation events which affect the interpretation or occurrence of outcome data (missing data or LTFU is not in itself an intercurrent event; see instead section 6.3 Missing data). [3] See section 6.1, adherence definition.

DocuSign Envelope ID: 880810C0-2986-4529-AFF3-3AB6B2CC01E3

5. Trial population

5.1 Screening data

No specific screening data were captured, aside from those used to determine eligibility for the trial (see sections 3.1 and 5.2).

5.2 Eligibility

Screening/eligibility data will be summarised in a CONSORT flowchart, showing the total number of people screened and the reasons for screening failures as per the eligibility criteria in section 3.1. See template in section 8.

5.3 Recruitment

We will present graphically screening and enrolment over time. The CONSORT flowchart will include the numbers of participants randomised by group. Enrolment will be presented by the stratification factors (site, HIV status, and age; see section 8).

5.4 Withdrawal/follow-up

The CONSORT flowchart will summarise follow-up for each of the scheduled visits (weeks 4, 8, 12 and 24), by randomised group. Reasons will be given for participants who did not complete follow-up as expected, including withdrawals.

5.5 Baseline patient characteristics

Baseline characteristics will be summarised by randomised group, using medians and interquartile ranges for continuous variables and numbers and percentages for categorical variables. A shell table showing the variables and categories is included in section 8. Assessment will be made for baseline imbalances between the randomisation groups by visual inspection only, by the trial team before looking at outcome data. In sensitivity analyses, we will further adjust the outcome analyses for any covariates so identified (erring on the side of inclusivity). There will be no formal testing of baseline characteristics across randomised groups (5,6).

6. Analysis

Analyses will follow CONSORT guidelines, including extensions for multi-arm and non-inferiority trials (6–9). Analyses will include all follow up to the date of data freeze.

Analyses will be performed by the trial statistician. There will be no independent programming of the analyses, rather we rely on the training and experience of the team for the accuracy of the data, analyses and interpretations; and the results will be assessed as a whole for consistency.

Percentages will be reported to zero decimal places, unless <0.5% when they will be given to one decimal place.

6.1 Outcome definitions

Here we define outcomes for analysis; section 6.2 further details how those outcomes will be analysed and presented.

Blood pressure

At screening and each follow-up visit, blood pressure is measured in triplicate, with the final value being the average of the second and third measurements. The average SBP and DBP values will be rounded to nearest whole number (of note, this means that an average SBP of 140.4 mmHg is treated for the purposes of eligibility and outcome determination as 140 mmHg and therefore within the target). Target blood pressure is defined by ≤130/80mmHg among patients <65 years of age and ≤140/90mmHg among patients ≥65 years of age, based on current age. Note that, for example, ≤130/80mmHg means BOTH systolic BP ≤130 mmHg AND diastolic BP ≤80 mmHg.

In addition, we will categorise blood pressure (at both baseline and during follow-up) as defined in the protocol (10), with the highest of SBP and DBP defining the category:

- Grade I hypertension: SBP 140-159 mmHg or DBP 90-99 mmHg
- Grade II hypertension: SBP 160-179 mmHg or DBP 100-109 mmHg
- Grade III hypertension: SBP ≥180 mmHg or DBP ≥110mmHg

Adherence

Adherence will be assessed by pill count, by comparing pills dispensed versus returned over the time between two visits, accounting for the form (full, half or quarters) and type (amlodipine, losartan, HCT) of pills dispensed, to yield a percentage of pills taken (0-100%) during each period between follow up visits (0-4, 4-8, 8-12 and 12-24 weeks), and overall. For participants with >1 drug dispensed, we will pool all drugs together to obtain a single estimate.

At each follow-up visit, self-reported adherence is captured through the question: "how often did the patient miss anti-hypertensive medication in the past 4 weeks?" with possible responses 1=Never, 2=Once, 3=Two-five times, 4=More than five times, 5=Daily. If doses were missed, the patient is asked to give a reason: 1=Forgot, 2=Lost drugs, 3=Side effects, 99=Other.

In addition, the patient is asked to report whether s/he took the medication as per instructions, and if not then which medications/doses and reasons for change.

For the adherence outcome, we will define non-adherence at 12 weeks as those meeting any of the following at their 12 week visit: adherence by pill count since the last visit <90%, or self-report of missed pills ≥2 times in the past 4 weeks, or reported drug intake different from prescription since the last visit equating to <90% of planned doses taken.

For the purposes of the per protocol comparisons (see Table 3b), we will use a threshold of 80% adherence (instead of 90% as above for the adherence outcome), in order not to exclude too many participants. Firstly the prescribed and dispensed treatment must be in accordance with the protocol (see Table 1), otherwise a participant will be considered non-adherent with the randomised regimen. A participant will also be considered non-adherent if they took <80% of their prescribed pills since enrolment according to the pill count, or self-report of missed pills >5 times in the past 4 weeks (at either 4, 8 or 12 week visits), or reported drug intake different from prescription since the last visit equating to <80% of planned doses taken.

DocuSign Envelope ID: 880810C0-2986-4529-AFF3-3AB6B2CC01E3

Laboratory parameters

Laboratory testing is performed at baseline and week 24.

eGFR will be calculated using the CKD-EPI equation (11):

$$eGFR = 141 \times \min\left(\frac{\text{crea}}{\kappa}, 1\right)^{\alpha} \times \max\left(\frac{\text{crea}}{\kappa}, 1\right)^{-1.209} \times 0.993^{age} \times 1.018[\text{if female}] \times 1.159[\text{if black}]$$

where: crea = serum creatinine in mg/dL, κ = 0.7 if female, κ = 0.9 if male, α = -0.329 if female, α = -0.411 if male, min(crea_mgdl/κ,1) = the minimum of crea /κ or 1, max(crea_mgdl/κ,1) = the maximum of crea /κ or 1, and all participants are black as defined by the eligibility criteria. We will use current age in the formula. Creatinine is captured in micromol/L, therefore we will multiply by 0.0113 to obtain in mg/dL before applying the above formula.

In addition to the AEs reported, the laboratory parameters shown in Table 4 will be graded according to CTCAE version 5 (12), with values as indicated in the table.

Table 4. Laboratory parameter categorisations as per CTCAE version 5.

Laboratory measurement	Adverse event in CTCAE	Grade 3	Grade 4	ULN
WBC (10^9 /L)	"Leukopenia"	<2.0 - 1.0 x 10e9 /L	<1.0 x 10e9 /L	-
WBC (10^9 /L)	"Leukocytosis" (high WBC)	>100,000/mm3	- [1]	-
Haemoglobin (g/dL)	"Severe anemia"	<8.0 g/dL	-	-
ALT (IU/L)	"Alanine aminotransferase increased"	>5.0 - 20.0 x ULN if baseline was normal; >5.0 - 20.0 x baseline if baseline was abnormal	>20.0 x ULN if baseline was normal; >20.0 x baseline if baseline was abnormal	46.8

ULN=upper limit of normal. Note that 10^3/microL and 10e9/L are equivalent. [1] Clinical manifestations of leucostasis; this would instead be captured as an adverse event.

Kidney impairment

Kidney impairment will be assessed using KDIGO staging based on eGFR, as shown in Table 5a (13); and the albumin creatinine ratio (ACR), as shown in Table 5b (13,14). ACR results will be excluded if the participant has a urinary tract infection (UTI). UTI will be defined as those with abnormal urinalysis and either of WBC or erythrocytes results "+++" (variable ua2 and ua3 as per database specification).

Of note, if both albumin and creatinine were below the detection limits, the machine determining ACR results would output "---"; we will interpret such results as category A1a. (If albumin and creatinine were both ABOVE the detection limits, then the same result would be shown but this was unlikely in practice.) Some results were captured as for example "<5" meaning that at least one of albumin or creatinine were below the detection limits; we will interpret such results as category A1a. Conversely, some results were captured as for example ">7.5" meaning that at least one of albumin or creatinine were above the detection limits; we will interpret such results as category A3.

Table 5a. KDIGO eGFR stages.

GFR category	GFR (ml/min/1.73 m3)	Terms
G1	≥90	Normal or high

G2	60 – 89	Mildly decreased
G3a	45 - 59	Mildly to moderately decreased
G3b	30-44	Moderately to severely decreased
G4	15-29	Severely decreased
G5	<15	Kidney failure

Table 5b. KDIGO albumin creatinine ratio (ACR).

Category	ACR in mg/mmol	Classification
A1	<1 mg/mmol (referred to as A1a hereafter)<br>1-<3 mg/mmol (referred to as A1b hereafter)	Normal to mildly increased
A2	3-30 mg/mmol	Moderately increase = microalbuminuria
A3	>30 mg/mmol	Severely increased = albuminuria

Retinopathy

Hypertensive retinopathy (HTR) is assessed through fundoscopy at baseline and week 24, with interpretations performed remotely by an expert. The quality of pictures will be summarised (good, adequate, poor, too poor for interpretation [the latter excluded from analyses]). The overall outcome of HTR is binary (yes/no). HTR is described further through "features" and "grade" as indicated in Table 6, with higher grades indicating worse HTR.

Table 6. Hypertensive retinopathy features and grades.

HTR grade	HTR features
1	Mild or moderate generalised arteriolar narrowing. (1)<br>Arteriovenous tortuosity. (2)
2	Definite focal narrowing. (3)<br>Arteriovenous nipping. (4)<br>Crossing compression. (5)
3	Copper wiring. (6)<br>Silver wiring. (7)<br>Signs of grade 2 plus retinal hemorrhages. (8)<br>Signs of grade 2 plus exudates. (9)<br>Signs of grade 2 plus cotton wool spots. (10)
4	Severe grade 3 retinopathy plus papilledema. (11)<br>Severe grade 3 retinopathy plus retinal edema. (12)

Changes in surrogate markers for hypertension-mediated organ damage

This secondary outcome will be assessed at 24 weeks according to the definitions shown in Table 7. Of note, "newly occurring" will only be assessed among those considered to NOT already meet the criteria at baseline; and "worsening" and "resolving" will only be assessed among those considered to already meet the criteria at baseline. For completeness, we will also present those never meeting the criteria, and those who met the criteria at baseline but remained the same at follow-up as baseline (i.e. not worsening and not resolving).

Table 7. Surrogate markers for hypertension-mediated organ damage.

Criterion	Newly occurring	Worsening	Resolving
Kidney impairment [1]	1) KDIGO eGFR stage: among those <G3a at baseline, reaching ≥G3a or	1) KDIGO eGFR stage: among those ≥G3a at baseline, reaching a higher stage or	1) KDIGO eGFR stage: among those ≥3a at baseline, reaching a lower stage or

DocuSign Envelope ID: 880810C0-2986-4529-AFF3-3AB6B2CC01E3

	2) KDIGO ACR stage: among those A1 at baseline, reaching ≥A2	2) KDIGO ACR stage: among those A2 at baseline, reaching A3	2) KDIGO ACR stage: among those ≥A2 at baseline, reaching a lower stage
Signs of left ventricular hypertrophy [2]	If echo available [6], left ventricular mass index (defined as left ventricular mass/BSA) >95g/m^2 in women, >115g/m^2 in men [4]. If echo not available [6], MESA ECG LVH criterion: Yes (versus no) [5]	No definition	Those meeting the criteria detailed in the "Newly occurring" column at baseline but not week 24 will be considered "resolving" this outcome
Retinopathy assessed by retinal picture	HTR grade ≥3, among those grade <3 at baseline	Among those grade 3 at baseline, increase to grade 4	Among those grade ≥3 at baseline, decreasing to grade <3 [3]

[1] Strictly we should have two measurements to exclude acute renal impairment; this is a limitation. In cases where the KDIGO and ACR criteria would not result in the same classification (e.g. one worsening but the other resolving), then the worst criterion prevails. [2] The protocol included signs of hypertensive heart disease, signs of left ventricular hypertrophy and left atrial remodelling; these have been condensed into this single criterion for simplification. [3] Note that decreasing from grade 4 to 3 does not meet the criteria of resolving. For those moving from grade 1 to 2, or 2 to 1: we count as never meeting the criteria. [4] Left ventricular mass is defined as 0.8x 1.04x [(IVS + LVID + PWT)^3-LVID^3] + 0.6 grams, where IVS (or IVSD) is interventricular septum diastole (units mm), LVID (or LVEDD) is left ventricular enddiastolic diameter (units mm), and PWT (or PWD) is posterior wall diastole (units mm) (15). [5] MESA ECG LVH criterion is: SV1 + SV2 + RV5 ≥ 4.2 mV (16). [6] At both baseline and 24 weeks.

Clinical end organ damage

A secondary outcome is major cardiovascular (CV) endpoints such as death, stroke, myocardial infarction, heart failure, within 24 weeks. These are defined by ICD-10 codes, as per Table 8.

Table 8. Major cardiovascular events.

Event	ICD-10 code
**Heart failure**	
- Hypertensive heart disease with congestive heart failure	I11.0
- Hypertensive heart disease without congestive heart failure	I11.9
- Hypertensive heart and renal disease with congestive heart failure	I13
- Heart failure	I50*
**Ischemic heart disease**	
- Angina pectoris	I20*
- Actue myocardial infarction	I21*
- Subsequent myocardial infarction	I22*
- Other acute ischaemic heart disease	I24*
- Chronic ischaemic heart disease	I25*
**Stroke**	
- Subarachnoidal hemorrhage	I60*
- Intracerebral hemorrhage	I61*, I62*
- Cerebral infarction	I63*
- Stroke, not specified	I64
**Kidney failure**	
- Chronic kidney disease stage V (GFR <15ml/min, uremia, ESRD)	N18.4, N18.5

DocuSign Envelope ID: 880810C0-2986-4529-AFF3-3AB6B2CC01E3

- Unspecified kidney failure	N19
**Death** (from CV causes)	Will be individually assessed and classified

* indicates all codes starting as indicated.

Adverse events

AEs are captured using ICD-10 coding, WHO 2019 version in Ifarkara and WHO 2010 version in Lesotho. Severity is graded using CTCAE version 4.03 (14 June 2010) in both sites (1=mild, 2=moderate, 3=severe, 4=life-threatening, 5=death). Causality is assessed as definitely, probably, possible, unlikely or not related to the trial intervention.

As per the protocol, a Serious Adverse Event (SAE) is any untoward medical occurrence that:

- Results in death or is life-threatening,
- Requires hospitalization or prolongation of existing hospitalization,
- Results in persistent or significant disability or incapacity,
- Results in persistent or significant disability or incapacity, or
- Causes a congenital anomaly or birth defect.

For SAEs, both the investigator and sponsor-investigator make assessments of causality and severity. Any differences in their assessments will be reported. For the purposes of analysis, the highest grading with respect to causality and severity will be reported (i.e. the sponsor-investigator cannot downgrade causality or severity, but may upgrade it).

Other definitions

BMI will be calculated as weight in kg divided by height in m^2, and categorized according to WHO classifications of underweight (<18.5 kg/m^2), normal (18.5-<25 kg/m^2) and overweight/obese (≥25 kg/m^2) (17).

Body surface area (BSA) will be calculated as the square root of (height in cm * weight in kg / 3600), yielding a value in m^2.

Alcohol use is captured by AUDIT (interview version) at baseline and week 24, yielding an overall score at each timepoint (18). Higher scores indicate higher alcohol use. Scores are classified as zones I (scores 0-7), II (scores 8-15), III (scores 16-19) and IV (scores 20-40) (18).

6.2 Analysis methods

Continuous variables will be inspected using histograms: 1) to assess for outliers which may be queried for accuracy, and 2) to assess whether appropriate transformations are required for analyses. Outcomes will be summarised using means and standard errors for continuous variables and numbers and percentages for categorical variables, by randomised group.

We will summarise treatment over time by randomised group with number and proportions of participants. We will report treatment adaptations made to the primary treatment (i.e. according to the randomisation at baseline) with the number and proportion of participants who made any change and the numbers of changes per patient. We will describe the treatment adaptations (dose

DocuSign Envelope ID: 880810C0-2986-4529-AFF3-3AB6B2CC01E3

changes, drug changes), including whether the adaptations were according to protocol or not, and reasons for such adaptations.

We will summarise (i) adherence by pill count, (ii) self-reported adherence, and (iii) self-reported drug intake different from prescription, by follow up visit and randomised group. We will report the number and proportion of participants who were non-adherent at 12 weeks (see section 6.1) by randomised group.

For each visit, we will summarise by randomised group the mean and 95% CI for absolute SBP and DBP, and similarly for changes versus baseline, and the number and proportion of participants reaching target BP. Results will be illustrated graphically where applicable.

For the primary outcome, we will summarise the numbers and percentages of participants included in the analysis, meeting the composite parts of the endpoint, and experiencing the intercurrent events, as per Tables 3a and 3b. The primary outcome will be assessed using a logistic regression model, with a variable capturing randomisation arm (three categories of control, dual combination or triple combination), adjusted for baseline blood pressure (SBP and DBP as continuous variables) and the randomisation stratification factors (site as binary; HIV status as binary; baseline age as continuous) (19). We will compare each of the intervention arms versus control. Results will be reported as odds ratios with 95% confidence intervals. Further, we will estimate risk differences, with 95% confidence intervals estimated using the delta method (20).

For the non-inferiority comparison between the dual combination and control arms, a CI approach will be used. Under the assumptions of the sample size calculation, the response rate in the control group is expected to be 40% and a non-inferiority margin of 10% corresponds to a response rate of 30% in the dual combination arm; these values yield an odds ratio of (30/70)/(40/60) = 0.64. Therefore, non-inferiority is compatible with the lower bound of the 95% CI for the odds ratio for the primary outcome exceeding 0.64. A figure illustrating the CIs and the non-inferiority margin will be presented. As per section 3.4, if the dual combination is found to be non-inferior to the control, then we will assess for superiority under the superiority comparison approach (Table 3a) by assessing whether the 95% CI for the odds ratio excludes 1.

Effect modification of the primary outcome by site and HIV status will be assessed by incorporating an interaction between arm and site or HIV status, respectively, acknowledging that power will be low.

In sensitivity analyses for the primary outcome, we will (a) fit an unadjusted model, (b) use windows defined in the protocol (see Table 2), (c) adjust for variables determined to be imbalanced between arms, as detailed in section 5.5, (d) adjust for baseline age categorised (<65, ≥65 years), since that is the categorisation used for the randomisation stratification, and (e) adjust for current age similarly categorised, since target BP is determined based on current age (these latter two analyses would not adjust for baseline continuous age).

Binary secondary outcomes (reaching target BP at weeks 4, 8 and 24; any treatment adaptations by 12 weeks; major CV events within 24 weeks; LTFU or stopped treatment within 24 weeks; at least one grade 3/4 AE within 24 weeks; at least one SAE within 24 weeks; non-adherence to drugs at 12 weeks) will be evaluated in a similar way as the primary outcome.

DocuSign Envelope ID: 880810C0-2986-4529-AFF3-3AB6B2CC01E3

Continuous secondary outcomes (change in SBP and DBP versus baseline at 4, 8, 12 and 24 weeks) will be assessed using linear regression models, adjusted for the same variables as in the primary outcome analysis, and reporting adjusted mean differences between arms.

The number of treatment adaptations made to the primary treatment (including those permitted according to the protocol) will be modelled initially as a binary outcome (any vs. no changes) and further as a count outcome using Poisson or negative binomial models as appropriate depending on the distribution of the data.

Time to event outcomes (time to first reaching target BP) will be assessed using appropriate methods, such as Kaplan-Meier estimation and Cox proportional hazards models, adjusted for the same variables as in the primary outcome analysis. A limitation of this analysis is the infrequent measuring of BP. Participants who were not observe to reach the target BP will be censored at the date that the BP was last measured. Any subsequent rebounds after first reaching target BP will be summarised, but ignored for the purposes of the time to event analysis.

The following laboratory parameters will be summarised as median, IQR, mean and SD at baseline, and mean and 95% CI at week 24 (indicating the numbers of participants with each measurement, and reasons for missingness): WBC, haemoglobin, haematocrit, thrombocytes, neutrophils, neutrophils %, lymphocytes, lymphocytes %, eGFR, ALAT, and random blood sugar. Urinanalysis result will be reported as number and proportion abnormal, at baseline and week 24. The laboratory parameters shown in Table 3 will be presented as the number and proportion of participants with grade 3 and 4 levels, by randomised group and visit (baseline or week 24). The components of the surrogate markers for hypertension-medicated organ damage (including kidney impairment as measured by KDIGO and ACR, left ventricular mass index, and HTR; see section 6.1) will be summarised accordingly.

By each follow-up visit, we will summarise vitals (namely weight, respiratory rate, heart rate, temperature, oxygen saturation) using mean and 95% CI. Physical examination findings and symptoms will be summarised as the number and proportion of participants with each respective finding, sign or symptom, along with the functional status categories (working, ambulatory but not working, bedridden). Smoking will be summarised by the categories current, previous, never, refused to answer.

We will summarise AEs (overall, and separately grade 3/4 AEs) and SAEs as the total number of events, the number of participants with at least one event and the median (IQR) number of events per participant (overall and by randomised group). We will summarise events by ICD-10, severity, causality, actions taken, and outcomes (and SAEs by reason; see section 6.1). AEs and SAEs will also be presented as listings.

We will present AUDIT results at week 24, as number of participants with results and the mean and 95% CI, by randomised group. We will present the number and percentage of participants by category (see section 6.1).

We will summarise co-medications, categorised as anti-infectives (antibiotics, antihelminths, antimalarials, antifungals), antiretrovirals, pain killers, cardiac medication, others.

DocuSign Envelope ID: 880810C0-2986-4529-AFF3-3AB6B2CC01E3

6.3 Missing data

Missing baseline and outcome data will be summarised by study arm. Where applicable, percentages will be of non-missing values.

For the primary outcome and the secondary outcomes relating to blood pressure (achieving target blood pressure at each time-point, changes in blood pressure, and time to target blood pressure), we will use multiple imputation to account for blood pressure data missing due to non-attendance or withdrawal of consent, under the assumption of data missing at random. (Of note, those who are known to have died will be handled accordingly as intercurrent events as per Tables 3a and 3b.) We will used chained equations with 50 imputed datasets (21,22). In the imputations, we will use baseline variables plus follow-up data including SBP, DBP and treatment adherence (Table 9). We will impute missing values of SBP and DBP as continuous variables, and then use these values to determine whether target BP was reached at 12 weeks (23). We will perform the imputation separately by randomised arm (24).

Table 9. Variables to be included in multiple imputation.

Variable	Timepoints	Model specification
SBP	Baseline and weeks 4, 8, 12, 24	Continuous
DBP	Baseline and weeks 4, 8, 12, 24	Continuous
Site	Baseline	Ifakara, Lesotho (logit)
HIV status	Baseline	Negative, positive (logit)
Age	Baseline	Continuous
Sex	Baseline	Female, male (logit)
Marital status	Baseline	Single, married/cohabiting, divorced/separated/widowed (mlogit)
Occupation	Baseline	Work for regular income, work for irregular income, farming, other (mlogit)
Education	Baseline	Captured as number of years completed; to be categorised e.g. none, completed primary education, beyond primary education; categories to be informed by frequencies in data (logit or mlogit depending on whether two or more categories)
Smoking	Baseline	Current, vs not (logit)<br>“refuses to answer” will be treated as missing data
Alcohol	Baseline and weeks 4, 8, 12, 24	AUDIT score zones I, II, III, IV (see section 6.1; mlogit)
Family history of heart attack/stroke	Baseline	No, yes (logit)
Previous history of arterial hypertension	Baseline	No, yes (logit)
Diabetes mellitus	Baseline	No, yes (logit)
BMI	Baseline	Continuous
eGFR	Baseline and week 24	≤G3a (no, yes; see Table 5a; logit)

DocuSign Envelope ID: 880810C0-2986-4529-AFF3-3AB6B2CC01E3

Adherence [1]	Baseline and weeks 4, 8, 12, 24	No, yes (logit)
Adverse event	At any time during follow up	Experienced an AE, vs not (logit; excluding major CV events which are captured separately)
Major CV event	At any time during follow up	No, yes (logit)

Variables selected are those thought to be associated with either the outcome (blood pressure) or non-attendance, or both. We will visually assess continuous outcomes for approximate Normality and consider transformations and/or truncations in the modelling if necessary. For categorical outcomes, some categories may be combined if frequencies are low. [1] Defined by adherent yes/no at each visit, with non-adherent being defined as for the secondary outcome based on 90% threshold (see section 6.1).

For the primary outcome, we will perform a second (deterministic) imputation approach where, for the same participants as above (namely those who did not attend or withdrew consent before the week 12 visit), we impute whether they achieved the primary outcome (achieved target BP at 12 weeks) or not according to the following criteria:

- If a participant also did not return to the clinic for the week 8, we assume that the participant did not achieve the primary outcome, since it is unlikely they are obtaining medications elsewhere and therefore will have been off treatment for at least 4 weeks.
- If a participant did attend their 8 week visit, had met the target BP at that time, and received 4 more weeks of medications, we assume that the participant met the primary outcome, since it is likely that they remained on their treatment through to week 12.
- If a participant did attend their 8 week visit, but did not meet the target BP at that time, then we assume that the participant did not meet the primary outcome, under the assumption that they were non-adherent to their treatment at week 8 and therefore remained non-adherent through to week 12.

For comparison purposes, in addition we will perform complete case analyses, including only the participants with blood pressure results at the respective visits. This would be a sensitivity analysis under the assumption of data missing completely at random. For the time to target blood pressure outcome, participants will be censored at the last visit before any missed visit.

For the secondary outcomes of number of treatment adaptations, changes in surrogate markers for hypertension-mediated organ damage, major CV events, adverse events, and adherence, we will perform complete case analyses. In addition, for the outcomes of major CV events, and adverse events, we will perform analyses under the assumption that participants who did not attend or withdrew consent before week 12 did not experience the event of interest.

6.4 Additional analyses

Separate SAPs will be developed for the 24h BP sub-study, the qualitative sub-study, and for the cost-effectiveness analyses.

6.5 Harms

Safety data are included as secondary endpoints (see section 6.2).

DocuSign Envelope ID: 880810C0-2986-4529-AFF3-3AB6B2CC01E3

We will report by randomised group on the number of pregnancies, the timing of pregnancy relative to entry into the trial, treatment adaptations, and safety outcomes.

6.6 Statistical software

Analyses will be conducted in Stata version 16 (25).

7. References

1. Gamble C, Krishan A, Stocken D, Lewis S, Juszczak E, Doré C, et al. Guidelines for the Content of Statistical Analysis Plans in Clinical Trials. JAMA. 2017 19;318(23):2337–43.

2. Mapesi H. The coArtHA Trial - Identifying the Most Effective Treatment Strategies to Control Arterial Hypertension in Sub-Saharan Africa: Study Protocol for a Randomized Controlled Trial. 2020 Dec 16 [cited 2021 Jan 11]; Available from: https://www.researchsquare.com/article/rs-60225/v1

3. Committee for Proprietary Medicinal Products. Points to consider on switching between superiority and non-inferiority. Br J Clin Pharmacol. 2001 Sep;52(3):223–8.

4. European Medicines Agency (EMA). ICH E9 (R1) addendum on estimands and sensitivity analysis in clinical trials to the guideline on statistical principles for clinical trials. 2020.

5. Pocock S, et al. Subgroup analysis, covariate adjustment and baseline comparisons in clinical trial reporting: current practice and problems. Stat Med. 2002;21:2917–30.

6. The CONSORT Group. CONSORT Statement [Internet]. [cited 2012 Mar 22]. Available from: http://www.consort-statement.org/consort-statement/13-19---results/item15_baseline-data/

7. Schulz KF, Altman DG, Moher D, CONSORT Group. CONSORT 2010 statement: updated guidelines for reporting parallel group randomised trials. BMJ. 2010 Mar 23;340:c332.

8. Juszczak E, Altman DG, Hopewell S, Schulz K. Reporting of Multi-Arm Parallel-Group Randomized Trials: Extension of the CONSORT 2010 Statement. JAMA. 2019 Apr 23;321(16):1610–20.

9. Piaggio G, Elbourne DR, Pocock SJ, Evans SJW, Altman DG, CONSORT Group. Reporting of noninferiority and equivalence randomized trials: extension of the CONSORT 2010 statement. JAMA. 2012 Dec 26;308(24):2594–604.

10. Williams B, Mancia G, Spiering W, Agabiti Rosei E, Azizi M, Burnier M, et al. 2018 ESC/ESH Guidelines for the management of arterial hypertension. Eur Heart J. 2018 Sep 1;39(33):3021–104.

11. Levey AS, Stevens LA, Schmid CH, Zhang YL, Castro AF 3rd, Feldman HI, et al. A new equation to estimate glomerular filtration rate. Ann Intern Med. 2009;150(9):604–12.

12. US Department of Health and Human Sciences. Common Terminology Criteria for Adverse Events (CTCAE) version 5.0 [Internet]. 2017 [cited 2022 Jun 28]. Available from: https://ctep.cancer.gov/protocoldevelopment/electronic_applications/docs/CTCAE_v5_Quick_Reference_8.5x11.pdf

DocuSign Envelope ID: 880810C0-2986-4529-AFF3-3AB6B2CC01E3

13. KDIGO 2012 Clinical Practice Guideline for the Evaluation and Management of Chronic Kidney Disease. Kidney Int Suppl. 2013 Jan;3(1):5–14.

14. Hodel NC, Hamad A, Praehauser C, Mwangoka G, Kasella IM, Reither K, et al. The epidemiology of chronic kidney disease and the association with non-communicable and communicable disorders in a population of sub-Saharan Africa. PloS One. 2018;13(10):e0205326.

15. Lang RM, Badano LP, Mor-Avi V, Afilalo J, Armstrong A, Ernande L, et al. Recommendations for cardiac chamber quantification by echocardiography in adults: an update from the American Society of Echocardiography and the European Association of Cardiovascular Imaging. J Am Soc Echocardiogr Off Publ Am Soc Echocardiogr. 2015 Jan;28(1):1-39.e14.

16. Jain A, Tandri H, Dalal D, Chahal H, Soliman EZ, Prineas RJ, et al. Diagnostic and Prognostic Utility of ECG for Left Ventricular Hypertrophy Defined by MRI in Relationship to Ethnicity: The Multi-Ethnic Study of Atherosclerosis (MESA). Am Heart J. 2010 Apr;159(4):652–8.

17. World Health Organization. Body mass index - BMI [Internet]. [cited 2017 Feb 23]. Available from: http://www.euro.who.int/en/health-topics/disease-prevention/nutrition/a-healthy-lifestyle/body-mass-index-bmi

18. Babor T, Higgins-Biddle J, Saunders J, Monteiro M. The alcohol use disorders identification test (AUDIT). Guidelines for use in primary care. Second edition. WHO. Department of mental health and substance dependence; 2001.

19. Kahan BC, Morris TP. Reporting and analysis of trials using stratified randomisation in leading medical journals: review and reanalysis. BMJ. 2012 Sep 14;345:e5840.

20. Norton EC, Miller MM, Kleinman LC. Computing Adjusted Risk Ratios and Risk Differences in Stata. Stata J Promot Commun Stat Stata. 2013 Sep;13(3):492–509.

21. Jakobsen JC, Gluud C, Wetterslev J, Winkel P. When and how should multiple imputation be used for handling missing data in randomised clinical trials – a practical guide with flowcharts. BMC Med Res Methodol. 2017 Dec 6;17(1):162.

22. Sterne JAC, White IR, Carlin JB, Spratt M, Royston P, Kenward MG, et al. Multiple imputation for missing data in epidemiological and clinical research: potential and pitfalls. BMJ. 2009 Jun 29;338:b2393.

23. Floden L, Bell ML. Imputation strategies when a continuous outcome is to be dichotomized for responder analysis: a simulation study. BMC Med Res Methodol. 2019 Jul 23;19(1):161.

24. Sullivan TR, White IR, Salter AB, Ryan P, Lee KJ. Should multiple imputation be the method of choice for handling missing data in randomized trials? Stat Methods Med Res. 2016 Dec 19;0962280216683570.

25. StataCorp. Stata Statistical Software: Release 16. College Station, TX: StataCorp LLC; 2019.

8. Shell tables and figures

Presented below are the shell tables and figures for the participant flow and baseline characteristics, to illustrate the variables included. Further tables will be included in the report to present the analyses described above, including outcomes and safety data.

8.1 Flowchart.

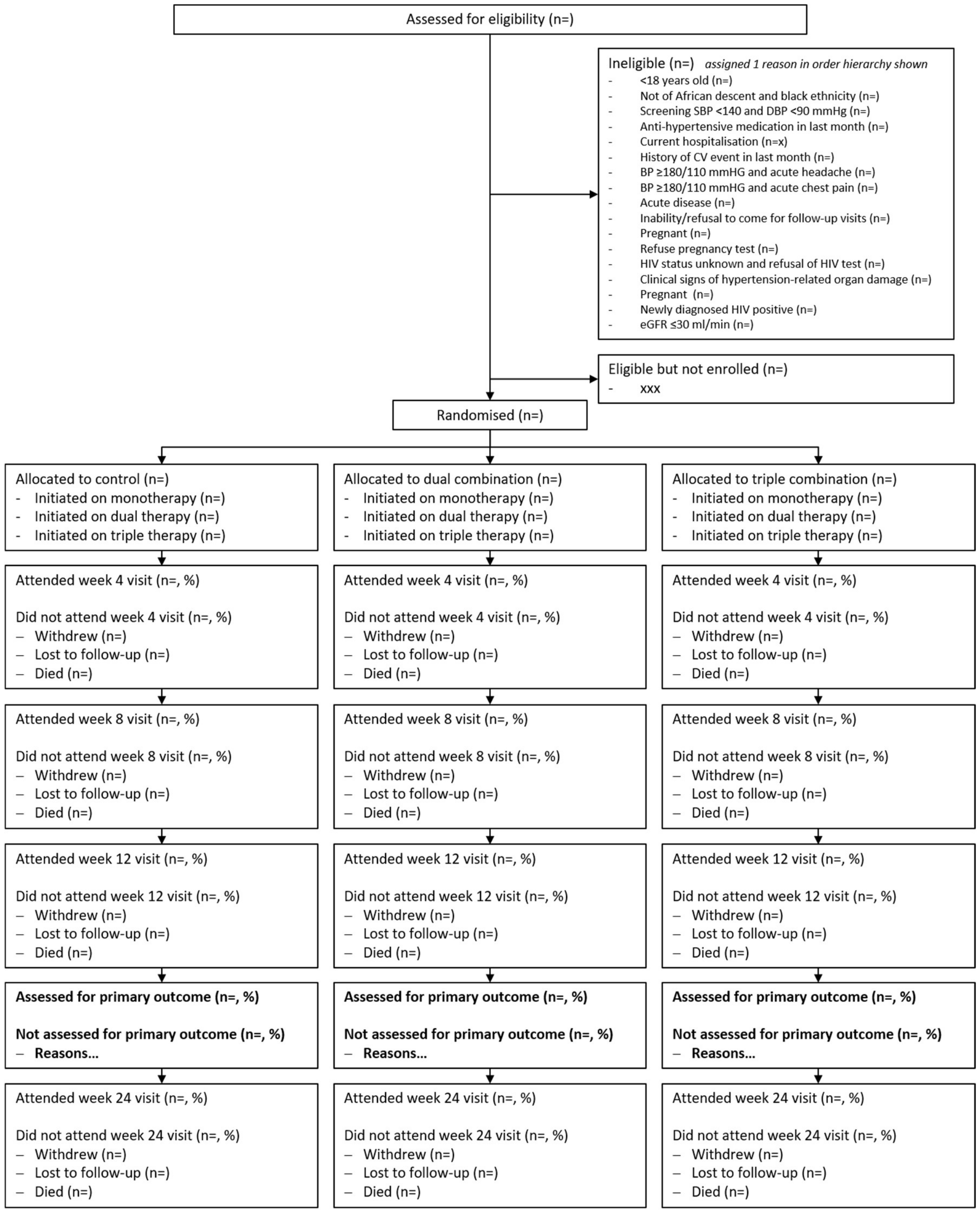

8.2 Enrolment by stratification factors and randomised group.

	Control	Dual therapy	Triple therapy	Total
Number randomised				
Site				
Ifakara				
Mokhotlong				
HIV status				
Positive				
Negative				
Age, years				
<65				
≥65				

Values are number (column %).

8.3 Baseline characteristics by randomised group.

	Control	Dual therapy	Triple therapy	Total
Number randomised				
**Demographics and socio-economics**				
Sex, female				
Age, years				
Marital status				
Occupation				
Education, number of years completed				
Health insurance				
**Toxic habits/CV risk factors**				
AUDIT score				
Cigarette smoking				
Family history of heart attack/stroke [1]				
**Medical history**				
Previous diagnosis of arterial hypertension				
History of tuberculosis				
Among those HIV positive: on ART?				
Diabetes mellitus				
Hypercholesterinemia				
Other chronic diseases				
Any other current medication				
**Current symptoms**				
Any complaints				
**Vitals**				
Weight, kg				
BMI				
Respiratory rate				
Heart rate				

DocuSign Envelope ID: 880810C0-2986-4529-AFF3-3AB6B2CC01E3

Temperature				
Oxygen saturation				
**Functional status**				
Pulmonary signs				
Cardiac signs				
Fluid overload				
Gastrointestinal signs				
Neurological signs				
Lymphadenopathy				
Other signs				
**Testing**				
Remote echocardiography done				
Echo results				
ECG done				
ECG results				
Remote fundoscopy done				
Fundoscopy results				
Chest X ray, clinically indicated				
Chest X ray done				
Chest X ray result				
KDIGO stage				
ACR category				

Results are number (column % of those with non-missing data) for categorical variables and median (IQR) [range] for continuous variables. [1] Parents or siblings with event aged <60 years.
