# Supplementary material for: Treatment Strategies to Control Blood Pressure in People With Hypertension in Tanzania and Lesotho: A Randomized Clinical Trial
Source: JAMA Cardiol. 2025 Jan 29;10(4):321–33. doi: 10.1001/jamacardio.2024.5124 (PMC11780507; doi:10.1001/jamacardio.2024.5124)
Supplement: Supplement 3. — eAppendix. Statistical Analysis eTable 1. Treatment Schedules eTable 2. Estimand Framework: Primary Outcome for Superiority Comparisons, Resembling Intention-to-Treat Approach eTable 3. Estimand Framework: Primary Outcome for Noninferiority Comparison, Resembling Per Protocol Approach eTable 4. Definition of Outcome of Surrogate Markers for Hypertension-Mediated Organ Damage eTable 5. Definition of Major Cardiovascular Events eTable 6. Primary Outcome: Additional Results eTable 7. Primary Outcome: Effect Modification eTable 8. Blood Pressure and Target Attainment Over Time eTable 9. Blood Pressure Changes at 4, 8, 12, and 24 Weeks eTable 10. Target Blood Pressure at 4, 8, and 24 Weeks (ITT Approach) eTable 11. Treatment Escalations Over Time eTable 12. Treatment Prescribed by Visit Week eTable 13. Adherence to Study Medication eTable 14. Adherence to Medication by Pill Count eTable 15. Left Ventricular Hypertrophy eTable 16. Renal Impairment eTable 17. Post Hoc Analysis of Effect Modification by Age eTable 18. Essential Target Blood Pressure Attainment eFigure 1. Time to Achieve Target Blood Pressure eFigure 2. Treatment Prescribed by Visit Week eFigure 3. Blood Pressure Target Attainment [file jamacardiol-e245124-s003.pdf]

Supplementary Online Content

Mapesi H, Rohacek M, Vanobberghen F, et al. Treatment strategies to control blood pressure in people with hypertension in Tanzania and Lesotho: a randomized clinical trial. *JAMA Cardiol*. Published online January 29, 2025. doi:10.1001/jamacardio.2024.5124

eAppendix. Statistical Analysis

eTable 1. Treatment Schedules

eTable 2. Estimand Framework: Primary Outcome for Superiority Comparisons, Resembling Intention-to-Treat Approach

eTable 3. Estimand Framework: Primary Outcome for Noninferiority Comparison, Resembling Per Protocol Approach

eTable 4. Definition of Outcome of Surrogate Markers for Hypertension-Mediated Organ Damage

eTable 5. Definition of Major Cardiovascular Events

eTable 6. Primary Outcome: Additional Results

eTable 7. Primary Outcome: Effect Modification

eTable 8. Blood Pressure and Target Attainment Over Time

eTable 9. Blood Pressure Changes at 4, 8, 12, and 24 Weeks

eTable 10. Target Blood Pressure at 4, 8, and 24 Weeks (ITT Approach)

eTable 11. Treatment Escalations Over Time

eTable 12. Treatment Prescribed by Visit Week

eTable 13. Adherence to Study Medication

eTable 14. Adherence to Medication by Pill Count

eTable 15. Left Ventricular Hypertrophy

eTable 16. Renal Impairment

eTable 17. Post Hoc Analysis of Effect Modification by Age

eTable 18. Essential Target Blood Pressure Attainment

eFigure 1. Time to Achieve Target Blood Pressure

eFigure 2. Treatment Prescribed by Visit Week

eFigure 3. Blood Pressure Target Attainment

This supplementary material has been provided by the authors to give readers additional information about their work.

© 2025 Mapesi H et al. *JAMA Cardiology*.

eAppendix. Statistical analysis

Outcomes were analysed within an estimand framework[1] (eTables 2 and 3) by randomisation allocation:

Briefly, for the superiority comparison for the primary outcome, the estimand was the odds ratio of achieving target blood pressure and not dying from a cardiovascular cause within 12 weeks, between triple combination versus monotherapy, among eligible adults who did not become pregnant or die from a non-cardiovascular cause. For the non-inferiority comparison, we used the same estimand for a per protocol type approach, except that we excluded participants who did not initiate treatment and those who were <80% adherent due to reasons other than AEs, while participants interrupting treatment due to AEs were considered not to have met the primary outcome.

Sensitivity analyses:

In pre-defined sensitivity analyses for the primary outcome, we fit an unadjusted model, used narrower protocol-defined windows for analysis, adjusted for variables determined based on visual inspection to be imbalanced between arms, adjusted for baseline age categorised (<65, ≥65 years), adjusted for current categorized age, performed complete case analyses, and did a deterministic imputation (assuming participants who previously did not return to the clinic or had uncontrolled blood pressure did not meet the primary outcome, and those who had previously controlled blood pressure and received drugs at the last visit did meet the primary outcome).

© 2025 Mapesi H et al. *JAMA Cardiology*.

eTable 1. Treatment schedules

	Steppd CCB monotherapy strategy (CCB/HCTZ)	Two pills strategy (CCB/ARB)	Three pills strategy (CCB/ARB)
Start	Amlodipine 10 mg OD	Amlodipine 5 mg OD + Losartan 50 mg OD	Amlodipine 2.5 mg OD + Losartan 12.5 mg OD + Hydrochlorothiazide 6.25 mg OD
Week 4 [a]	Amlodipine 10 mg OD [b] + Hydrochlorothiazide 25 mg OD [b]	Amlodipine 10 mg [b] OD + Losartan 50mg OD [b]	Amlodipine 5 mg OD [b]+ Losartan 25 mg OD [b]+ Hydrochlorothiazide 12.5 mg OD [b]
Week 8 [a]	Amlodipine 10 mg OD [b] + Hydrochlorothiazide 50 mg OD [b]	Amlodipine 10 mg [b] OD + Losartan 100 mg OD [b]	Amlodipine 10 mg OD [b] + Losartan 50 mg OD [b] + Hydrochlorothiazide 25 mg OD [b]
Week 12-24 [a]	Amlodipine 10 mg [b,c] OD + Hydrochlorothiazide 50 mg OD [b,c]	Amlodipine 10 mg OD [b,c] + Losartan 100 mg OD [b,c]	Amlodipine 10 mg OD [b,c] + Losartan 50 mg OD [b,c] + Hydrochlorothiazide 25 mg OD [b,c]

CCB calcium-channel blocker; ARB angiotensin II receptor blocker; HCTZ hydrochlorothiazide; OD once daily.
[a] Dosages were up-titrated monthly if target blood pressure not reached, namely (≤130/80 mm Hg for participants aged <65 years and ≤140/90 mm Hg for participants aged ≥65 years).
[b] In case of orthostatic hypotension or adverse events, medication was reduced to the prior step – or to half of the initial dosage.
[c] If regimen showed insufficient effect, individualized adaptation was done according the treating physician.

© 2025 Mapesi H et al. *JAMA Cardiology*.

eTable 2. Estimand framework: primary outcome for superiority comparisons, resembling intention to treat approach

Estimand attribute	Definition	Comments
Population	HIV-positive and -negative adults aged ≥18 years of African descent and black ethnicity with a documented uncomplicated, untreated hypertension (blood pressure ≥140/90 mm Hg), who would not become pregnant nor die from a non-CV cause within 12 weeks	Exclusion of pregnant women as defined in the protocol. Exclusion of participants who died from non-CV causes within 12 weeks added since we are not interested in treatment effects among such participants
Treatment conditions	Three pills (CCB/ARB/HCZT) vs. stepped CCB monotherapy (CCB/HCZT) strategies, and two pills (CCB/ARB) vs. stepped CCB monotherapy (CCB/HCZT) strategies	Doses defined in eTable 1
Endpoint	Target blood pressure ≤130/80 mm Hg among patients <65 years of age and ≤140/90 mm Hg among patients ≥65 years of age at 12 weeks, and did not die from CV cause	Endpoint clarified to include those who died from a CV cause as "failures", i.e., did not achieve target blood pressure
Summary measure	Odds ratio and risk difference	
Handling of intercurrent events (IE) [a]		
Failure to initiate treatment	"Treatment policy" approach	That is, such IE are ignored and considered part of the intention to treat strategy
Treatment interruption due to AE	"Treatment policy" approach	That is, such IE are ignored and considered part of the intention to treat strategy
<80% adherent (not for AE reason) b	"Treatment policy" approach	That is, such IE are ignored and considered part of the intention to treat strategy
Death due to CV	"Composite" approach	That is, death due to CV cause has been included as part of the endpoint definition above
Death due to non-CV cause	"Principal stratum" approach	That is, the endpoint prior to the occurrence of the IE (death due to non-CV cause) is of interest, and therefore in the definition of the population above we have excluded those who died due to non-CV cause within 12 weeks
Pregnancy	"Principal stratum" approach	That is, the endpoint prior to the occurrence of the IE (pregnancy) is of interest, and therefore in the definition of the population above we have excluded those who became pregnant within 12 weeks

CV cardiovascular; CCB calcium-channel blocker; ARB angiotensin II receptor blocker; HCTZ hydrochlorothiazide

[a] Intercurrent events are post-randomization events which affect the interpretation or occurrence of outcome data (missing data or loss to follow-up is not in itself an intercurrent event).

© 2025 Mapesi H et al. *JAMA Cardiology*.

eTable 3. Estimand framework: primary outcome for non-inferiority comparison, resembling per protocol approach

Estimand attribute	Definition	Comments
Population	HIV-positive and -negative adults aged ≥18 years of African descent and black ethnicity with a documented uncomplicated, untreated hypertension (blood pressure ≥140/90 mm Hg), who would not become pregnant nor die from a non-CV cause within 12 weeks	Exclusion of pregnant women as defined in the protocol. Exclusion of participants who died from non-CV causes within 12 weeks added since we are not interested in treatment effects among such participants
Treatment conditions	Two pills (CCB/ARB) vs. stepped CCB monotherapy (CCB/HCZT) strategies	Doses defined in eTable 1
Endpoint	Target blood pressure ≤130/80 mm Hg among patients <65 years of age and ≤140/90 mm Hg among patients ≥65 years of age at 12 weeks, and did not die from CV cause and did not interrupt treatment due to AE	Endpoint clarified to include those who died from a CV cause as "failures", i.e., did not achieve target blood pressure
Summary measure	Odds ratio and risk difference	
Handling of intercurrent events (IE) [a]		
Failure to initiate treatment	"Principal stratum" approach	That is, we are interested in the treatment effect of participants who would start the treatment under either treatment arm. Given the similarity of treatment arms and therefore assuming that failure to initiate treatment is independent of the randomization, we will exclude participants who do not start treatment in each arm (n=0, 0, 3 participants in the stepped CCB monotherapy (CCB/HCTZ), two pills (CCB/ARB), three pills (CCB/ARB/HCTZ) strategies, respectively)
Treatment interruption due to AE	"Composite" approach	That is, treatment interruption due to AE has been included as part of endpoint definition above (n=37, 30, 10 participants in the stepped CCB monotherapy (CCB/HCTZ), two pills (CCB/ARB), three pills (CCB/ARB/HCTZ) strategies, respectively)
<80% adherent (not for AE reason)	"Principal stratum" approach	That is, we are interested in the treatment effect of participants who would adhere under either treatment arm. Under the assumption that the reasons for non-adherence are not related to the treatment itself, we will exclude non-adherent participants in each arm (n=115, 89, 56 participants in the stepped CCB monotherapy (CCB/HCTZ), two pills (CCB/ARB), three pills (CCB/ARB/HCTZ) strategies, respectively)
Death due to CV	"Composite" approach	That is, death due to CV cause has been included as part of the endpoint definition above (n=1, 3, 0 participants in the stepped CCB monotherapy (CCB/HCTZ), two pills (CCB/ARB), three pills (CCB/ARB/HCTZ) strategies, respectively)

© 2025 Mapesi H et al. *JAMA Cardiology*.

Death due to non-CV cause	"Principal stratum" approach	That is, the endpoint prior to the occurrence of the IE (death due to non-CV cause) is of interest, and therefore in the definition of the population above we have excluded those who died due to non-CV cause within 12 weeks (n=2, 1, 0 participants in the stepped CCB monotherapy (CCB/HCTZ), two pills (CCB/ARB), three pills (CCB/ARB/HCTZ) strategies, respectively)
Pregnancy	"Principal stratum" approach	That is, the endpoint prior to the occurrence of the IE (pregnancy) is of interest, and therefore in the definition of the population above we have excluded those who became pregnant within 12 weeks (n=1, 4, 1 participants in the stepped CCB monotherapy (CCB/HCTZ), two pills (CCB/ARB), three pills (CCB/ARB/HCTZ) strategies, respectively)

CCB calcium-channel blocker; ARB angiotensin II receptor blocker; HCTZ hydrochlorothiazide;
Text which is the same as eTable 2 is shown in grey, to highlight where there are differences.
[a] Intercurrent events are post-randomization events which affect the interpretation or occurrence of outcome data (missing data or loss to follow-up is not in itself an intercurrent event).

© 2025 Mapesi H et al. *JAMA Cardiology*.

eTable 4. Definition of outcome of surrogate markers for hypertension-mediated organ damage

Criterion	Newly occurring	Worsening	Resolving
Renal impairment [a]	1) KDIGO stage [b]: among those <3a at baseline, reaching ≥3a or	1) KDIGO stage: among those ≥3a at baseline, reaching a higher stage or	1) KDIGO stage: among those ≥3a at baseline, reaching a lower stage or
	2) ACR stage [c]: among those 0 at baseline, reaching ≥1	2) ACR stage: among those 1 at baseline, reaching 2	2) ACR stage: among those ≥1 at baseline, reaching a lower stage
Left ventricular hypertrophy	If echo available, left ventricular mass index (defined as left ventricular mass/BSA) >95g/m^2 in women, >115g/m^2 in men [d].	No definition	Those meeting the criteria detailed in the “Newly occurring” column at baseline but not week 24 will be considered “resolving” this outcome
	If no echo available, MESA ECG LVH criterion: Yes (versus no) [e]		

KDIGO Kidney Disease Improving Global Outcomes criteria;[2] ACR albumin creatinine ratio; BSA body surface area; MESA Multi-Ethnic Study of Atherosclerosis.
[a] In cases where the KDIGO and ACR criteria would not result in the same classification, then the worst criterion prevails.
[b] KDIGO stages included G1 eGFR ≥90ml/min, G2 60-89ml/min, G3a 45-59ml/min, G3b30-44ml/min, G4 15-29ml/min, G5 <15ml/min, calculated using the Chronic Kidney Disease Epidemiology Collaboration (CKD-Epi) equation.[3]
[c] ACR stages included stage 0 (normal) = 30mg/g, stage 1 (microalbuminuria) = 30-300mg/g) and stage 2 (albuminuria) = >300mg/g.
[d] Left ventricular mass was defined as 0.8x 1.04x [(IVS + LVID + PWT)^3-LVID^3] + 0.6 grams, where IVS (or IVSD) is interventricular septum diastole (units mm), LVID (or LVEDD) is left ventricular end-diastolic diameter (units mm), and PWT (or PWD) is posterior wall diastole (units mm).[4]
[e] MESA ECG LVH criterion was: SV1 + SV2 + RV5 ≥ 4.2 mV.[5]

© 2025 Mapesi H et al. *JAMA Cardiology*.

eTable 5. Definition of major cardiovascular events

Event	ICD-10 code
**Heart failure**	
- Hypertensive heart disease with congestive heart failure	I11.0
- Hypertensive heart disease without congestive heart failure	I11.9
- Hypertensive heart and renal disease with congestive heart failure	I13
- Heart failure	I50 [a]
**Ischemic heart disease**	
- Angina pectoris	I20 [a]
- Actue myocardial infarction	I21 [a]
- Subsequent myocardial infarction	I22 [a]
- Other acute ischaemic heart disease	I24 [a]
- Chronic ischaemic heart disease	I25 [a]
**Stroke**	
- Subarachnoidal hemorrhage	I60 [a]
- Intracerebral hemorrhage	I61 [a], I62 [a]
- Cerebral infarction	I63 [a]
- Stroke, not specified	I64
**Kidney failure**	
- Chronic kidney disease stage V (GFR <15ml/min, uremia, ESRD)	N18.4, N18.5
- Unspecified kidney failure	N19
**Death** (from cardiovascular causes)	Were individually assessed and classified

* indicates all codes starting as indicated.

© 2025 Mapesi H et al. *JAMA Cardiology*.

eTable 6. Primary outcome: additional results

	Stepped CCB monotherapy strategy (CCB/HCZT) N=505	Two pills strategy (CCB/ARB) N=510	Three pills strategy (CCB/ARB/HCTZ) N=253
**Per protocol type approach for non-inferiority comparison of two pills versus steeped monotheapy strategy arms (complete case analysis)**			
Number included	338	370	NA
Number reached primary outcome (%) [a]	173 (51%)	207 (56%)	NA
Unadjusted odds ratio (95% CI)	Reference	1.21 (0.90,1.63)	NA
Adjusted odds ratio (95% CI) [b]	Reference	1.18 (0.87,1.61)	NA
**Intention to treat type approach for superiority comparisons**			
**Complete case analysis**			
Number included	447	453	219
BP measured	446	450	219
CV related death	1	3	0
Number reached primary outcome (%) [a]	229 (51%)	257 (57%)	131 (60%)
Unadjusted odds ratio (95% CI)	Reference	1.25 (0.96,1.62); p=0.10	1.42 (1.02,1.97); p=0.04
Adjusted odds ratio (95% CI) [b]	Reference	1.21 (0.92,1.60); P=0.17	1.32 (0.94,1.86); p=0.11
**Using protocol defined windows; complete case analysis**			
Number included	413	425	207
Number reached primary outcome (%) [a]	223 (54%)	247 (58%)	129 (62%)
Adjusted odds ratio (95% CI) [b]	Reference	1.14 (0.86,1.52); p=0.37	1.34 (0.94,1.91); p=0.11
**Multiple imputation analysis**			
Number included	501	505	252
% reached primary outcome [a]	49%	55%	57%
Unadjusted odds ratio (95% CI)	Reference	1.28 (0.99,1.66); p=0.06	1.39 (1.01,1.91); p=0.05
Adjusted odds ratio (95% CI) [b]	Reference	1.24 (0.94,1.63); p=0.12	1.28 (0.91,1.79); p=0.16
As above [b], but baseline age categorised	Reference	1.28 (0.97,1.70); p=0.08	1.39 (0.99,1.96); p=0.06
As above [b], but adjust for current age (categorised) [c]	Reference	1.27 (0.96,1.68); p=0.09	1.39 (0.99,1.96); p=0.06
As above [b], and adjusted in addition for sex and previous diagnosis of hypertension [d]	Reference	1.25 (0.95,1.64); p=0.12	1.28 (0.91,1.79); p=0.15
**Deterministic imputation analysis**			
Number included	501	505	252
% reached primary outcome [a]	232 (46%)	264 (52%)	134 (53%)
Unadjusted odds ratio (95% CI)	Reference	1.27 (0.99,1.63); p=0.06	1.32 (0.97,1.78); p=0.08
Adjusted odds ratio (95% CI) [b]	Reference	1.21 (0.92,1.60); p=0.17	1.32 (0.94,1.86); p=0.11

CCB calcium-channel blocker; ARB angiotensin II receptor blocker; HCTZ hydrochlorothiazide; CI confidence interval; CV cardiovascular.
[a] Percentages of those included in the respective analysis.
[b] From logistic regression model with arm, adjusted for site, HIV status, age (linear), baseline SBP (linear), and baseline DBP (linear).
[c] For those who died from CV death, their baseline age category was used, which had not changed by their time of death, and also would not have changed if they have been alive throughout the trial.

© 2025 Mapesi H et al. *JAMA Cardiology*.

[d] Variables that were determined based on visual inspection to be imbalanced between the arms.

© 2025 Mapesi H et al. *JAMA Cardiology*.

eTable 7. Primary outcome: effect modification

	Stepped CCB monotherapy strategy (CCB/HCZT) N=505	Two pills strategy (CCB/ARB) N=510	Three pills strategy (CCB/ARB/HCTZ) N=253
**Site**			
**Complete case analysis**			
Number included			
Ifakara	235	233	112
Lesotho	212	220	107
Number reached primary outcome (%) [a]			
Ifakara	117 (50%)	118 (51%)	62 (55%)
Lesotho	112 (53%)	139 (63%)	69 (64%)
Adjusted odds ratio (95% CI) [b]			
Ifakara	Reference	1.03 (0.70,1.50)	1.15 (0.72,1.83)
Lesotho	Reference	1.46 (0.98,2.18)	1.55 (0.94,2.54)
P value for effect modification	-	0.21	0.39
**Multiple imputation analysis**			
Number included			
Ifakara	265	264	134
Lesotho	236	241	118
% reached primary outcome [a]			
Ifakara	47%	49%	52%
Lesotho	51%	61%	62%
Adjusted odds ratio (95% CI) [b]			
Ifakara	Reference	1.07 (0.73,1.56)	1.12 (0.70,1.78)
Lesotho	Reference	1.47 (0.98,2.19)	1.48 (0.91,2.41)
P value for effect modification	-	0.26	0.42
**HIV status**			
**Complete case analysis**			
Number included			
HIV negative	288	286	135
HIV positive	159	167	84
Number reached primary outcome (%) [a]			
HIV negative	141 (49%)	161 (56%)	79 (59%)
HIV positive	88 (55%)	96 (57%)	52 (62%)
Adjusted odds ratio (95% CI) [b]			
HIV negative	Reference	1.30 (0.92,1.85)	1.37 (0.89,2.11)
HIV positive	Reference	1.08 (0.68,1.69)	1.24 (0.71,2.16)
P value for effect modification	-	0.51	0.78
**Multiple imputation analysis**			
Number included			
HIV negative	320	322	159
HIV positive	181	183	93
% reached primary outcome [a]			
HIV negative	47%	54%	55%
HIV positive	52%	57%	60%
Adjusted odds ratio (95% CI) [b]			
HIV negative	Reference	1.29 (0.91,1.81)	1.28 (0.84,1.95)
HIV positive	Reference	1.17 (0.75,1.82)	1.27 (0.73,2.20)
P value for effect modification	-	0.73	0.98

CCB calcium-channel blocker; ARB angiotensin II receptor blocker; HCTZ hydrochlorothiazide;
[a] Percentages of those included in the respective analysis.
[b] From logistic regression model with arm, adjusted for site, HIV status, age (linear), baseline SBP (linear), and baseline DBP (linear).

© 2025 Mapesi H et al. *JAMA Cardiology*.

eTable 8. Blood pressure and target attainment over time

	Complete case analysis			Multiple imputation analysis		
	**Stepped CCB monotherapy strategy (CCB/HCZT) N=505**	**Two pills strategy (CCB/ARB) N=510**	**Three pills strategy (CCB/ARB/HCTZ) N=253**	**Stepped CCB monotherapy strategy (CCB/HCZT) N=505**	**Two pills strategy (CCB/ARB) N=510**	**Three pills strategy (CCB/ARB/HCTZ) N=253**
**Baseline**						
SBP, mm Hg (standard deviation)	154 (18)	152 (18)	153 (19)	NA	NA	NA
DBP, mm Hg (standard deviation)	101 (11)	100 (11)	99 (11)	NA	NA	NA
**Week 4**						
Have result [a]	444 (88%)	467 (92%)	231 (91%)	503 [e]	508 [e]	253 [e]
SBP, mm Hg	128 (0.7)	129 (0.8)	133 (1.3)	128 (0.7)	130 (0.8)	134 (1.4)
SBP change, mm Hg	-26 (0.8)	-23 (0.8)	-19 (1.2)	-26 (0.8)	-23 (0.8)	-19 (1.2)
DBP, mm Hg	84 (0.4)	86 (0.5)	88 (0.8)	85 (0.4)	86 (0.5)	88 (0.8)
DBP change, mm Hg	-16 (0.5)	-14 (0.5)	-12 (0.7)	-16 (0.5)	-14 (0.5)	-12 (0.7)
**Target BP [b,c]**	**174 (39%)**	**181 (39%)**	**61 (26%)**	**37%**	**37%**	**26%**
**Essential target BP [b,d]**	**317 (71%)**	**311 (67%)**	**127 (55%)**	**70%**	**65%**	**53%**
Target SBP [b,c]	302 (68%)	309 (66%)	122 (53%)	66%	64%	51%
Essential target SBP [b,d]	371 (84%)	371 (79%)	160 (69%)	83%	77%	67%
Target DBP [b,c]	205 (46%)	197 (42%)	78 (34%)	43%	41%	34%
Essential target DBP [b,d]	355 (80%)	336 (72%)	149 (65%)	78%	70%	64%
Target BP reduction	260 (59%)	249 (53%)	101 (44%)			
**Week 8**						
Have result [a]	427 (85%)	445 (87%)	222 (88%)	501 [e]	505 [e]	252 [e]
SBP, mm Hg	125 (0.7)	124 (0.8)	126 (1.2)	125 (0.7)	125 (0.8)	127 (1.2)
SBP change, mm Hg	-29 (0.9)	-28 (0.9)	-26 (1.3)	-28 (0.9)	-28 (0.8)	-26 (1.3)
DBP, mm Hg	83 (0.4)	82 (0.5)	83 (0.7)	83 (0.4)	83 (0.5)	84 (0.7)
DBP change, mm Hg	-18 (0.5)	-17 (0.5)	-16 (0.7)	-17 (0.5)	-17 (0.5)	-16 (0.7)
**Target BP [b,c]**	**201 (47%)**	**236 (53%)**	**95 (43%)**	**44%**	**51%**	**41%**
**Essential target BP [b,d]**	**324 (76%)**	**337 (76%)**	**156 (70%)**	**74%**	**74%**	**68%**
Target SBP [b,c]	319 (75%)	340 (76%)	156 (70%)	73%	75%	68%
Essential target SBP [b,d]	366 (86%)	379 (85%)	183 (82%)	84%	84%	80%
Target DBP [b,c]	225 (53%)	258 (58%)	110 (50%)	50%	56%	48%
Essential target DBP [b,d]	352 (82%)	360 (81%)	172 (77%)	80%	80%	75%
Target BP reduction	270 (63%)	285 (64%)	124 (56%)			
**Week 12**						
Have result [a]	446 (88%)	450 (88%)	219 (87%)	5003 [e]	5023 [e]	2523 [e]
SBP, mm Hg	125 (0.7)	125 (0.9)	123 (1.2)	125 (0.7)	126 (0.9)	124 (1.2)
SBP change, mm Hg	-29 (0.9)	-27 (0.9)	-29 (1.4)	-28 (0.9)	-27 (0.9)	-29 (1.3)
DBP, mm Hg	83 (0.5)	82 (0.5)	80 (0.6)	83 (0.4)	83 (0.5)	81 (0.6)
DBP change, mm Hg	-18 (0.5)	-17 (0.6)	-19 (0.8)	-17 (0.5)	-17 (0.5)	-19 (0.8)
**Target BP [b,c]**	**229 (51%)**	**257 (57%)**	**131 (60%)**	**49%**	**55%**	**57%**
**Essential target BP [b,d]**	**342 (77%)**	**349 (78%)**	**179 (82%)**	**75%**	**76%**	**79%**
Target SBP [b,c]	335 (75%)	349 (78%)	168 (77%)	74%	76%	74%
Essential target SBP [b,d]	381 (85%)	382 (85%)	190 (87%)	85%	84%	84%
Target DBP [b,c]	252 (57%)	281 (62%)	143 (65%)	54%	61%	63%
Essential target DBP [b,d]	366 (82%)	372 (83%)	195 (89%)	81%	81%	87%
Target BP reduction	278 (62%)	282 (63%)	137 (63%)			
**Week 24**						
Have result [a]	420 (83%)	436 (85%)	215 (85%)	4943 [e]	4983 [e]	2513 [e]

© 2025 Mapesi H et al. *JAMA Cardiology*.

SBP, mm Hg	126 (0.9)	126 (0.9)	125 (1.2)	126 (0.9)	126 (0.8)	125 (1.3)
SBP change, mm Hg	-27 (1.0)	-26 (0.9)	-28 (1.4)	-27 (0.9)	-26 (0.9)	-28 (1.4)
DBP, mm Hg	83 (0.5)	83 (0.5)	81 (0.7)	83 (0.5)	83 (0.5)	81 (0.7)
DBP change, mm Hg	-18 (0.6)	-17 (0.6)	-18 (0.8)	-18 (0.6)	-17 (0.6)	-18 (0.8)
**Target BP [b,c]**	223 (53%)	230 (53%)	121 (56%)	**50%**	**51%**	**54%**
**Essential target BP [b,d]**	**314 (75%)**	**331 (76%)**	**163 (76%)**	**73%**	**74%**	**74%**
Target SBP [b,c]	319 (76%)	319 (73%)	156 (73%)	74%	72%	71%
Essential target SBP [b,d]	344 (82%)	370 (85%)	177 (82%)	81%	83%	81%
Target DBP [b,c]	239 (57%)	250 (57%)	134 (62%)	54%	56%	60%
Essential target DBP [b,d]	337 (80%)	350 (80%)	176 (82%)	79%	79%	81%
Target BP reduction	263 (63%)	258 (59%)	138 (64%)			

CCB calcium-channel blocker; ARB angiotensin II receptor blocker; HCTZ hydrochlorothiazide; BP blood pressure; SBP systolic blood pressure; DBP diastolic blood pressure; SD standard deviation. Results are number (column%) or mean (standard error) except where otherwise indicated. All changes are relative to baseline.

[a] Percentage of those randomized.

[b] Percentage of those with a result.

[c] Target BP reached based on primary outcome definition, namely ≤130/80 mm Hg in those aged <65 years and ≤140/90 mm Hg in those aged ≥65 years.

[d] Target BP reached based on guidelines of the International Hypertension Society, namely ≤140/90 mm Hg regardless of age.[6]

[e] Values were multiply imputed for those who did not have BP measured, except for those pregnant or who died, hence values are not total number randomized.

© 2025 Mapesi H et al. *JAMA Cardiology*.

eTable 9. Blood pressure changes at 4, 8, 12, and 24 weeks

	Stepped CCB monotherapy strategy (CCB/HCZT) N=505	Two pills strategy (CCB/ARB) N=510	Three pills strategy (CCB/ARB/HCTZ) N=253
**WEEK 4**			
**Complete case analysis**			
Number included	444	467	231
Adjusted mean SBP difference (95% CI) [a]	Reference	2.10 (0.23,3.97); p=0.03	6.08 (3.78,8.37); p<0.001
Adjusted mean DBP difference (95% CI) [a]	Reference	1.87 (0.77,2.97); p=0.001	4.07 (2.72,5.42); p<0.001
**Multiple imputation analysis**			
Number included	503	508	253
Adjusted mean SBP difference (95% CI) [a]	Reference	2.28 (0.37,4.19); p=0.02	6.47 (4.08,8.87); p <0.001
Adjusted mean DBP difference (95% CI) [a]	Reference	1.80 (0.68,2.93); p=0.002	3.96 (2.56,5.36); p<0.001
**WEEK 8**			
**Complete case analysis**			
Number included	427	445	222
Adjusted mean SBP difference (95% CI) [a]	Reference	-0.01 (-1.94,1.92); p=0.99	1.52 (-0.84,3.88); p=0.21
Adjusted mean DBP difference (95% CI) [a]	Reference	-0.23 (-1.36,0.89); p=0.68	0.84 (-0.53,2.21); p=0.23
**Multiple imputation analysis**			
Number included	501	505	252
Adjusted mean SBP difference (95% CI) [a]	Reference	-0.19 (-2.10,1.72); p=0.84	1.89 (-0.52,4.31); p=0.12
Adjusted mean DBP difference (95% CI) [a]	Reference	-0.48 (-1.62,0.65); p=0.41	0.90 (-0.49,2.30); p=0.21
**WEEK 12**			
**Complete case analysis**			
Number included	446	450	219
Adjusted mean SBP difference (95% CI) [a]	Reference	0.66 (-1.35,2.68); p=0.52	-1.70 (-4.19,0.78); p=0.18
Adjusted mean DBP difference (95% CI) [a]	Reference	-0.47 (-1.68,0.74); p=0.44	-2.21 (-3.70,-0.71); p=0.004
**Multiple imputation analysis**			
Number included	500	502	252
Adjusted mean SBP difference (95% CI) [a]	Reference	0.76 (-1.27,2.79); p=0.46	-1.18 (-3.66,1.30); p=0.35
Adjusted mean DBP difference (95% CI) [a]	Reference	-0.52 (-1.72,0.69); p=0.40	-2.17 (-3.64,-0.69); p=0.004
**WEEK 24**			
**Complete case analysis**			
Number included	420	436	215
Adjusted mean SBP difference (95% CI) [a]	Reference	0.33 (-1.76,2.41); p=0.76	-1.56 (-4.12,0.99); p=0.23
Adjusted mean DBP difference (95% CI) [a]	Reference	0.04 (-1.23,1.31); p=0.95	-1.11 (-2.67,0.44); p=0.16
**Multiple imputation analysis**			
Number included	494	498	251
Adjusted mean SBP difference (95% CI) [a]	Reference	0.38 (-1.72,2.48); p=0.73	-1.26 (-3.87,1.36); p=0.35
Adjusted mean DBP difference (95% CI) [a]	Reference	-0.04 (-1.33,1.25); p=0.95	-1.07 (-2.69,0.54); p=0.19

CCB calcium-channel blocker; ARB angiotensin II receptor blocker; HCTZ hydrochlorothiazide; BP Blood pressure; SBP systolic BP; DBP diastolic BP; CI Confidence interval.

[a] From linear regression model with arm (three categories), adjusted for site (Ifakara, Mokhotlong), HIV status (positive, negative), age (linear), baseline SBP (linear), and baseline DBP (linear).

© 2025 Mapesi H et al. *JAMA Cardiology*.

eTable 10. Target blood pressure at 4, 8, and 24 weeks (ITT approach)

	Stepped CCB monotherapy strategy (CCB/HCZT) N=505	Two pills strategy (CCB/ARB) N=510	Three pills strategy (CCB/ARB/HCTZ) N=253
**WEEK 4**			
Number in whom outcome assessed [a]	445 (88%)	468 (92%)	231 (91%)
BP measured	444	467	231
CV death	1	1	0
**Complete case analysis**			
Number included	445	468	231
Number reached outcome (%) [b]	174 (39%)	181 (39%)	61 (26%)
Adjusted odds ratio (95% CI) [c]	Reference	0.92 (0.69,1.24); p=0.60	0.47 (0.32,0.69); p<0.001
**Multiple imputation analysis**			
Number included	503	509	253
% reached outcome [b]	37%	37%	26%
Adjusted odds ratio (95% CI) [c]	Reference	0.93 (0.70,1.25); p=0.64	0.49 (0.33,0.72); p<0.001
**WEEK 8**			
Number in whom outcome assessed [a]	428 (85%)	447 (88%)	222 (88%)
BP measured	427	445	222
CV death	1	2	0
**Complete case analysis**			
Number included	428	447	222
Number reached outcome (%) [b]	201 (47%)	236 (53%)	95 (43%)
Adjusted odds ratio (95% CI) [c]	Reference	1.20 (0.90,1.59); p=0.22	0.73 (0.52,1.04); p=0.08
**Multiple imputation analysis**			
Number included	502	507	252
% reached outcome [b]	44%	51%	41%
Adjusted odds ratio (95% CI) [c]	Reference	1.26 (0.95,1.65); p =0.11	0.75 (0.53,1.07); p=0.11
**WEEK 24**			
Number in whom outcome assessed [a]	421 (83%)	439 (86%)	215 (85%)
BP measured	420	436	215
CV death	1	3	0
**Complete case analysis**			
Number included	420	439	215
Number reached outcome (%) [b]	223 (53%)	230 (52%)	121 (56%)
Adjusted odds ratio (95% CI) [c]	Reference	0.95 (0.72,1.26); p=0.73	1.09 (0.77,1.53); p=0.64
**Multiple imputation analysis**			
Number included	495	501	251
% reached outcome [b]	50%	51%	54%
Adjusted odds ratio (95% CI) [c]	Reference	0.98 (0.75,1.30); p =0.91	1.06 (0.75,1.48); p=0.75

CCB calcium-channel blocker; ARB angiotensin II receptor blocker; HCTZ hydrochlorothiazide;
[a] Percentage of those randomized.
[b] Percentages of those included in the respective analysis.
[c] From logistic regression model with arm (three categories), adjusted for site (Ifakara, Mokhotlong), HIV status (positive, negative), age (linear), baseline SBP (linear), and baseline DBP (linear).

© 2025 Mapesi H et al. *JAMA Cardiology*.

eTable 11. Treatment escalations over time

Treatment		Stepped CCB monotherapy strategy (CCB/HCZT) N=505	Two pills strategy (CCB/ARB) N=510	Three pills strategy (CCB/ARB/HCTZ) N=253
**Enrolment**				
First regimen as per randomization		505	510	250
Error in first regimen		0	0	3
**Week 4 (+ unscheduled visits before)**				
No changes (target BP met)	Continue baseline regimen	161	175	59
Dose escalation (target BP not met)	1st escalation	264	279	164
Other	Continue regimen for non-adherence	0	5	2
	Stop treatment due to AE	18	8	5
	Stop treatment due to pregnancy	1	1	0
	Not according protocol/Other [a]	3	0	1
**Week 8 (+ unscheduled visits before)**				
No changes (target BP met)	Continue baseline regimen	117	120	46
	Continue 1st escalation	62	95	42
Dose escalation (target BP not met)	1st escalation	33	45	14
	2nd escalation	157	148	101
Other	Continue due to non-adherence	10	6	4
	Stop treatment due to AE	35	25	7
	Stop treatment due to pregnancy	1	2	1
	Not according protocol/Other [a]	13	6	8
**Week 12 (+ unscheduled visits before)**				
No changes (target BP met)	Continue baseline regimen	93	103	35
	Continue 1st escalation	66	77	33
	Continue 2nd escalation	36	42	51
Dose escalation (target BP not met)	1st escalation	23	24	10
	2nd escalation	33	48	22
	Additional escalation	69	45	15
Other	Continued despite target BP not met	40	45	22
	Continue due to non-adherence	22	14	7
	Stop treatment due to AE	47	51	12
	Stop treatment due to pregnancy	2	4	0
	Not according protocol/Other [a]	17	11	12
**Week 24 (+ unscheduled visits before)**				
No changes (target BP met)	Continue baseline regimen	12	5	6
	Continue 1st escalation	23	14	10
	Continue 2nd escalation	12	25	13
	1st escalation	0	2	1
	2nd escalation	5	6	2
	Additional escalation	17	10	4
	Continued despite target BP not met	12	12	11
	Continue due to non-adherence	2	3	2
	Stop treatment due to AE	30	43	7
	Stop treatment due to pregnancy	4	1	0
	Not according protocol/Other [a]	69	53	23

CCB calcium-channel blocker; ARB angiotensin II receptor blocker; HCTZ hydrochlorothiazide;
[a] Not according protocol/Other were deviations from study protocol including not increasing dosage despite blood pressure target not met. AE adverse event; BP blood pressure.

© 2025 Mapesi H et al. *JAMA Cardiology*.

eTable 12. Treatment prescribed by visit week

	Stepped CCB monotherapy strategy (CCB/HCZT) N=505	Two pills strategy (CCB/ARB) N=510	Three pills strategy (CCB/ARB/HCTZ) N=253
**Week 0**	**505 (100 %)**	**510 (100 %)**	**253 (100 %)**
AML 10mg	505 (100 %)		
AML 5mg + LOS 50mg		510 (100 %)	
AML 2.5mg + LOS 12.5mg + HCZT 6.25mg			250 (98%)
Other			3 (1%) [1]
**Week 4**	**445**	**468**	**231**
AML 10mg	163	1	
AML 10mg + HCZT 25mg	262		
AML 5mg + LOS 50mg		182	
AML 10mg + LOS 50mg		279	
AML 2.5mg + LOS 12.5mg + HCZT 6.25mg			61
AML 5mg + LOS 25mg + HCZT 12.5mg			166
Other	19	6	3
None	1		1
**Week 8**	**428**	**447**	**223**
AML 10mg	130	2	
AML 10mg + HCZT 25mg	103		
AML 10mg + HCZT 50mg	161		
AML 5mg + LOS 50mg		134	
AML 10mg + LOS 50mg		146	
AML 10mg + LOS 100mg		149	
AML 2.5mg + LOS 12.5mg + HCZT 6.25mg			56
AML 5mg + LOS 25mg + HCZT 12.5mg			59
AML 10mg + LOS 50mg + HCZT 25mg			104
Other	34	16	2
None	0	0	2
**Week 12**	**448**	**454**	**219**
AML 10mg	117	3	
AML 10mg + HCZT 25mg	104		
AML 10mg + HCZT 50mg	114		
AML 5mg + LOS 50mg		122	
AML 10mg + LOS 50mg		108	
AML 10mg + LOS 100mg		140	
AML 2.5mg + LOS 12.5mg + HCZT 6.25mg			46
AML 5mg + LOS 25mg + HCZT 12.5mg			50
AML 10mg + LOS 50mg + HCZT 25mg			97
Other	111	79	26
None	2	2	0

CCB calcium-channel blocker; ARB angiotensin II receptor blocker; HCTZ hydrochlorothiazide; AML amlodipine; LOS losartan

© 2025 Mapesi H et al. *JAMA Cardiology*.

eTable 13. Adherence to study medication

	Stepped CCB monotherapy strategy (CCB/HCZT) N=505	Two pills strategy (CCB/ARB) N=510	Three pills strategy (CCB/ARB/HCTZ) N=253
**Week 4**			
Have adherence data	445	468	231
≥80% adherent	419 (94%)	438 (94%)	214 (93%)
≥90% adherent	377 (85%)	397 (85%)	187 (81%)
**Week 8**			
Have adherence data	428	447	223
≥80% adherent	386 (90%)	426 (95%)	206 (92%)
≥90% adherent	356 (83%)	393 (88%)	187 (84%)
**Week 12**			
Have adherence data	446	454	219
≥80% adherent	383 (86%)	406 (89%)	197 (90%)
≥90% adherent	342 (77%)	375 (83%)	173 (79%)
Odds ratio for ≥90% adherence			
Unadjusted OR (95% CI)	Reference	1.44 (1.04,2.00); p=0.03	1.14 (0.77,1.69); p=0.50
Adjusted OR (95% CI) [a]	Reference	1.45 (1.04,2.02); p=0.03	1.12 (0.75,1.66); p=0.59
**Week 24**			
Have adherence data	426	441	216
≥80% adherent	358 (84%)	376 (85%)	175 (81%)
≥90% adherent	315 (74%)	330 (75%)	153 (71%)
**Reasons indicated for missing medication in self-report**			
Number of visits with self-report of missing medication in past 4 weeks	187	163	96
Reason			
Forgot	116 (62%)	116 (71%)	69 (72%)
Lost drugs	6 (3%)	3 (2%)	0
Side effects	5 (3%)	3 (2%)	2 (2%)
Other	60 (32%)	41 (25%)	25 (26%)

CCB calcium-channel blocker; ARB angiotensin II receptor blocker; HCTZ hydrochlorothiazide;
Overall adherence was based on a combination of three methods of adherence measurement (self-report of missed medication, not taking medication as per instructions, pill count), with non-adherence by any measure prevailing. For the few participant-visits missing self-report information, we used the pill count information if available.
[a] From logistic regression model with arm, adjusted for site (Ifakara, Mokhotlong), HIV status (positive, negative), age (linear), baseline SBP (linear), and baseline DBP (linear).

© 2025 Mapesi H et al. *JAMA Cardiology*.

eTable 14. Adherence to medication by pill count

	Amlodipine			Losartan			Hydrochlorothiazide		
	**Stepped CCB monotherapy strategy (CCB/HCZT) N=505**	**Two pills strategy (CCB/ARB) N=510**	**Three pills strategy (CCB/ARB/HCTZ) N=253**	**Stepped CCB monotherapy strategy (CCB/HCZT) N=505**	**Two pills strategy (CCB/ARB) N=510**	**Three pills strategy (CCB/ARB/HCTZ) N=253**	**Stepped CCB monotherapy strategy (CCB/HCZT) N=505**	**Two pills strategy (CCB/ARB) N=510**	**Three pills strategy (CCB/ARB/HCTZ) N=253**
**Week 4**									
Have adherence data	442	468	231	0	468	230	0	0	231
≥80% adherent	417 (94%)	443 (95%)	215 (93%)	N/A	440 (94%)	215 (93%)	N/A	N/A	216 (94%)
≥90% adherent	384 (87%)	415 (89%)	193 (84%)	N/A	414 (88%)	197 (86%)	N/A	N/A	197 (85%)
**Week 8**									
Have adherence data	414	445	221	1	445	223	247	0	223
≥80% adherent	374 (90%)	427 (96%)	206 (93%)	1 (100 %)	426 (96%)	211 (95%)	237 (96%)	N/A	209 (94%)
≥90% adherent	357 (86%)	400 (90%)	191 (86%)	1 (100 %)	400 (90%)	193 (87%)	219 (89%)	N/A	194 (87%)
**Week 12**									
Have adherence data	424	445	218	4	451	219	292	5	218
≥80% adherent	370 (87%)	398 (89%)	196 (90%)	4 (100 %)	405 (90%)	198 (90%)	258 (88%)	5 (100 %)	198 (91%)
≥90% adherent	346 (82%)	376 (84%)	180 (83%)	2 (50%)	383 (85%)	180 (82%)	232 (79%)	5 (100 %)	181 (83%)
**Week 24**									
Have adherence data	400	419	213	76	438	216	301	61	216
≥80% adherent	343 (86%)	362 (86%)	178 (84%)	71 (93%)	378 (86%)	181 (84%)	264 (88%)	54 (89%)	180 (83%)
≥90% adherent	319 (80%)	330 (79%)	159 (75%)	67 (88%)	345 (79 %)	162 (75%)	239 (79%)	50 (82%)	159 (74%)

CCB calcium-channel blocker; ARB angiotensin II receptor blocker; HCTZ hydrochlorothiazide;

© 2025 Mapesi H et al. *JAMA Cardiology*.

eTable 15. Left ventricular hypertrophy

	Stepped CCB monotherapy strategy (CCB/HCZT) N=505	Two pills strategy (CCB/ARB) N=510	Three pills strategy (CCB/ARB/HCTZ) N=253
**Echocardiography**			
**Week 0**			
Left ventricular mass index	65 (57-76)	67 (56-77)	65 (56-78)
Index above threshold [a]	27 (6%)	16 (4%)	14 (6%)
Missing	44 (9%)	53 (10%)	24 (9%)
**Week 24**			
Left ventricular mass index	61 (52-70)	63 (54-73)	62 (54-72)
Index above threshold [a]	8 (2%)	4 (1%)	5 (3%)
Missing	134 (27%)	111 (22%)	57 (23%)
**Electrocardiography**			
**Week 0**			
MESA ECG LVH, mV	4 (3-5)	4 (3-5)	4 (4-5)
ECG LVH criterion reached [b]	274 (56%)	278 (56%)	141 (56%)
Missing	12 (2%)	11 (2%)	2 (1%)
**Week 24**			
MESA ECG LVH, mV	4 (3-5)	4 (3-5)	4 (3-5)
ECG LVH criterion reached [b]	148 (36%)	166 (39%)	72 (35%)
Missing	98 (19%)	86 (17%)	46 (18%)

CCB calcium-channel blocker; ARB angiotensin II receptor blocker; HCTZ hydrochlorothiazide; MESA, multi ethnic study of atherosclerosis; ECG electrocardiogram; LVH left ventricular hypertrophy. Results are number (column % of those with non-missing data; missing % are of those randomized) for categorical variables and median (interquartile range) for continuous variables.
[a] >95 g/m^2 in women, >115 g/m^2 in men.
[b] SV1+SV2+RV5 >=4.2 mV.
[c] If available at both time points, results from echocardiography were used, if not MESA score from electrocardiography was calculated, if both available we graded according the more pathologic result.

© 2025 Mapesi H et al. *JAMA Cardiology*.

eTable 16. Renal impairment

	Stepped CCB monotherapy strategy (CCB/HCZT) N=505	Two pills strategy (CCB/ARB) N=510	Three pills strategy (CCB/ARB/HCTZ) N=253
**Week 0**			
eGFR, mL/min/1.73 m2	97 (81-113)	97 (79 -115)	95 (77-113)
Missing	43 (9%)	54 (11%)	30 (12%)
KDIGO eGFR category			
G1	283 (61%)	266 (58%)	126 (57%)
G2	146 (32%)	157 (34%)	77 (35%)
G3a	23 (5%)	25 (5%)	15 (7%)
G3b	10 (2%)	7 (2%)	5 (2%)
G4	0 (0%)	1 (0%)	0 (0%)
Missing	43 (9%)	54 (11%)	30 (12%)
KDIGO eGFR summarised categories			
G1-G2 (≥60 ml/min/1.73 m3)	429 (93%)	423 (93%)	203 (91%)
G3a-G5 (<60 ml/min/1.73 m3)	33 (7 %)	33 (7%)	20 (9%)
Missing	43 (9%)	54 (11%)	30 (12%)
KDIGO albuminuria category (ACR)			
A1a	187 (38%)	187 (39%)	95 (39%)
A1b	168 (34%)	175 (37%)	85 (35%)
A2	100 (21%)	80 (17%)	50 (21%)
A3	32 (7%)	37 (8%)	12 (5%)
Missing	18 (4%)	31 (6%)	11 (4%)
**Week 24**			
eGFR, mL/min/1.73 m2	93 (75-108)	92 (73-108)	87 (70-105)
Missing	116 (23%)	102 (20%)	58 (23%)
KDIGO eGFR category			
G1	208 (53%)	217 (53%)	88 (45%)
G2	146 (38%)	151 (37%)	86 (44%)
G3a	29 (7%)	29 (7%)	10 (5%)
G3b	4 (1%)	11 (3%)	11 (6%)
G4	2 (1%)	0 (0%)	0 (0%)
Missing	116 (23%)	102 (20%)	58 (23%)
KDIGO eGFR summarised categories			
G1-G2 (>=60 ml/min/1.73 m3)	354 (91%)	368 (90%)	174 (89%)
G3a-G5 (<60 ml/min/1.73 m3)	35 (9%)	40 (10%)	21 (11%)
Missing	116 (23%)	102 (20%)	58 (23%)
KDIGO albuminuria category (ACR)			
A1a	181 (45%)	229 (54%)	113 (55%)
A1b	157 (39%)	129 (30%)	59 (29%)
A2	49 (12%)	50 (12%)	26 (13%)
A3	13 (3%)	19 (4%)	7 (3%)
Missing	105 (21%)	83 (16%)	48 (19%)
**Renal impairment by eGFR**			
never	313 (88%)	323 (89%)	146 (84%)
newly occurring	16 (5%)	15 (4%)	10 (6%)
worsening	4 (1%)	3 (1%)	5 (3%)
resolving	17 (5%)	10 (3%)	9 (5%)

© 2025 Mapesi H et al. *JAMA Cardiology*.

no change	4 (1%)	11 (3%)	3 (2%)
Missing	151 (30%)	148 (29%)	80 (32%)
**Renal impairment by albuminuria (ACR)**			
never	208 (54%)	221 (55%)	109 (56%)
newly occurring	25 (6%)	22 (5%)	10 (5%)
worsening	1 (0%)	3 (1%)	0 (0%)
resolving	80 (21%)	70 (17%)	35 (18%)
no change	73 (19%)	87 (22%)	41 (21%)
Missing	118 (23%)	107 (21%)	58 (23%)

CCB calcium-channel blocker; ARB angiotensin II receptor blocker; HCTZ hydrochlorothiazide; eGFR estimated glomerular filtration rate; ACR albumin creatinine ratio. KDIGO Kidney Disease Improving Global Outcomes.[2] Results are number (column % of those with non-missing data; missing % are of those randomized) for categorical variables and median (interquartile range) for continuous variables. Of note, a total 65 ACR results were excluded due to the participant having a urinary tract infection. a Definitions of renal impairment see Table S2. If both eGFR and ACR were available at baseline and follow-up, the more pathologic evaluation determined the outcome, if only one was available, this was used.

© 2025 Mapesi H et al. *JAMA Cardiology*.

eTable 17. Post-hoc analysis of effect modification by age

	**Stepped CCB monotherapy strategy (CCB/HCZT) N=505**	**Two pills strategy (CCB/ARB) N=510**	**Three pills strategy (CCB/ARB/HCTZ) N=253**
**Complete case analysis**			
Number included			
<65 years	329	341	166
≥65 years	118	112	53
Number reached primary outcome (%) [a]			
<65 years	136 (41%)	175 (51%)	90 (54%)
≥65 years	93 (79%)	82 (73%)	41 (77%)
Adjusted odds ratio (95% CI) [b]			
<65 years	Reference	1.49 (1.09,2.05)	1.58 (1.07,2.32)
≥65 years	Reference	0.64 (0.34,1.19)	0.92 (0.41,2.06)
P value for effect modification	-	0.02	0.24
**Multiple imputation analysis**			
Number included			
<65 years	379	382	190
≥65 years	122	123	62
% reached primary outcome [a]			
<65 years	39%	49%	51%
≥65 years	78%	73%	74%
Adjusted odds ratio (95% CI) [b]			
<65 years	Reference	1.50 (1.10,2.06)	1.55 (1.06,2.29)
≥65 years	Reference	0.67 (0.36,1.23)	0.82 (0.38,1.81)
P value for effect modification	-	0.02	0.16

CCB calcium-channel blocker; ARB angiotensin II receptor blocker; HCTZ hydrochlorothiazide;
[a] Percentages of those included in the respective analysis.
[b] From logistic regression model with arm, adjusted for site, HIV status, age (linear), baseline SBP (linear), and baseline DBP (linear).

© 2025 Mapesi H et al. *JAMA Cardiology*.

eTable 18. Essential target blood pressure attainment[6]

	Stepped CCB monotherapy strategy (CCB/HCZT) N=505	Two pills strategy (CCB/ARB) N=510	Three pills strategy (CCB/ARB/HCTZ) N=253
**Complete case analysis**			
Number included	447	453	219
Number reached outcome (%) [a]	342 (77%)	349 (77%)	179 (82%)
Unadjusted odds ratio (95% CI)	Reference	1.03 (0.76,1.40); p=0.85	1.37 (0.91,2.06); p=0.13
Adjusted odds ratio (95% CI) [b]	Reference	0.97 (0.70,1.34); p=0.84	1.30 (0.85,1.99); p=0.22
**Multiple imputation analysis**			
Number included	501	505	252
% reached outcome [b]	75%	76%	79%
Unadjusted odds ratio (95% CI)	Reference	1.01 (0.75,1.37); p=0.95	1.22 (0.83,1.80); p=0.32
Adjusted odds ratio (95% CI) [b]	Reference	0.96 (0.70,1.32); p=0.82	1.18 (0.79,1.78); p=0.42

CCB calcium-channel blocker; ARB angiotensin II receptor blocker; HCTZ hydrochlorothiazide;
[a] Percentages of those included in the respective analysis.
[b] From logistic regression model with arm, adjusted for site, HIV status, age (linear), baseline SBP (linear), and baseline DBP (linear).

© 2025 Mapesi H et al. *JAMA Cardiology*.

eFigure 1. Time to achieve target blood pressure

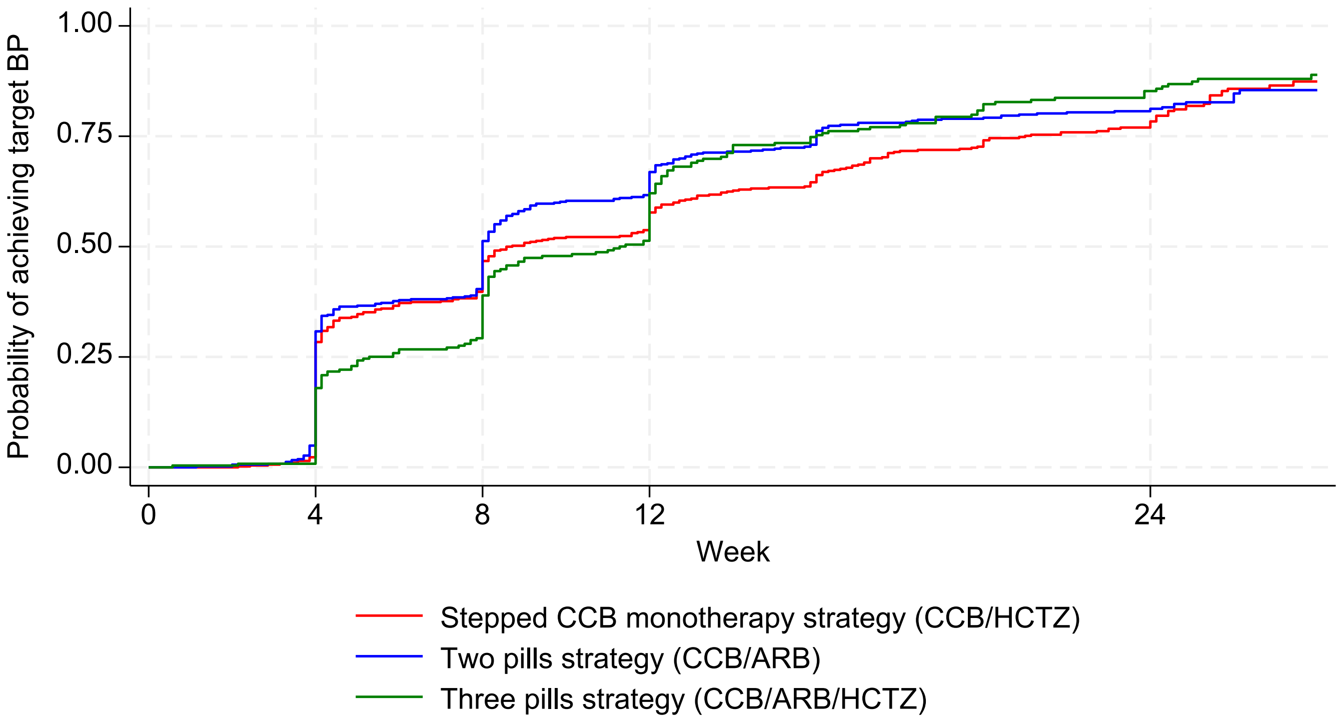

Target blood pressure defined as per primary outcome (≤130/80mmHg for participants aged <65 years, ≤140/90mmHg for those aged ≥65 years). CCB calcium-channel blocker; ARB angiotensin II receptor blocker; HCTZ hydrochlorothiazide; BP blood pressure.

© 2025 Mapesi H et al. *JAMA Cardiology*.

eFigure 2. Treatment prescribed by visit week

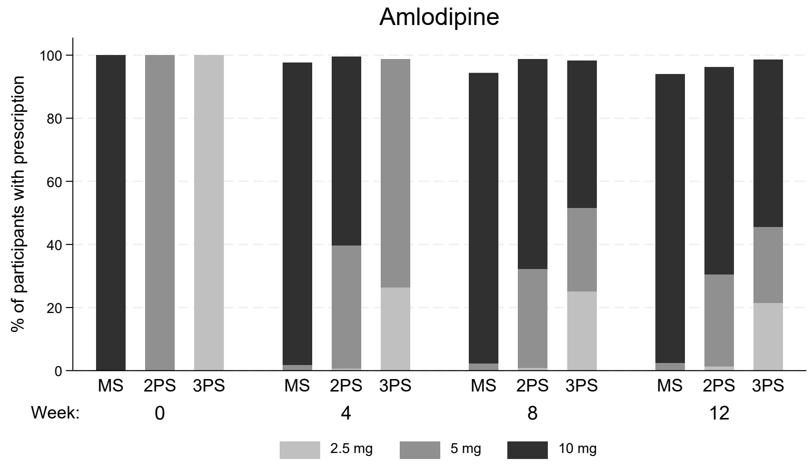

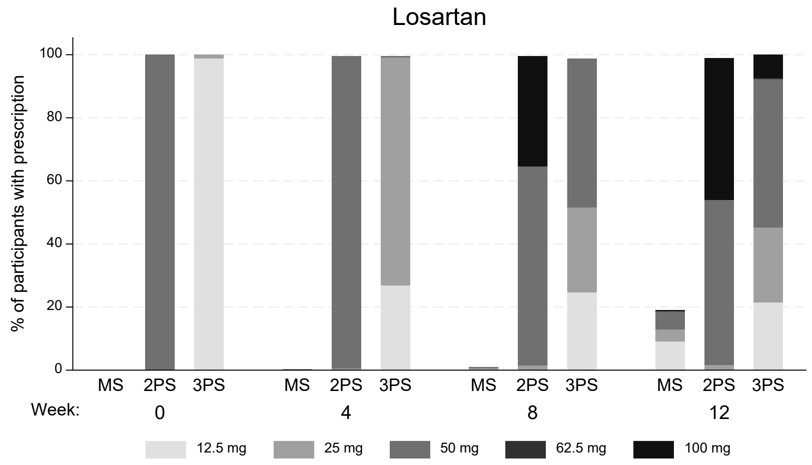

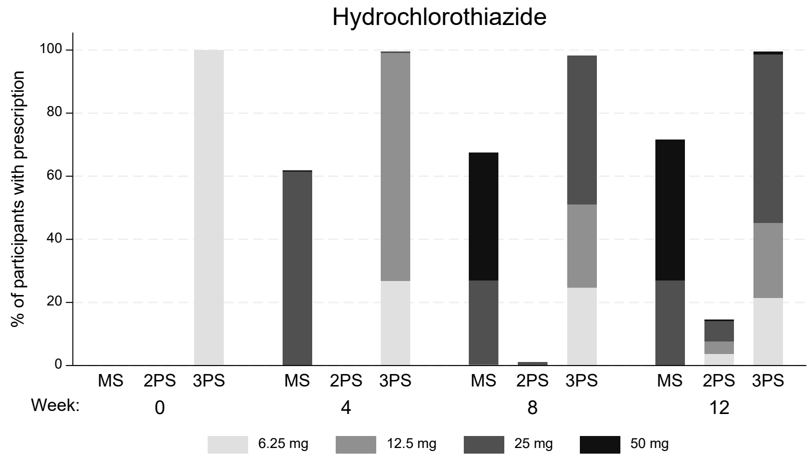

Bars indicate antihypertensive drugs with different dosages prescribed at a given visit. MS=stepped CCB monotherapy strategy (CCB/HCTZ), 2PS=two pills strategy (CCB/ARB), 3PS=three pills strategy (CCB/ARB/HCTZ).
CCB calcium-channel blocker; ARB angiotensin II receptor blocker; HCTZ hydrochlorothiazide

© 2025 Mapesi H et al. *JAMA Cardiology*.

eFigure 3A-B. Blood pressure target attainment

A) Participants < 65years

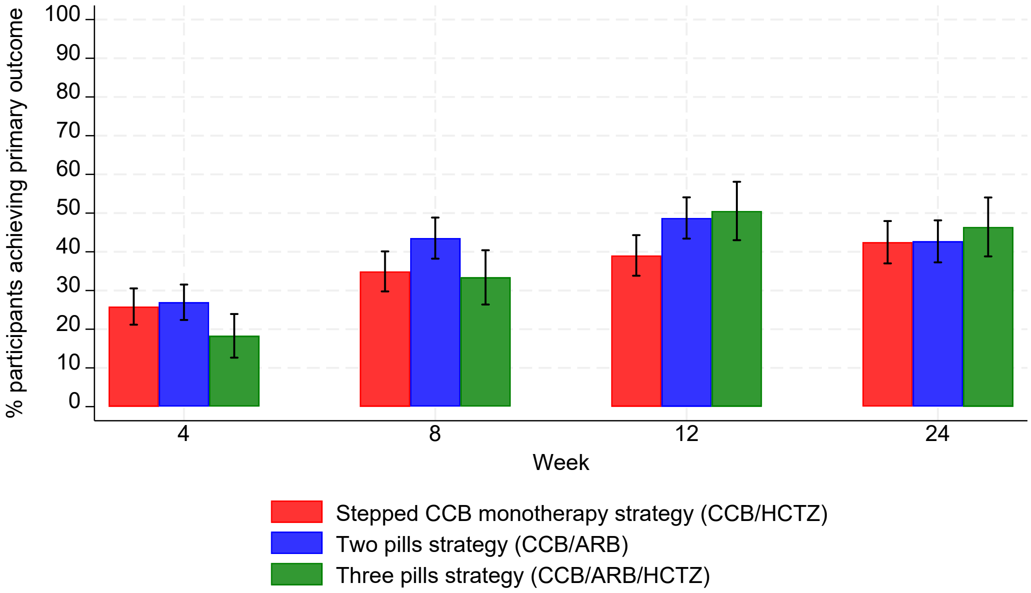

B) Participants ≥65 years

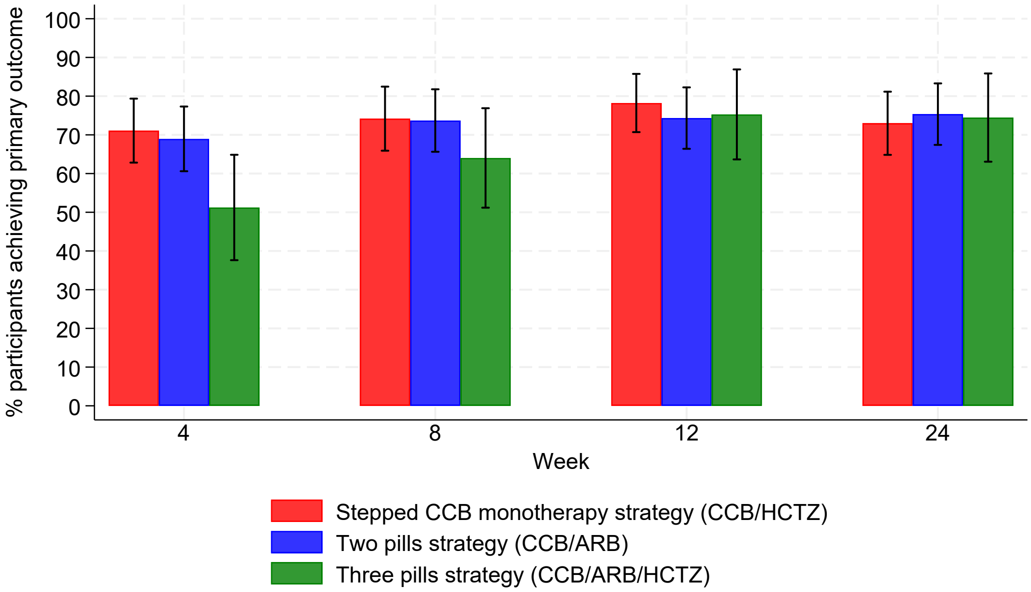

Analysis based on multiply imputed data. The primary outcome of target blood pressure (defined as SBP ≤130 mmHg and DBP ≤80 mmHg for participants aged <65 years and SBP ≤140 mmHg and DBP ≤90 mmHg for participants aged ≥65 years) is shown in bars at week 12 and additionally for secondary outcomes at week 4, 8 and 24 for those aged <65 years (A) and for those aged ≥65 years. SBP systolic blood pressure; DBP diastolic blood pressure; CCB calcium-channel blocker; ARB angiotensin II receptor blocker; HCTZ hydrochlorothiazide;

© 2025 Mapesi H et al. *JAMA Cardiology*.

eReferences

1. Use EMAECfMPfH. ICH E9 (R1) addendum on estimands and sensitivity analysis in clinical trials to the guideline on statistical principles for clinical trials. , 2017.
2. National Kidney F. K/DOQI clinical practice guidelines for chronic kidney disease: evaluation, classification, and stratification. *Am J Kidney Dis* 2002; **39**(2 Suppl 1): S1-266.
3. Levey AS, Stevens LA, Schmid CH, et al. A new equation to estimate glomerular filtration rate. *Ann Intern Med* 2009; **150**(9): 604-12.
4. Lang RM, Badano LP, Mor-Avi V, et al. Recommendations for cardiac chamber quantification by echocardiography in adults: an update from the American Society of Echocardiography and the European Association of Cardiovascular Imaging. *J Am Soc Echocardiogr* 2015; **28**(1): 1-39 e14.
5. Jain A, Tandri H, Dalal D, et al. Diagnostic and prognostic utility of electrocardiography for left ventricular hypertrophy defined by magnetic resonance imaging in relationship to ethnicity: the Multi-Ethnic Study of Atherosclerosis (MESA). *Am Heart J* 2010; **159**(4): 652-8.
6. Unger T, Borghi C, Charchar F, et al. 2020 International Society of Hypertension Global Hypertension Practice Guidelines. *Hypertension* 2020; **75**(6): 1334-57.

© 2025 Mapesi H et al. *JAMA Cardiology*.
