# Supplementary material for: Treatment Strategies to Control Blood Pressure in People With Hypertension in Tanzania and Lesotho: A Randomized Clinical Trial
Source: JAMA Cardiol. 2025 Jan 29;10(4):321–33. doi: 10.1001/jamacardio.2024.5124 (PMC11780507; doi:10.1001/jamacardio.2024.5124)
Supplement: Supplement 4. — Data Sharing Statement. [file jamacardiol-e245124-s004.pdf]

Data Sharing Statement

Mapesi. Treatment Strategies to Control Blood Pressure in People With Hypertension in Tanzania and Lesotho. *JAMA Cardiol*. Published January 29, 2025.
doi:10.1001/jamacardio.2024.5124

Data
Additional Information: clinicaltrials.gov; NCT04129840;
https://www.clinicaltrials.gov/study/NCT04129840
Data available: Yes
Data types: Deidentified participant data
How to access data: Zenodo (DOI: 10.5281/zenodo.11090818; pending publication)
When available: beginning date: 08-28-2024

Supporting Documents
Document types: None

Additional Information
Who can access the data: researchers whose proposed use of the data has been approved
Types of analyses: specified purposes, e.g. meta-analyses
Mechanisms of data availability: without investigator support
